# Metabolic network capacity of *Escherichia coli* for Krebs cycle-dependent proline hydroxylation

**DOI:** 10.1186/s12934-015-0298-1

**Published:** 2015-07-29

**Authors:** Eleni Theodosiou, Oliver Frick, Bruno Bühler, Andreas Schmid

**Affiliations:** Department of Biochemical and Chemical Engineering, Laboratory of Chemical Biotechnology, TU Dortmund University, Emil-Figge-Strasse 66, 44227 Dortmund, Germany; Department of Solar Materials, Helmholtz-Centre for Environmental Research - UFZ, Leipzig, Germany

**Keywords:** Whole-cell biocatalysis, Proline hydroxylation, Strain engineering, Metabolic flux analysis, Biocatalyst efficiency

## Abstract

**Background:**

Understanding the metabolism of the microbial host is essential for the development and optimization of whole-cell based biocatalytic processes, as it dictates production efficiency. This is especially true for redox biocatalysis where metabolically active cells are employed because of the cofactor/cosubstrate regenerative capacity endogenous in the host. Recombinant *Escherichia coli* was used for overproducing proline-4-hydroxylase (P4H), a dioxygenase catalyzing the hydroxylation of free l-proline into *trans*-4-hydroxy-l-proline with *a*-ketoglutarate (*a*-KG) as cosubstrate. In this whole-cell biocatalyst, central carbon metabolism provides the required cosubstrate *a*-KG, coupling P4H biocatalytic performance directly to carbon metabolism and metabolic activity. By applying both experimental and computational biology tools, such as metabolic engineering and ^13^C-metabolic flux analysis (^13^C-MFA), we investigated and quantitatively described the physiological, metabolic, and bioenergetic response of the whole-cell biocatalyst to the targeted bioconversion and identified possible metabolic bottlenecks for further rational pathway engineering.

**Results:**

A proline degradation-deficient *E. coli* strain was constructed by deleting the *putA* gene encoding proline dehydrogenase. Whole-cell biotransformations with this mutant strain led not only to quantitative proline hydroxylation but also to a doubling of the specific *trans*-4-l-hydroxyproline (hyp) formation rate, compared to the wild type. Analysis of carbon flux through central metabolism of the mutant strain revealed that the increased *a*-KG demand for P4H activity did not enhance the *a*-KG generating flux, indicating a tightly regulated TCA cycle operation under the conditions studied. In the wild type strain, P4H synthesis and catalysis caused a reduction in biomass yield. Interestingly, the Δ*putA* strain additionally compensated the associated ATP and NADH loss by reducing maintenance energy demands at comparably low glucose uptake rates, instead of increasing the TCA activity.

**Conclusions:**

The *putA* knockout in recombinant *E. coli* BL21(DE3)(pLysS) was found to be promising for productive P4H catalysis not only in terms of biotransformation yield, but also regarding the rates for biotransformation and proline uptake and the yield of hyp on the energy source. The results indicate that, upon a *putA* knockout, the coupling of the TCA-cycle to proline hydroxylation via the cosubstrate *a*-KG becomes a key factor constraining and a target to further improve the efficiency of *a*-KG-dependent biotransformations.

**Electronic supplementary material:**

The online version of this article (doi:10.1186/s12934-015-0298-1) contains supplementary material, which is available to authorized users.

## Background

Fe(II)⁄*α*-ketoglutarate-dependent dioxygenases are nonheme iron-containing oxygenases coupling dioxygen activation to the oxidative decarboxylation of *α*-ketoglutarate (*α*-KG). These enzymes catalyze a remarkable range of reactions, such as hydroxylation, desaturation, and epoxidation [1, 2]. In the hydroxylation reaction catalyzed by proline-4-hydroxylase (P4H), one atom of molecular oxygen is introduced into l-proline to give *trans*-4-hydroxy-l-proline (hyp), a valuable intermediate for the synthesis of chiral pharmaceuticals and antibiotics [3], while the other oxygen atom is introduced into the cosubstrate *α*-KG to give succinate and CO_2_ upon oxidative decarboxylation [4].

**Fig. 1:**
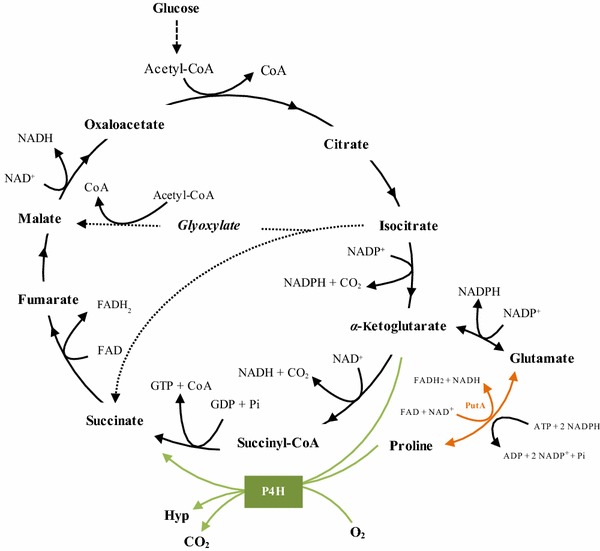
Schematic representation of the interconnection between *trans*-4-hydroxy-l-proline (hyp) synthesis catalyzed by recombinant *Escherichia coli* containing proline-4-hydroxylase (P4H) and central carbon metabolism.

For an economically viable process that utilizes P4H or other *α*-KG-dependent dioxygenases, microbial cells are the preferred biocatalysts, since *α*-KG is a central metabolite and can be supplied continuously by host metabolism, namely the tricarboxylic acid (TCA) cycle. Thus, the biocatalytic target reaction creates a short-cut in the TCA cycle that intertwines whole-cell catalytic efficiency with microbial metabolism and physiology (Fig. 1). When using whole-cell biocatalysts, numerous physiology-related factors can potentially interfere with catalytic performance, i.e., limited substrate uptake or cosubstrate supply, substrate or product metabolization by host intrinsic enzymes, by-product formation, and toxicity of substrates and products [5–7]. Therefore, retrofitting of cellular functionalities is often necessary to overcome unwanted constraints and to obtain efficient and robust microbial cell factories specifically tailored to meet commercial biotechnological objectives [8, 9].

For the synthesis of hyp from proline, a commercially used whole-cell process has already been reported [10]. However, the interdependency of process conditions, host metabolism, and catalyst performance has not been unraveled yet. Using P4H containing recombinant *Escherichia coli* cells, it was recently demonstrated that there is a strong interference of catalytic activity with the regulation of proline uptake and metabolism [11]. In *E. coli,* proline degradation requires two genes, *putP* and *putA*, encoding the Na^+^/proline symport carrier (PutP) and the multifunctional proline utilization protein A (PutA), respectively [12]. Depending on proline availability, PutA functions either as a DNA-binding transcriptional repressor of *putA* and *putP* expression (low proline levels) or as a membrane-bound bi-functional dehydrogenase responsible for the two-step oxidation of proline to glutamate (high proline levels). PutA is thus involved in both, proline catabolism and its transcriptional regulation [13]. For biocatalytically active growing cells, a catalysis-induced reduction of proline uptake was observed, which correlated with reduced transcription of *putA* and *putP*, demonstrating that proline uptake and competition of hyp formation from proline by P4H with proline catabolism were the key factors limiting biocatalyst efficiency [11].

In this study, *E. coli* BL21(DE3)(pLysS), a potent host strain for P4H catalysis ([11], hereafter referred to as wt), overexpressing a codon-optimized *p4h* gene (*p4h1of*) was rationally engineered to further comprehend and modulate the interplay between cellular physiology and proline hydroxylation. Toward this goal, a knockout mutant lacking the *putA* gene (*E. coli* BL21Δ*putA*(DE3)(pLysS), referred to as Δ*putA*) was constructed and physiologically characterized. This mutation eliminated both proline oxidation to glutamate and transcriptional repression of the *put* operon. Using ^13^C-based metabolic flux analysis (^13^C-MFA), the metabolic response of the whole-cell catalyst upon genetic (i.e., *putA* deletion, *p4h1of* expression) and environmental perturbations (i.e., proline addition, product synthesis) was assessed. Furthermore, energy and redox metabolism were investigated via ^13^C-MFA to reveal how the cells attune their bioenergetic status upon product synthesis and under various growth conditions.

## Results and discussion

### Proline degradation deficiency increases both hyp yield and formation rate

To determine how proline metabolism and its regulation affect the physiology of a proline-hydroxylating biocatalyst, the impact of the *putA* deletion on exponential growth parameters and hyp synthesis was investigated. The engineered strain, bearing either pET-24a (Δ*putA*_pET) or pET_p4h1of (Δ*putA_*p4h1of), was incubated aerobically in M9 minimal medium with glucose as carbon and energy source in the presence or absence of proline and compared with the wildtype strain cultivated under the same conditions (Table 1).

**Table 1:** Physiological comparison of aerobically growing recombinant *E. coli* BL21(DE3)(pLysS) strains

	wt_pET		Δ*putA*_pET		wt_p4h1of		Δ*putA*_p4h1of	
	−Pro	+Pro	−Pro	+Pro	−Pro	+Pro	−Pro	+Pro
**μ** (h^−1^)	0.44 ± 0.01	0.48 ± 0.01	0.42 ± 0.01	0.43 ± 0.01	0.32 ± 0.02	0.33 ± 0.00	0.3 ± 0.01	0.32 ± 0.01
Final biomass (g_CDW_ L^−1^)	1.9 ± 0.1	2.2 ± 0.1	2.0 ± 0.1	2.0 ± 0.1	1.4 ± 0.1	1.8 ± 0.1	1.5 ± 0.1	1.5 ± 0.1
***r***_Glucose_ (mmol g^−1^ h^−1^)	−5.3 ± 0.3	−5.1 ± 0.1	−5.2 ± 0.2	−5.1 ± 0.2	−4.8 ± 0.3	−4.4 ± 0.3	−4.7 ± 0.2	−3.9 ± 0.1
***r***_Acetate_ (mmol g h^−1^)	1.33 ± 0.1	1.59 ± 0.2	0.98 ± 0.1	0.76 ± 0.1	0.92 ± 0.1	1.24 ± 0.1	1.09 ± 0.04	0.93 ± 0.1
***r***_Proline_ (mmol g h^−1^)	NA	−1.08 ± 0.01	NA	ND	NA	−0.68 ± 0.01	NA	−0.75 ± 0.01
***r***_Hyp_ (mmol g h^−1^)	ND	ND	ND	ND	0.09 ± 0.01	0.37 ± 0.01	0.14 ± 0.01	0.75 ± 0.01
***Y***_x/glc_ (g_CDW_ g_glc_^−1^)	0.46 ± 0.02	0.52 ± 0.01	0.45 ± 0.02	0.47 ± 0.01	0.39 ± 0.02	0.42 ± 0.03	0.36 ± 0.01	0.45 ± 0.01
***Y***_ace/glc_ (mol_ace_ mol_glc_^−1^)	0.25 ± 0.03	0.31 ± 0.04	0.19 ± 0.03	0.15 ± 0.01	0.19 ± 0.01	0.28 ± 0.01	0.23 ± 0.01	0.24 ± 0.03
***Y***_hyp/pro_ (mol_hyp_ mol_pro_^−1^)	NA	ND	NA	ND	NA	0.55 ± 0.01	NA	1 ± 0.01
***Y***_hyp/glc_ (mol_hyp_ mol_glc_^−1^)	ND	ND	ND	ND	0.019 ± 0.001	0.084 ± 0.003	0.030 ± 0.001	0.192 ± 0.002
***r***_Hyp_ (U g_CDW_^−1^)	NA	NA	NA	NA	1.35 ± 0.02	6.1 ± 0.2	2.20 ± 0.02	12.6 ± 0.1

Cultivation was performed at 30°C in M9 medium with 5 g L^−1^ glucose in the absence (−Pro) or presence (+Pro) of 5 mM proline. wt_pET, *E. coli* BL21(DE3)(pLysS)(pET-24a); Δ*putA*_pET, *E. coli* BL21Δ*putA*(DE3)(pLysS)(pET-24a); wt_p4h1of, *E. coli* BL21(DE3)(pLysS)(pET_p4h1of); Δ*putA*_p4h1of, *E. coli* BL21Δ*putA*(DE3)(pLysS)(pET_p4h1of); μ, specific growth rate; r, specific rates for substrate uptake (negative values) or product formation; CDW, cell dry weight; pro, proline; ace, acetate; hyp, *trans*-4-hydroxy-l-proline; Y_x/glc_, Y_ace/glc_, Y_hyp/pro_, and Y_hyp/glc_, yield coefficients for biomass and acetate on glucose and for hyp on proline and glucose, respectively; ND, not detected; NA, not applicable; 1 U = 1 μmol of product formed per min. Values represent the average from three different experiments (biological replicates) with standard deviations displayed as error.

In case of pET-24a containing wildtype cells (wt_pET) grown in the presence of proline, PutA associates with the membrane, catalyzing the first steps of proline degradation to glutamate [12]. Glutamate is subsequently deaminated to the TCA cycle intermediate *α*-KG, supplying additional carbon, nitrogen, and energy to the cells. This ability of the wildtype to catabolize proline together with glucose led to an increase in growth rate, final biomass titer, and biomass yield on glucose, compared to growth on glucose alone. On the contrary, the mutant strain Δ*putA* was, as expected, unable to degrade proline. The specific growth rate (μ), final biomass titer, and biomass yield on glucose of the Δ*putA_*pET strain were virtually unchanged upon proline addition and resembled the wt_pET strain grown on glucose (Table 1, see also Additional file 1: Figure S1). Similarly, proline addition had no effect on the growth rate of the Δ*putA_*p4h1of strain. Interestingly, the presence of proline also did not significantly influence the growth rate of the wildtype strain containing pET_p4h1of (wt_p4h1of). This can be explained by the reduced amount of proline used for biomass formation due to hydroxylation, and the decreased proline uptake rate induced by P4H activity [11]. Nevertheless, proline addition increased the final biomass titer of the wt_p4h1of strain.

In all cases, both growth rate and biomass formed were decreased, when P4H was produced, indicating a metabolic burden imposed by heterologous P4H synthesis [14]. The glucose uptake rate and the biomass yield on glucose were also reduced upon P4H synthesis, independently of proline addition, indicating that less energy and metabolite precursors are used for biomass formation. It should be noted that P4H was successfully produced at constant levels under all conditions studied (see Additional file 1: Figure S2) and that, in the absence of extracellular proline, both wt_p4h1of and Δ*putA*_p4h1of could synthesize hyp from endogenous proline. Interestingly, upon proline addition, the Δ*putA*_p4h1of strain displayed the lowest glucose uptake rate (3.9 mmol g^−1^ h^−1^), but its biomass yield on glucose (0.45 g_CDW_ g_glc_^−1^) was comparably high, suggesting energy-efficient biomass formation.

Acetate was secreted by all strains under the conditions studied, implying an excess of available acetyl-CoA as a result of the TCA cycle not keeping pace with glycolysis [15, 16], and was assimilated after glucose depletion. The specific growth rate was not negatively affected by the relatively low acetate concentrations (from 0.1 to 0.26 g L^−1^, see Additional file 1: Figures S1 and S3), which were below the reported growth-inhibiting acetate concentrations of 0.5–5 g L^−1^ [17, 18]. While the presence of proline resulted in an increased acetate formation rate in the case of wt_pET and wt_p4h1of, no such effect was observed for the corresponding Δ*putA* strains. Thus, proline-derived carbon influx into the central metabolism appears to promote overflow metabolism. Interestingly, the Δ*putA*_pET strain showed less acetate formation as the only difference to the wt_pET strain indicating a higher carbon cost of maintenance. However, in the absence of PutA, P4H synthesis (Δ*putA*_p4h1of) caused an increase in acetate yield on glucose, probably due to the metabolic stress.

Overall, proline hydroxylation profited from the following beneficial effects of *putA* deletion: (1) quantitative transformation of proline into hyp, (2) doubling of the specific hyp formation rate, and (3) a 2.3-fold higher molar hyp yield on glucose, characterizing this strain as a favorable biocatalyst for proline hydroxylation (Table 1, see also Additional file 1: Figure S3).

### Metabolic network operation: *α*-KG formation via the TCA cycle does not increase upon P4H catalysis

**Fig. 2:**
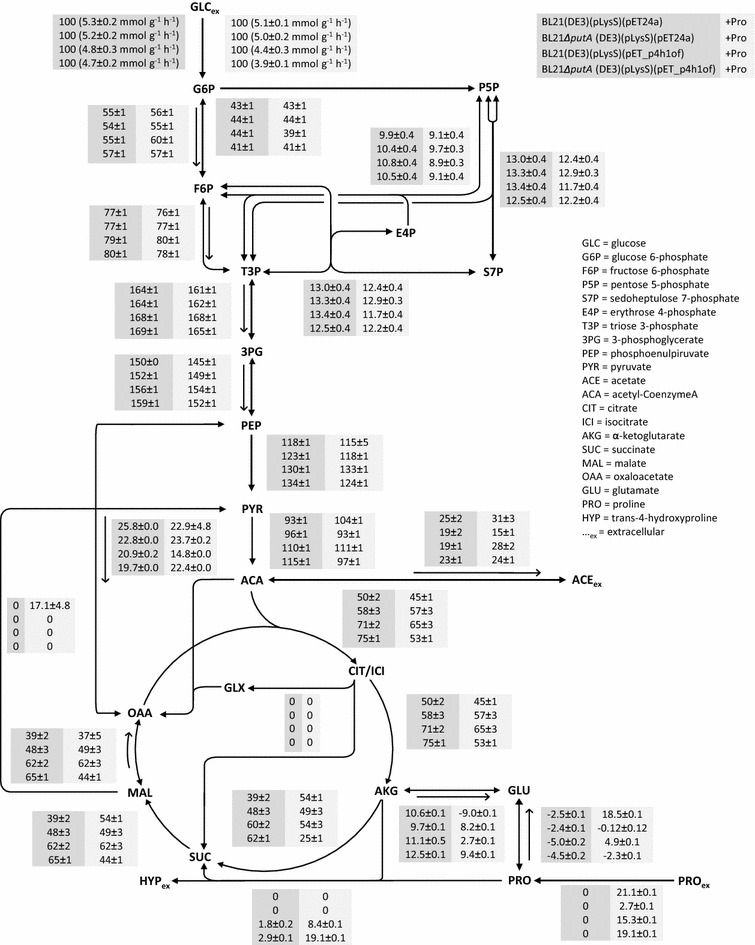
Metabolic fluxes in recombinant *E. coli* BL21(DE3)(pLysS) and *E. coli* BL21Δ*putA*(DE3)(pLysS) strains containing pET-24a or pET_p4h1of during exponential aerobic growth at 30°C in M9 medium containing 5 g L^−1^ glucose (4:1 mixture of 1-^13^C-labeled and U-^13^C-labeled glucose) in the absence or presence of 5 mM proline. All fluxes are given as relative fluxes normalized to the specific glucose uptake rate of 100 for each strain (given for the reaction of glucose to glucose-6-phosphate). For the extracellular metabolite fluxes, the experimental error is given. *Arrows* indicate the main direction of reversible reactions.

To investigate how the *putA* deletion and hyp synthesis affect the operation of the intracellular reaction network, ^13^C-MFA was performed for the wildtype and the Δ*putA* strain, bearing either pET-24a or pET_p4h1of, during exponential batch growth in the presence and absence of proline in M9 medium containing labeled glucose (80% [1-^13^C] and 20% [U-^13^C]). The relative carbon flux distributions throughout the central metabolic pathways for all strains and conditions studied, normalized to the glucose uptake rate, are mapped in Fig. 2.

Flux patterns in the upper part of glycolysis (until phosphoenolpyruvate) were similar for all strains and were virtually unaffected by the presence of proline. When using *putA*-positive strains, unlabeled proline-derived carbon only became evident in amino acids synthesized from TCA cycle intermediates (Table 2). As expected for Δ*putA* strains, which are unable to channel proline carbon into central metabolism, the unlabeled carbon content in amino acids (except for proline) did not change upon proline addition.

**Table 2:** Fractional abundance of unlabeled amino acid fragments (*m*0) during cultivation on labeled glucose with and without unlabeled proline

	wt_pET		Δ*putA*_pET		wt_p4h1of		Δ*putA*_p4h1of	
	−Pro	+Pro	−Pro	+Pro	−Pro	+Pro	−Pro	+Pro
Glycolysis								
Ala [M-57]^+^	0.366	0.377	0.366	0.368	0.365	0.360	0.361	0.359
Gly [M-57]^+^	0.622	0.624	0.621	0.622	0.621	0.621	0.619	0.620
Val [M-57]^+^	0.175	0.183	0.176	0.175	0.174	0.169	0.169	0.165
Leu [M-159]^+^	0.113	0.12	0.114	0.114	0.113	0.105	0.109	0.104
Ser [M-57]^+^	0.319	0.322	0.321	0.321	0.313	0.311	0.313	0.310
Phe [M-57]^+^	0.111	0.114	0.111	0.110	0.113	0.109	0.109	0.108
TCA								
Asp [M-57]^+^	0.195	0.336	0.194	0.194	0.191	0.233	0.187	0.179
Thr [M-57]^+^	0.193	0.331	0.190	0.190	0.187	0.232	0.184	0.176
Ile [M-159]^+^	0.140	0.225	0.140	0.140	0.137	0.157	0.135	0.128
Glu [M-57]^+^	0.115	0.333	0.115	0.115	0.113	0.195	0.112	0.105
Pro [M-159]^+^	0.184	0.818	0.181	0.819	0.176	0.819	0.175	0.820

Cells were grown aerobically at 30°C in M9 medium with 5 g L^−1^ labeled glucose (80% 1-^13^C and 20% U-^13^C) in the presence (+Pro) and absence (−Pro) of 5 mM unlabeled proline. wt_pET, *E. coli* BL21(DE3)(pLysS)(pET-24a); Δ*putA*_pET, *E. coli* BL21Δ*putA*(DE3)(pLysS)(pET-24a); wt_p4h1of, *E. coli* BL21(DE3)(pLysS)(pET_p4h1of); Δ*putA*_p4h1of, *E. coli* BL21Δ*putA*(DE3)(pLysS)(pET_p4h1of).

The largest differences among strains were observed in the reactions mediated by the malic enzymes and the phosphoenolpyruvate carboxykinase, the TCA cycle, and the fluxes related to proline metabolism and hydroxylation. As the Δ*putA*_pET strain was not able to catabolize proline, proline addition did not have an impact on the flux distribution, which resembled that of the wt_pET strain grown on glucose only. However, the Δ*putA*_pET strain virtually substituted endogenous proline biosynthesis with the uptake of extracellular proline. In contrast, proline addition increased the acetate secretion rate by almost 25%, in the wt_pET strain, reversed the flux between *α*-KG and glutamate towards *α*-KG synthesis, increased the TCA flux from *α*-KG to succinate by almost 40%, and activated the conversion of malate to pyruvate. In all other cases, independently of proline addition, this gluconeogenetic reaction was not active. The oxidative decarboxylation of malate catalyzed by the malic enzymes ScfA and/or MaeB [19] has previously been considered absent in *E. coli* cells growing on glucose [20]. Such a flux through malic enzymes, for which simulations gave evidence only in the case of the wt_pET strain grown on glucose and proline, was not identified in our earlier study, where the pools of malate and oxaloacetate in the TCA cycle and the pools of pyruvate and phosphoenolpyruvate in glycolysis were lumped [11]. Accordingly, proline addition was suggested to reduce the anaplerotic fluxes. In order to assess whether the malate decarboxylation indeed takes place in vivo, a tracer experiment using 100% U-^13^C labeled glucose was performed and the fraction of unlabeled alanine derived from proline via malate and pyruvate was quantified. When proline was added, the unlabeled alanine *m*0 fraction of the 260, 232, and 158 [*m*/*z*] fragment ions doubled from 1.8, 1.8, and 2.2% to 3.8, 3.4, and 4.0%, respectively (see Additional file 1: Table S4). This experimentally determined increase, reaching on average approximately 1.8%, is in agreement with the 2.1% calculated theoretically from the metabolic fluxes presented in Fig. 2, confirming active flux via malic enzymes. The role of malic enzymes is not clear during growth on glucose, since the combined activities of pyruvate carboxylase, malate dehydrogenase, and malic enzymes result in net ATP consumption and can therefore be regarded as parts of a futile cycle. However, it is generally accepted that the flux from malate to pyruvate produces NADPH and thus functions as NADPH generator for biosynthetic purposes when *E. coli* grows on substrates that do not make use of glycolysis to enter central metabolism (e.g., acetate, C4-dicarboxylic acids, amino acids) [19, 21, 22]. Moreover, as phosphoenolpyruvate carboxykinase and the malic enzyme(s) may be responsible for the withdrawal of C4- and C5-intermediates from the TCA cycle, they might fulfil a cataplerotic function [23]. Thus, the addition of proline to the wt_pET strain, accompanied by the increased NADH generating flux from *a*-KG towards succinate and malate, may have led to malic enzyme activation, pyruvate surplus, and finally a higher acetate excretion rate.

In the absence of proline, *p4h1* expression and the resulting proline hydroxylation lead to a doubling of the proline synthesis rate in both strains. Additionally, recombinant P4H production was associated with a metabolic burden as reflected by higher relative TCA fluxes in both wt_p4h1of and Δ*putA*_p4h1of indicating increased biosynthetic and energy demands. When proline was added to the medium, the same effect was observed for wt_p4h1of, but not for Δ*putA*_p4h1of which retained similar relative TCA cycle fluxes as Δ*putA*_pET. Another difference between wt_p4h1of and Δ*putA*_p4h1of is the anaplerotic net flux between phosphoenolpyruvate and oxaloacetate. Upon proline hydroxylation, this flux decreased by 30% in wt_p4h1of, implying an anaplerotic role of proline metabolism, whereas it remained similar in Δ*putA*_p4h1of, as can be expected from the inability of this strain to metabolize proline. However, in wt_pET such an anaplerotic role of proline metabolism was not observed. Instead, the flux through the malic enzymes was activated as described above. For wt_p4h1of, the missing evidence for an activated malic enzyme flux can be explained by a decreased rate of proline metabolism caused by proline hydroxylation and the lower proline uptake rate.

**Fig. 3:**
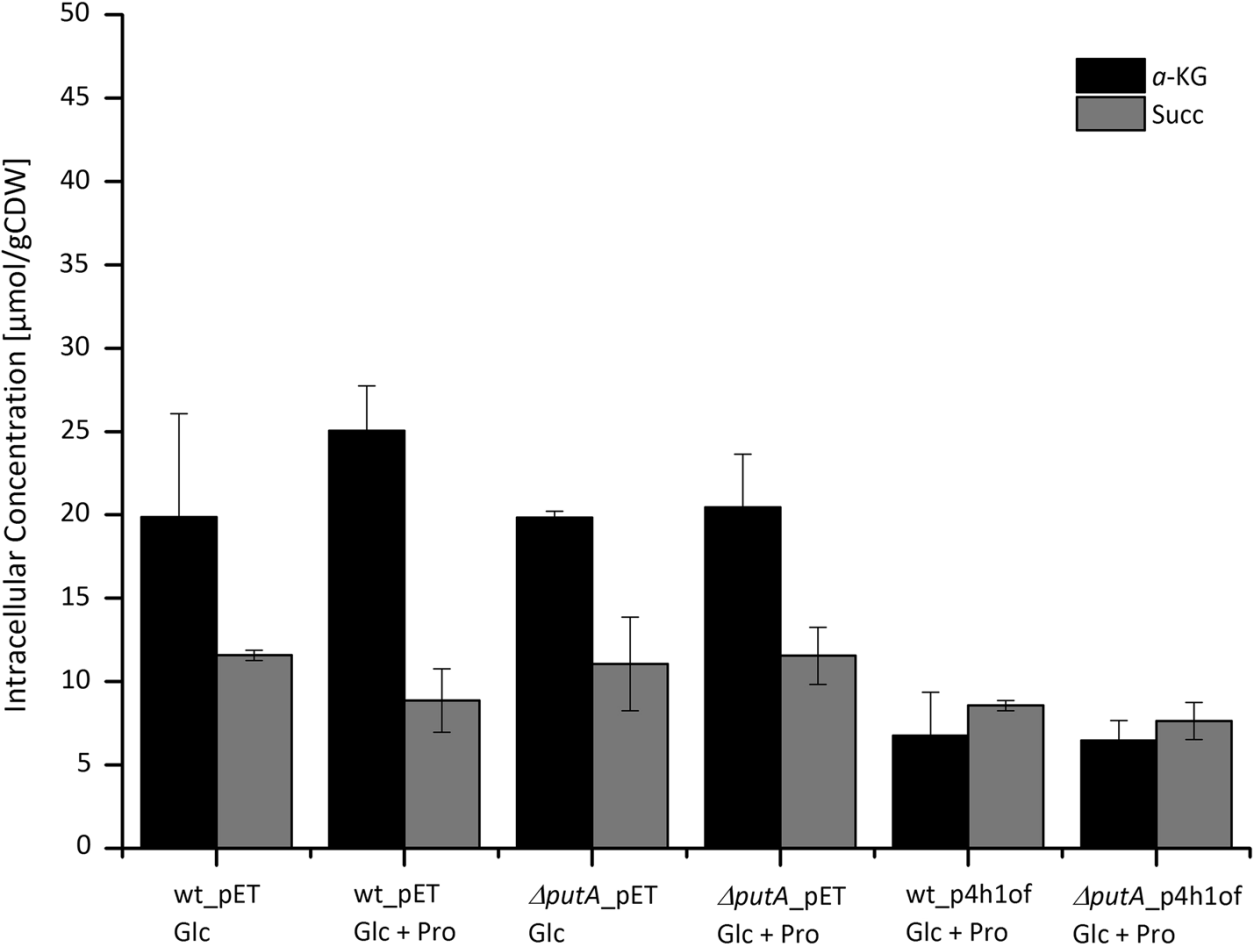
Intracellular *a*-ketoglutarate (*a*-KG) and succinate (Succ) concentrations in the mid-exponential growth phase for recombinant *E. coli* BL21(DE3)(pLysS) (wt) and *E. coli* BL21Δ*putA*(DE3)(pLysS) (Δ*putA*) containing pET-24a (pET) or pET_p4h1of (p4h1of) in M9 medium with 5 g L^−1^ glucose (Glc) in the absence or presence of 5 mM proline (Pro) at 30°C. Concentrations are given as average values in µmoles per gram cell dry weight from three different samples with standard deviations displayed as *error bars*.

In the presence of proline, the flux from *α*-KG to succinate was decreased by 60% in Δ*putA*_p4h1of compared to only 10% in wt_p4h1of, which can be ascribed to *α*-KG withdrawal for hyp synthesis. Strikingly, even though *putA* deletion led to increased proline uptake and hydroxylation rates, it did not induce a “driven-by-demand” increase of the *α*-KG generating TCA flux, pointing towards a possible limitation of P4H catalysis by the cosubstrate *α*-KG. Moreover, the intracellular *α*-KG concentrations determined for both strains (Fig. 3) show that even though the hyp formation rate is almost twice as high when using the Δ*putA*_p4h1of as compared to wt_p4h1of, P4H has to withdraw *α*-KG from a pool of similar size competing for *α*-KG with *α*-KG dehydrogenase and glutamate dehydrogenase. These results indicate that the main limitation of proline hydroxylation is shifted from intracellular proline to *α*-KG availability and thus to reactions involved in *α*-KG formation, such as oxidative isocitrate decarboxylation, rendering them promising targets for future metabolic engineering efforts.

### Decreased maintenance energy demands upon hyp synthesis

Even though stoichiometric modelling provides a precious basis to elucidate how cellular metabolism functions and responds to perturbations, it does not directly offer a deeper insight in the way bacterial cells balance catabolic energy generation with anabolic demands, including redox pools. Thus, based on the metabolic fluxes derived from ^13^C-MFA, net formation rates of NTPs (ATP and GTP), NAD(P)H, and FADH_2_ were determined (Table 3, See also Additional file 1: Table S5). The rate of NTP formation via substrate-level phosphorylation was calculated based on glycolysis, TCA cycle, and acetic acid formation rates. The ATP generating pathway from acetyl-CoA to acetate catalyzed by phosphotransacetylase and acetate kinase was considered, assuming that the flux through pyruvate oxidase B (PoxB) plays a minor role [24]. The growth-related demand for energy and redox equivalents was calculated using a stoichiometric equation for biomass formation generated by the FiatFlux software (see Additional file 2) [25–27]. The equation contains the specific growth rate (µ) as a variable, thereby considering the differing macromolecular biomass composition at different growth rates [28]. The rate of ATP formation via oxidative phosphorylation was calculated based on the assumptions that (1) the NADH and FADH_2_ not used for biomass formation are utilized for ATP formation at the maximum P/O ratio (NADH → 3ATP, FADH_2_ → 2ATP) [29], and (2) only NADH and not NADPH is oxidized by the respiratory chain [30].

**Table 3:** Effect of *putA* deletion, *p4h1of* expression, proline addition, and proline hydroxylation on NTP and cofactor formation and consumption rates

	wt_pET		Δ*putA_*pET		wt_p4h1of		Δ*putA*_p4h1of	
	−Pro	+Pro	−Pro	+Pro	−Pro	+Pro	−Pro	+Pro
Catabolism^a^								
NADPH	6.4	8.0	6.8	6.8	6.6	6.2	6.4	4.7
NADH	17.8	19.1	18.5	17.7	19.2	17.6	19.3	12.9
FADH_2_	2.1	3.7	2.5	2.5	3.0	2.9	3.1	1.7
ATP/GTP	14.1	14.5	14.3	13.4	14.1	13.3	14.3	10.1
Biomass formation								
NADPH	−7.3	−7.9	−7.0	−7.3	−5.7	−5.7	−5.2	−5.4
NADH	1.2	1.3	1.1	1.2	0.9	0.9	0.8	0.8
FADH_2_	–	–	–	–	–	–	–	–
ATP	−7.3	−8.1	−7	−7.3	−5.4	−5.4	−4.9	−5.1
Transhydrogenation								
NADPH	0.9	−0.1	0.2	0.5	−0.9	−0.5	−1.2	0.7
NADH	−0.9	0.1	−0.2	−0.5	0.9	0.5	1.2	−0.7
Oxidative phosphorylation								
NADPH	–	–	–	–	–	–	–	–
NADH	−18.1	−20.5	−19.4	−18.4	−21.0	−19.0	−21.3	−13.0
FADH_2_	−2.1	−3.7	−2.5	−2.5	−3.0	−2.9	−3.1	−1.7
ATP	58.4	67.3	63.3	60.1	68.9	62.3	70.0	42.5
Maintenance^b^								
ATP	−65.2	−73.7	−70.6	−66.2	−77.6	−70.2	−79.4	−47.5

*E. coli* BL21(DE3)(pLysS) strains were grown in M9 medium containing 5 g L^−1^ glucose and optionally 5 mM proline (Pro) at 30°C and 250 rpm. Rates are given in mmol g_CDW_^−1^ h^−1^. wt_pET,  *E. coli* BL21(DE3)(pLysS)(pET-24a); Δ*putA*_pET, *E. coli* BL21Δ*putA*(DE3)(pLysS)(pET-24a); wt_p4h1of, *E. coli* BL21(DE3)(pLysS)(pET_p4h1of); Δ*putA*_p4h1of, *E. coli *BL21Δ*putA*(DE3)(pLysS)(pET_p4h1of)

^a^The calculations were based on the reactions described in Additional file 1: Table S5 and included in the metabolic map presented in Fig. 2.

^b^The ATP costs for maintenance were not divided into growth and non-growth associated maintenance.

Substrate-level phosphorylation occurred at similar rates with all strains and under all conditions tested except for Δ*putA*_p4h1of which displayed a significantly lower rate in the presence of proline (Table 3). This low NTP formation rate can be explained by the decreased flux from *α*-KG to succinate upon P4H catalysis at high rates. The NTP consumed by the pET containing strains for cell growth was at similar levels, except for wt_pET in the presence of proline which showed a higher NTP demand due to increased biomass formation resulting from proline assimilation.

Upon P4H synthesis, the NTP amounts invested for cell growth on glucose decreased by 26 and 30% for the wt_p4h1of and Δ*putA*_p4h1of, respectively. The same phenomenon was observed when proline was added to the medium, i.e., 33 and 30% decrease of cell growth-associated NTP consumption, respectively. The metabolic burden of P4H production imposed on the cells when grown on glucose as sole source of carbon was also manifested by an increased respiratory activity that led to 18 and 10% higher NTP formation rates in wt_p4h1of and Δ*putA*_p4h1of, respectively, and this at a lower growth rate [31, 32]. Accordingly, enhanced maintenance requirements were observed during recombinant protein production as a non-growth associated function that is in agreement with other published results [32, 33]. This situation changed when proline was added to the medium. In Δ*putA*_p4h1of, the NADH generating flux from *α*-KG to succinate was partially replaced by the biotransformation and, in wt_p4h1of, less proline was converted to glutamate, both resulting in decreased NADH/FADH_2_ formation and thus reduced oxidative phosphorylation. This effect was more prominent for Δ*putA*_p4h1of.

ATP produced and not consumed for growth-related production of cellular material typically is consumed for maintenance processes such as maintenance of electrochemical gradients across the plasma membrane that can reach up to 50% of the ATP produced, degradation and regeneration of cellular macromolecules, futile cycles, energy spilling reactions, proofreading, and cell motility [34, 35]. Based on the calculations performed for Δ*putA*_p4h1of, the most noteworthy bioenergetic alteration caused by P4H activity consists in the dramatic decrease of NADH available for oxidative phosphorylation. Most interestingly, it seems that, upon hyp synthesis, the engineered strain responded by optimizing its energetic efficiency by reducing its maintenance energy demand instead of increasing the TCA activity. This is especially remarkable as recombinant *p4h1of* expression itself had the opposite effect increasing the energy demand. Taking into account that the stoichiometry of energy transducing membranes is not fixed and that the maximum P/O ratios assumed in Table 3 will not be reached in reality, the engineered strain may possibly utilize glucose more efficiently by exhibiting a higher in vivo P/O ratio.

## Conclusions

In the current study, a *putA* knockout mutant of *E. coli* BL21 overproducing *a*-KG-dependent proline-4-hydroxylase (P4H) was generated and physiologically analyzed to uncover the interference of proline hydroxylation with host cell physiology and metabolism. The deletion of the *putA* gene has already been reported to be a successful engineering strategy for efficient *trans*-4-hydroxy-l-proline production [10]. Here, a detailed insight into the metabolic operation of engineered *E. coli* BL21 upon recombinant P4H catalysis is provided. The results show that the mutant strain unable to catabolize proline displayed not only a higher yield of hyp on proline, but also a higher specific hyp formation rate and a higher hyp yield on glucose as compared to the wildtype. Metabolic flux analysis revealed that, although the hyp formation rate increased when using the mutant strain, the TCA flux supplying *a*-KG (isocitrate to *a*-KG) did not increase upon accelerated P4H catalysis, leading to improved glucose utilization efficiency and pointing to a possible limitation in *a*-KG. Furthermore, a redox and energy balance study based on the ^13^C-MFA allowed a quantitative assessment of how the cells coordinate catabolism with anabolism during product synthesis. It was shown that P4H catalyzed proline hydroxylation in the mutant strain decreases TCA-mediated energy formation by 25% compared to the wildtype. Obviously, the engineered strain compensated this ATP loss by reducing its maintenance energy demand, e.g., by improving the P/O ratio for more efficient glucose utilization, emphasizing the role of maintenance and respiratory chain operation in dynamic cellular adaptation strategies.

## Methods

### Chemicals

1-^13^C (99%) and U-^13^C labeled glucose (99%) was purchased either from Sigma-Aldrich (Munich, Germany) or Cambridge Isotope Laboratories (Andover, MA, USA); l-proline was kindly provided by Evonik Rexim SAS (Ham, France); all other chemicals were purchased from Sigma-Aldrich (Munich, Germany) or Carl-Roth (Karlsruhe, Germany) and were of the highest purity available.

### Bacterial strains, plasmids, and cultivation conditions

The strains and plasmids used in this study are listed in Additional file 1: Table S6. For the *putA* gene deletion, the Quick & Easy *E.coli* Gene Deletion Kit based on Red^®^/ET^®^ Recombination (Gene Bridges GmbH, Heidelberg, Germany) was used. Recombinant *E. coli* strains were routinely precultured in LB complex medium [36] followed by precultivation and cultivation in M9 mineral medium composed of 8.5 g L^−1^ Na_2_HPO_4_·2H_2_O, 3.0 g L^−1^ KH_2_PO_4_, 0.5 g L^−1^ NaCl, 1.0 g L^−1^ NH_4_Cl, and 2 mL L^−1^ 1 M MgSO_4_, which contained 10 mg L^−1^ thiamine, 5 mg L^−1^ biotin, 1 mL L^−1^ US^Fe^ trace element solution [37], 5 g L^−1^ glucose, 5 mM proline (when specified), 34 mg L^−1^ chloramphenicol, and 50 mg L^−1^ kanamycin. Such M9 medium was used for main cultures using non-labeled glucose to investigate the physiology and ^13^C-labeled glucose to perform tracer experiments. For the induction of *p4h1of* expression, 1 mM isopropyl-*β*-D-thiogalactopyranoside (IPTG) was added to both the M9 preculture and the main culture at inoculation. All strains were incubated in baffled Erlenmeyer flasks in horizontal shakers at 30°C and 250 rpm.

### Analytical procedures

Bacterial cell growth was monitored by measuring the optical density at 600 nm (OD_600_), using a Libra S11 spectrophotometer (Biochrom Ltd., Cambridge, UK). The correlation factors between OD_600_ and cell dry weight (CDW) concentration under the conditions investigated were determined as described elsewhere [38] and are listed in the Additional file 1: Table S7. For the determination of extracellular consumption and secretion rates, samples taken during exponential growth were centrifuged for 10 min at 4°C and 13,000 g. Glucose and acetate were quantified in the resulting supernatants using a LaChrom Elite^®^ HPLC System (VWR International GmbH, Darmstadt, Germany) equipped with a Trentec 308R-Gel.H ion exclusion column (300 × 8 mm, Trentec Analysentechnik, Gerlingen, Germany) at 40°C with 5 mM H_2_SO_4_ as mobile phase at a flow rate of 1 mL min^−1^. Analytes were detected either by a UV (λ = 210 nm) (VWR Hitachi L-2420) or refractive index (RI) detector (VWR Hitachi L-2490). Proline and hyp were quantified using a LaChrom Elite^®^ HPLC System (VWR International GmbH, Darmstadt, Germany) equipped with a diode array detector (VWR Hitachi L-2450) at 200 nm and an Intersil ODS-3 column (GL Sciences B.V., Eindhoven, Netherlands). The separation was performed at 22°C and 1 mL min^−1^ flow rate applying a linear gradient profile based on 20 mM KH_2_PO_4_ buffer (pH 2.3) containing 1% (v/v) acetonitrile as eluent A and acetonitrile containing 10% (v/v) water as eluent B as follows: 85% eluent A for 2 min, 85% to 70% eluent A within 4 min, 70% eluent A for 8 min, and 70–85% eluent A within 0.5 min. Before analysis, the analytes were derivatized using benzoyl chloride. Briefly, supernatant was vortex-mixed with 100 mM benzoyl chloride in acetonitrile and 0.5 M K_2_HPO_4_ (pH 11.7) in a 1:2.5:9 (v/v) ratio followed by incubation for 10 min at room temperature and 20 min at 50°C with stirring. Before analysis, derivatized samples were diluted in a 4:1 ratio using eluent A and 50% phosphoric acid in a 7.5:1 (v/v) ratio. Alternatively, a spectrophotometric method described elsewhere [11] was used for hyp quantification. SDS-PAGE was performed to monitor recombinant protein production [39] using an AlphaImager HP documentation system (Biozym, Hessisch Oldendorf, Germany) for gel imaging.

The quantification of intracellular metabolites was based on a modified fast filtration method described by Bolten et al. [40]. Briefly, cells were separated from the medium via vacuum filtration using a water jet pump and cellulose nitrate filter membranes (Whatman, pore size: 0.2 µm). Two milliliters of cell culture were transferred onto the filter membrane and washed twice with 5 mL of washing solution (NaCl solution with an osmolarity equivalent to the M9 medium). The filter was put upside-down into a 100 mL Schott flask that contained 2 mL of ice-cold extraction solution (45:45:1:9 of acetonitrile, 2,2,2-trifluoroethanol, trifluoroacetic acid, and water [41]) and incubated for 20 min. The quenching was performed in less than 1 min. The extraction solution was then transferred to 2 mL Eppendorf tubes and centrifuged for 5 min at 4°C and 13,300 rpm. The supernatant was then derivatized for GC–MS analysis in two-steps, first oximation using an aqueous methoxylamine solution and then silylation using MBDSTF [42].

### ^13^C-labeling experiments

*E. coli* strains (wildtype and Δ*putA*) were precultured aerobically in 5 mL M9 minimal medium supplemented with 5 g L^−1^ of glucose at 30°C and 250 rpm. In the mid-exponential growth phase, cells were harvested by centrifugation at 4,000*g* and 4°C for 5 min, washed once with M9 medium to remove unlabeled glucose, and used to inoculate M9 medium containing 5 g L^−1^ of a 4:1 mixture of 1-^13^C-labeled and U-^13^C labeled glucose to an OD_600_ of 0.05 or lower to minimize introduction of non-labeled carbon from precultivation. For labeling analysis of proteinogenic amino acids, cells were harvested in the mid-exponential growth phase (at an OD_600_ of 2–2.5) by centrifugation for 10 min at 13,000*g* and 4°C, and washed twice by resuspension in 1 mL 0.9% (w/v) NaCl. The washed pellet was resuspended in 150 μL of 6 M HCl and hydrolyzed for 6 h at 105°C in a well-sealed vial to prevent evaporation. After drying overnight at 85°C, the hydrolyzate was resuspended in 30 µL acetonitrile, and amino acid silylation was initiated by the addition of 30 µl *N*-methyl-*N*-tert-butyldimethylsilyl-trifluoracetamide (MBDSTFA) followed by incubation at 85°C for 60 min. The quantification of mass isotopomer distributions (MIDs) of 12 amino acids (alanine, glycine, valine, leucine, isoleucine, proline, serine, threonine, phenylalanine, aspartate, glutamate, tyrosine) from biomass hydrolyzates was performed as previously described [43] using a GC 3800 combined with a MS/MS 1,200 unit (Varian Deutschland GmbH, Darmstadt, Germany).

### Metabolic modeling and calculation of metabolic fluxes

The metabolic network of *E. coli* was adapted from literature [20, 44] and comprised glycolysis (EMP), pentose phosphate pathway (PPP), tricarboxylic acid (TCA) cycle with the glyoxylate bypass, anaplerotic carboxylation and decarboxylation reactions, acetate formation, proline hydroxylation, and the reactions among proline, glutamate, and *α*-ketoglutarate (*α*-KG). The stoichiometric model contained 58 reactions and 38 metabolites in case of growth on glucose only, and 65 reactions and 40 metabolites in case proline was added to the medium. The model was constrained with (1) 4 extracellular fluxes (glucose and proline uptake and acetate and hyp formation), (2) the anabolic precursor requirements for biomass formation, as previously described [44], corrected for the experimentally determined biomass yield on glucose, and (3) the ^13^C-isotopomer labeling pattern of proteinogenic amino acids. Given that the glyoxylate bypass is considered to be inactive in *E. coli* grown on glucose and active when grown on acetate [24], the pathway was set to zero. Quantitative analysis of the intracellular metabolic fluxes was performed with OpenFlux [45] and all numerical calculations with Matlab 7.9 (The Mathworks Inc., Natick, MA, USA). The best-fit values of the intracellular carbon fluxes were estimated by minimizing the deviation between experimentally determined and simulated MIDs. Standard deviations were estimated using the Monte Carlo approach included in OpenFLUX and expressed as upper and lower boundaries for intervals of 95% confidence [46]. Stoichiometries and related files are presented in the Additional file 3.

Rates for in vivo energy production in the form of NTP and redox cofactors NAD(P)H and FADH_2_ were estimated from the catabolic reactions using the optimal set of fluxes obtained from ^13^C-MFA. Growth-related NADPH and ATP consumption rates were estimated using a lumped stoichiometric equation for biomass formation derived from the FiatFlux software [26], in which the growth rate is integrated considering that macromolecular biomass composition is growth rate dependent [47] (see Additional file 2). Maximal ATP formation rates via electron transport chain and oxidative phosphorylation were estimated using the maximum theoretical P/O ratios of 3 and 2 for NADH and FADH_2_, respectively [29]. Maximal NAD(P)H and ATP demands for maintenance were calculated by subtracting the growth-related consumption rate from the production rate.

## Additional files

Additional file 1:
**Figure S1.** Physiology of recombinant *E. coli* BL21(DE3)(pLysS) strains bearing pET-24a. Panel A and B show biomass formation (circles), glucose consumption (squares), acetate formation (triangles), and proline consumption (diamonds) during batch cultivation of wildtype (closed symbols) and *ΔputA* (open symbols) strains at 30°C in M9 medium supplemented with 5 g L^−1^ glucose in the absence (A) or presence (B) of 5 mM proline, respectively. **Figure S2.** SDS-PAGE analysis of recombinant *E. coli* BL21(DE3)(pLysS) (pET_p4h1of) and *E. coli* BL21 *ΔputA* (DE3) (pLysS) (pET_p4h1of) at different time points during growth in M9 medium with 5 g L^−1^ glucose (glc) only or with addition of 5 mM proline (pro) at 30°C. M: protein size marker. **Figure S3.** Physiology of recombinant *E. coli* BL21(DE3)(pLysS) strains bearing pET_p4h1of. Biomass formation (circles), glucose consumption (squares), acetate formation (triangles), hyp formation (stars), and proline consumption (diamonds) during batch cultivation of wildtype (closed symbols) and *ΔputA* (open symbols) strains are shown. Cultivation was performed at 30°C in M9 medium supplemented with 5 g L^−1^ glucose in the absence (panel A) or presence of 5 mM proline (panels B and C). **Table S4.** Mass isotopomer distribution of alanine for the wt_pET strain at 30°C in M9 medium supplemented with 5 g L^−1^ U-^13^C labeled glucose in the absence or presence of 5 mM proline. **Table S5.** Reactions of the central carbon metabolism generating or consuming NTP and/or redox equivalents. **Table S6.** Bacterial strains and plasmids used in this study. **Table S7.** Correlation factors between OD600 1 and cell dry weight concentration (g_CDW_ L^−1^) of the strains used in this study. Additional file 2. Anabolic demands for biomass formation as a function of the growth rate. Additional file 3. Supplementary data for ^13^C-MFA including reactions considered in the model, comparisons between experimental and simulated mass distribution vectors (MDVs), Monte Carlo analysis, and mass isotopomer distributions of the amino acids.

## Abbreviations

*α*-KG: *α*-ketoglutarate
ATP: adenosine triphosphate
CDW: cell dry weight (g L^−1^)
^13^C-MFA: ^13^C-metabolic flux analysis
Cp: chloramphenicol
EMP: Embden–Meyerhof–Parnas
FAD: flavin adenine dinucleotide
HPLC: high performance liquid chromatography
Glc: glucose
hyp: *trans*-4-l-hydroxy-proline
IPTG: isopropyl β-d-thiogalactoside
Km: kanamycin
λ: wavelength (nm)
LB: lysogeny broth
NAD(P): nicotinamide adenine dinucleotide (phosphate)
MBDSTFA: *N*-methyl-*N*-tert-butyldimethylsilyl-trifluoracetamide
MID: mass isotopomer distribution
OD_600_: optical density at 600 nm
P/O: phosphate/oxygen
PPP: pentose phosphate pathway
pro: proline
P4H: proline-4-hydroxylase
putA: proline utilization protein A
putP: Na^+^/proline symport carrier
RID: refractive index detector
SDS-PAGE: sodium dodecyl sulfate polyacrylamide gel electrophoresis
TCA: tricaboxylic acid
UV/VIS: ultraviolet/visible
wt: wild type
μ: exponential growth rate (h^−1^)
Δ*putA*: knockout of *putA* gene encoding proline dehydrogenase (PutA)
