# Supplementary material for: Metabolic network capacity of Escherichia coli for Krebs cycle-dependent proline hydroxylation
Source: Microb Cell Fact. 2015 Jul 29;14:108. doi: 10.1186/s12934-015-0298-1 (PMC4517350; doi:10.1186/s12934-015-0298-1)
Supplement: Additional file 1: — Figure S1. Physiology of recombinant E. coli BL21(DE3)(pLysS) strains bearing pET-24a. Panel A and B show biomass formation (circles), glucose consumption (squares), acetate formation (triangles), and proline consumption (diamonds) during batch cultivation of wildtype (closed symbols) and ΔputA (open symbols) strains at 30°C in M9 medium supplemented with 5 g L−1 glucose in the absence (A) or presence (B) of 5 mM proline, respectively. Figure S2. SDS-PAGE analysis of recombinant E. coli BL21(DE3)(pLysS) (pET_p4h1of) and E. coli BL21 ΔputA (DE3) (pLysS) (pET_p4h1of) at different time points during growth in M9 medium with 5 g L−1 glucose (glc) only or with addition of 5 mM proline (pro) at 30°C. M: protein size marker. Figure S3. Physiology of recombinant E. coli BL21(DE3)(pLysS) strains bearing pET_p4h1of. Biomass formation (circles), glucose consumption (squares), acetate formation (triangles), hyp formation (stars), and proline consumption (diamonds) during batch cultivation of wildtype (closed symbols) and ΔputA (open symbols) strains are shown. Cultivation was performed at 30°C in M9 medium supplemented with 5 g L−1 glucose in the absence (panel A) or presence of 5 mM proline (panels B and C). Table S4. Mass isotopomer distribution of alanine for the wt_pET strain at 30°C in M9 medium supplemented with 5 g L−1 U-13C labeled glucose in the absence or presence of 5 mM proline. Table S5. Reactions of the central carbon metabolism generating or consuming NTP and/or redox equivalents. Table S6. Bacterial strains and plasmids used in this study. Table S7. Correlation factors between OD600 1 and cell dry weight concentration (gCDW L−1) of the strains used in this study. [file 12934_2015_298_MOESM1_ESM.docx]

**
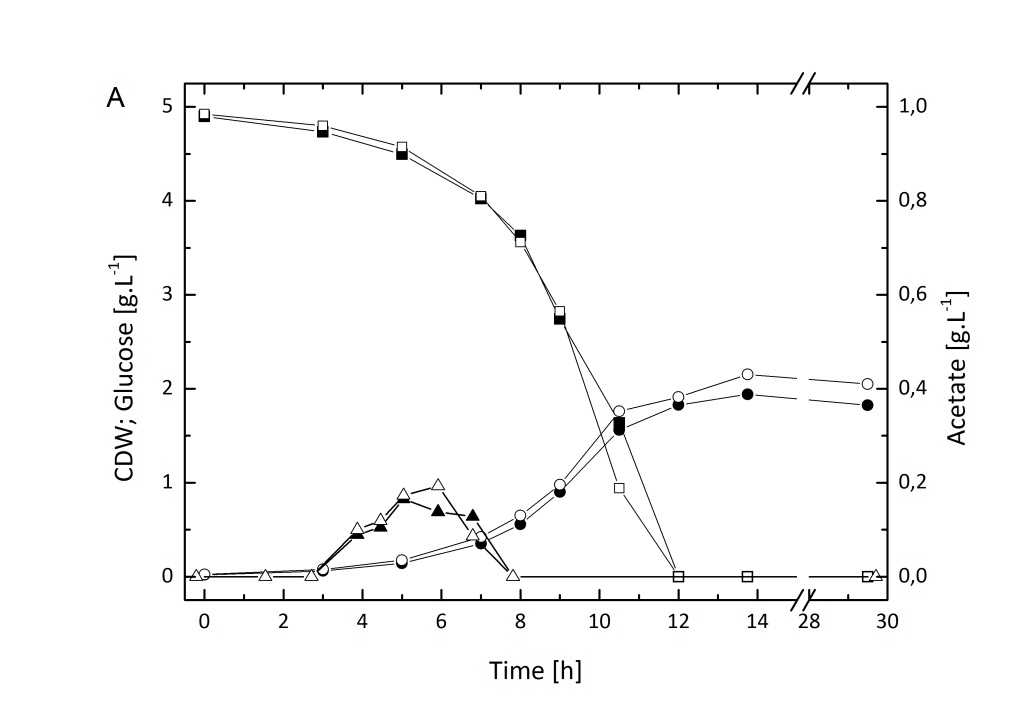

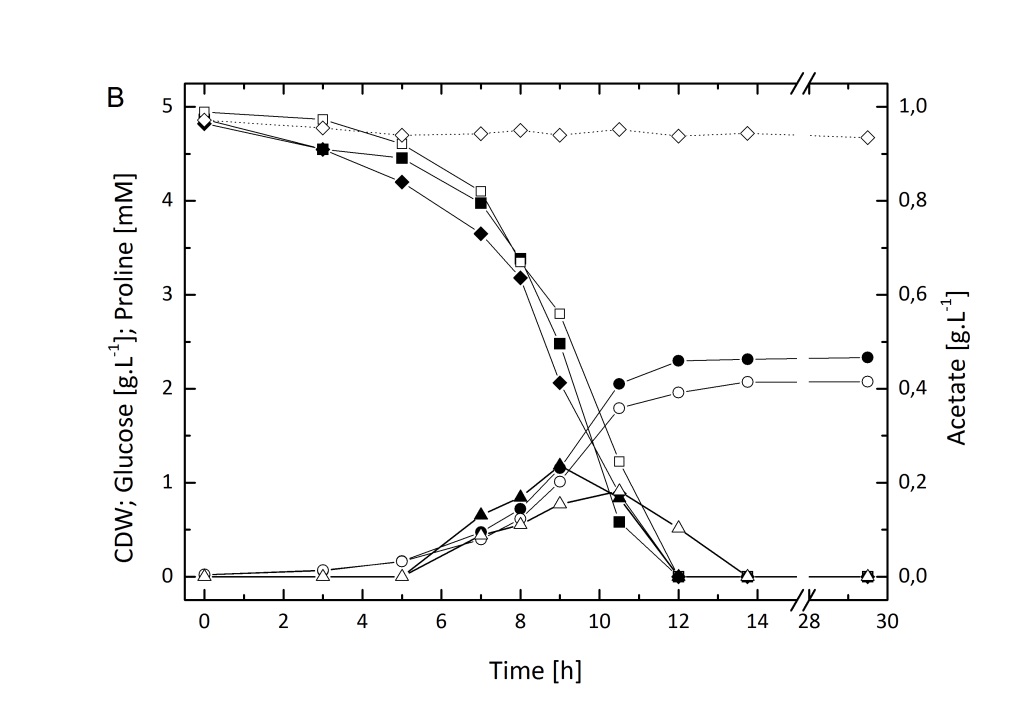
**

**Figure S1 Physiology of recombinant *E. coli* BL21(DE3)(pLysS) strains bearing pET-24a.** Panel A and B show biomass formation (circles), glucose consumption (squares), acetate formation (triangles), and proline consumption (diamonds) during batch cultivation of wildtype (closed symbols) and *ΔputA* (open symbols) strains at 30 °C in M9 medium supplemented with 5 g L^-1^ glucose in the absence (A) or presence (B) of 5 mM proline, respectively.

**
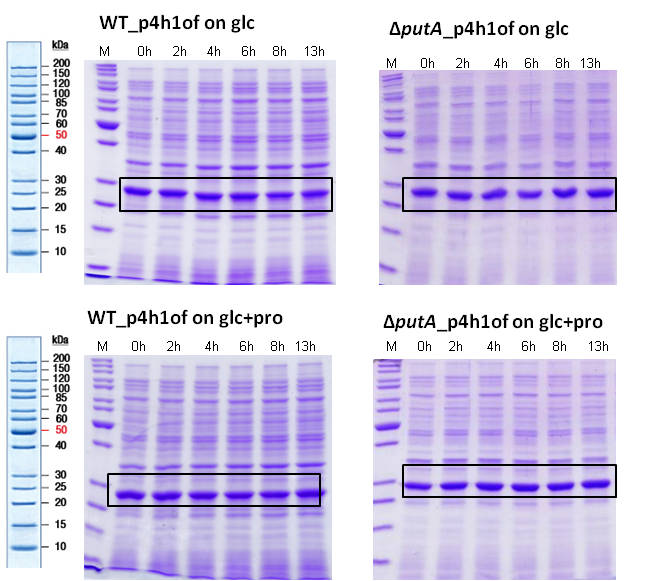
**

**Figure S2 SDS-PAGE of whole cells of recombinant *E. coli* BL21(DE3)(pLysS) (pET_p4h1of) and *E. coli* BL21 *ΔputA* (DE3) (pLysS) (pET_p4h1of)** at different time points during growth in M9 medium with 5 g L^-1^ glucose (glc) only or with addition of 5 mM proline (pro) at 30 °C. M: protein size marker.

**
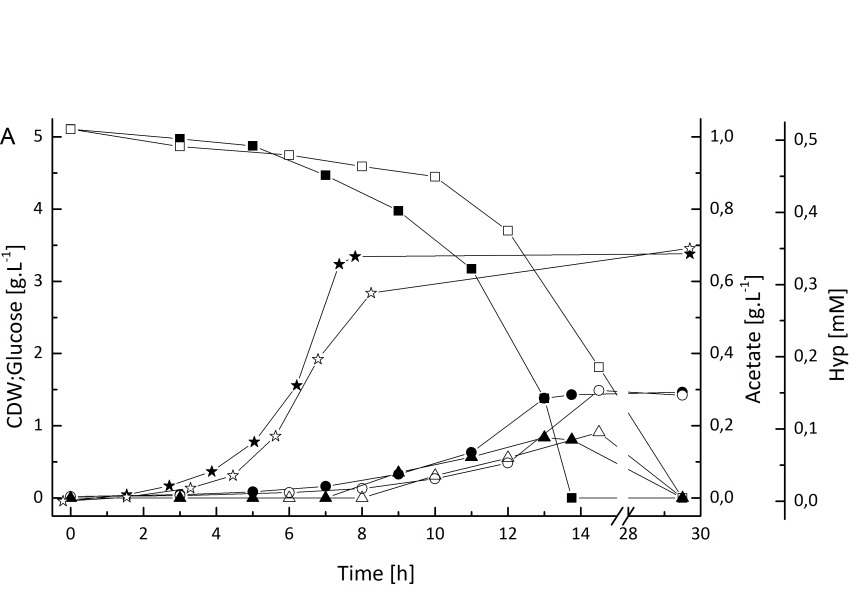

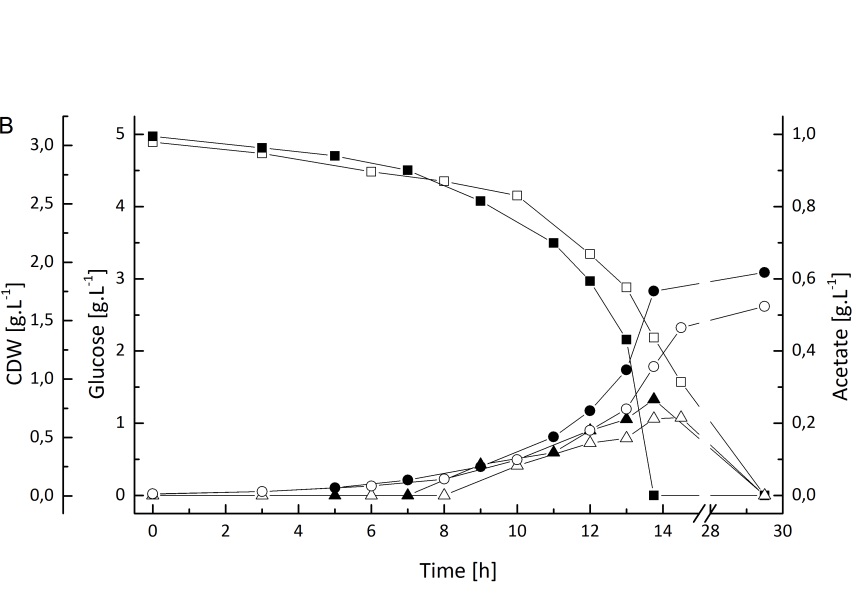
**

**
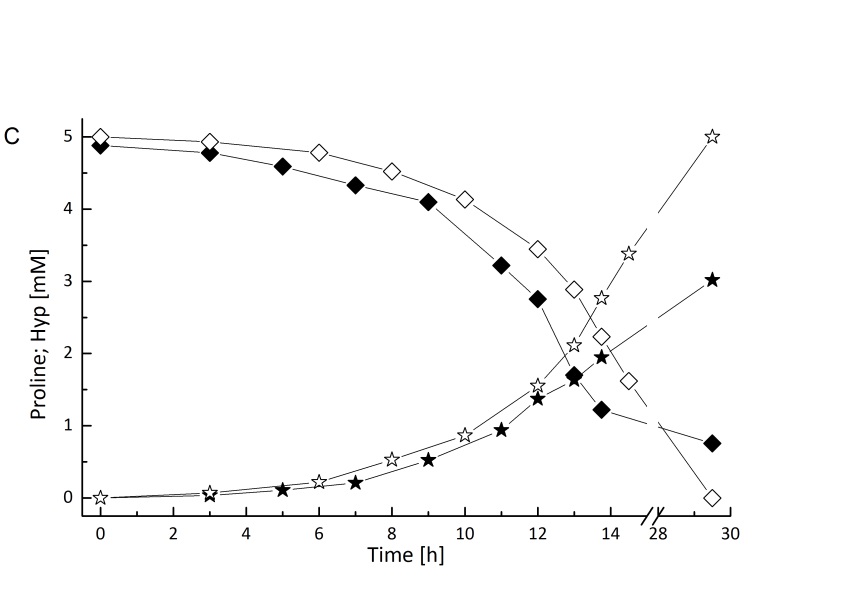
**

**Figure S3 Physiology of recombinant *E. coli* BL21(DE3)(pLysS) strains bearing pET_p4h1of.** Biomass formation (circles), glucose consumption (squares), acetate formation (triangles), hyp formation (stars), and proline consumption (diamonds) during batch cultivation of wildtype (closed symbols) and *ΔputA* (open symbols) strains are shown. Cultivation was performed at 30 °C in M9 medium supplemented with 5 g L^-1^ glucose in the absence (panel A) or presence of 5 mM proline (panels B and C).

**Table S4 Mass isotopomer distribution of alanine for the wt_pET strain at 30 °C in M9 medium supplemented with 5 g L^-1^ U-^13^C labeled glucose in the absence or presence of 5 mM proline.**

|  |  |  |  |  |  |  |  |  |  |
| --- | --- | --- | --- | --- | --- | --- | --- | --- | --- |
|  |  | **WT_pET24a** | | | | | | | |
|  |  | **Glucose** | | | | **Glucose + Proline** | | | |
|  | **Fragment** | **M0** | **M1** | **M2** | **M3** | **M0** | **M1** | **M2** | **M3** |
| Ala260 | [M-57]^+^ | 0.018±0.002 | 0.009±0.002 | 0.051±0.001 | 0.720±0.006 | 0.034±0.003 | 0.018±0.001 | 0.057±0.002 | 0.696±0.005 |
| Ala232 | [M-85]^+^ | 0.018±0.003 | 0.048±0.003 | 0.726±0.001 | 0.147±0.002 | 0.035±0.004 | 0.054±0.004 | 0.711±0.006 | 0.141±0 |
| Ala158 | [M-159]^+^ | 0.021±0.003 | 0.047±0 | 0.781±0.005 | 0.114±0.001 | 0.040±0.004 | 0.059±0 | 0.753±0.002 | 0.113±0.001 |

**Table S5 Reactions of the central carbon metabolism generating or consuming NTP and/or redox equivalents.**

|  | **Stoichiometry of biochemical reactions** | **Metabolic Pathway** |
| --- | --- | --- |
| NTP | Fructose-6-Phosphate + ATP → 2 Triose-3-phosphate | Glycolysis |
|  | Triose-3-Phosphate ↔ 3-phosphoglycerate + ATP + NADH | Glycolysis |
|  | Phosphoenolpyruvate → Pyruvate + ATP | Glycolysis |
|  | Acetyl-CoA ↔ Acetate + ATP | Acetate formation |
|  | *α*-Ketoglutarate → Succinate + CO_2_ + NADH + ATP/GTP | TCA cycle |
|  | Glutamate + ATP + 2 NADPH → Proline | Proline/Glutamate metabolism |
| NADPH | Glucose 6-Phopshpate → Pentose 5-phosphate + CO_2_ + 2 NADPH | PP pathway |
|  | Isocitrate ↔ *α*-ketoglutarate + CO_2_ + NADPH | TCA cycle |
|  | Malate → pyruvate + CO_2_ + NAD(P)H | Gluconeogenesis |
|  | Glutamate ↔ NH_3_ + *α*-ketoglutarate + NADPH | Proline/Glutamate metabolism |
|  | Glutamate + ATP + 2 NADPH → Proline | Proline/Glutamate metabolism |
| NADH | Triose-3-P ↔ 3-phosphoglycerate + ATP + NADH | Glycolysis |
|  | Pyruvate → Acetyl-coA + NADH + CO_2_ | Glycolysis |
|  | α-Ketoglutarate → Succinate + CO_2_ + NADH + ATP/GTP | TCA cycle |
|  | Malate ↔ Oxaloacetate + NADH | TCA cycle |
|  | Proline → Glutamate + NADH + FADH_2_ | Proline/Glutamate metabolism |
| FADH_2_ | Succinate → Fumarate + FADH_2_ | TCA cycle |

**Table S6 Bacterial strains and plasmids.**

| **Strain or Plasmid** | **Description** | **Source** |
| --- | --- | --- |
| BL21(DE3) (pLysS) | F^–^*ompT hsdS_B_*(r_B_^–^ m_B_^–^) *gal dcm* (DE3) pLysS (Cm^R^) | Invitrogen^TM^USA |
| pLysS | Encodes T7 lysozyme that reduces basal level expression from T7 promoter-containing plasmids when not induced, Cm^R^ | [1] |
| BL21Δ*putA*(DE3)(pLysS) | Knockout of *putA* encoding proline dehydrogenase (PutA) | This study |
| pET-24a | High copy number vector, *lacI/PT7lac*, Km^R^ | Novagen |
| pET*_*p4h1of | pET-24a containing a codon-optimized *p4h1of* gene (*Dactylosporangium* sp), Km^R^ | [2] |

**Table S7 Correlation factors between OD_600_ 1 and cell dry weight concentration (g_CDW_ L^-1^) of the strains used in this study.**

| **Strain** | **Abbreviation** | **M9 with glucose** | **M9 with glucose**  **and proline** | **Reference** |
| --- | --- | --- | --- | --- |
|  |  |  |  |  |
| BL21(DE3)(pLysS)(pET-24a) | wt_pET | 0.386 | 0.390 | [2] |
| BL21(DE3)(pLysS)(pET_*p4h1of*) | wt_p4h1of | 0.339 | 0.337 | [2] |
| BL21Δ*putA*(DE3)(pLysS)(pET-24a) | Δ*putA_*pET | 0.423 | 0.423 | This study |
| BL21Δ*putA*(DE3)(pLysS)(pET_*p4h1of*) | Δ*putA_* p4h1of | 0.364 | 0.364 | This study |

**References**

1. Moffatt BA and Studier FW. T7 lysozyme inhibits transcription by T7 RNA polymerase. Cell. 1998;49(2):221.

2. Falcioni F, Blank LM, Frick O, Karau A, Bühler B, Schmid A. Proline Availability regulates proline-4-hydroxylase synthesis and substrate uptake in proline-hydroxylating recombinant *Escherichia coli*. Appl Environ Microbiol. 2013;79(9):3091.
