# Supplementary material for: Metabolic network capacity of Escherichia coli for Krebs cycle-dependent proline hydroxylation
Source: Microb Cell Fact. 2015 Jul 29;14:108. doi: 10.1186/s12934-015-0298-1 (PMC4517350; doi:10.1186/s12934-015-0298-1)
Supplement: Additional file 3. — Supplementary data for 13C-MFA including reactions considered in the model, comparisons between experimental and simulated mass distribution vectors (MDVs), Monte Carlo analysis, and mass isotopomer distributions of the amino acids. [file 12934_2015_298_MOESM3_ESM.docx]

**Supplementary data for ^13^C-MFA**

**BL21(DE3) (pLysS) (pET-24a) on glucose model**

| rxnID | rxnEQ | cTrans | rates | type | basis | deviation |
| --- | --- | --- | --- | --- | --- | --- |
| R01 | GLC_EX = GLC6P | abcdef = abcdef |  | F | 1 | 0 |
| R02 | GLC6P = F6P | abcdef = abcdef |  | FR |  |  |
| R03 | F6P=GLC6P | abcdef = abcdef |  | R | x |  |
| R04 | F6P = DHAP + G3P | abcdef = abc + def |  | F |  |  |
| R05 | DHAP = G3P | abc = cba |  | F |  |  |
| R06 | GLC6P = P5P + CO2 | abcdef = bcdef + a |  | F | X |  |
| R07 | P5P + P5P = S7P + G3P | abcde + fghij = fgabcde + hij |  | F |  |  |
| R08 | S7P + G3P = E4P + F6P | abcdefg + hij = defg + abchij |  | F |  |  |
| R09 | E4P + P5P = F6P + G3P | abcd + efghi = efabcd + ghi |  | F |  |  |
| R10 | G3P = 3PG | abc = abc |  | F |  |  |
| R11 | 3PG = PEP | abc = abc |  | FR |  |  |
| R12 | PEP = 3PG | abc = abc |  | R | X |  |
| R13 | PEP = PYR | abc = abc |  | F |  |  |
| R14 | PYR = ACCOA + CO2 | abc = bc + a |  | F |  |  |
| R15 | ACCOA + OAA = CIT | ab + cdef = fedbac |  | F |  |  |
| R16 | CIT = AKG + CO2 | abcdef = abcde + f |  | F |  |  |
| R17 | CIT = S_PREC + GLYOX | abcdef = cdef + ab |  | F | 0 |  |
| R18 | S_PREC = 0.5 SUC + 0.5 SUC | abcd = 0.5 abcd + 0.5 dcba |  | F |  |  |
| R19 | GLYOX + ACCOA = MAL | ab + cd = abdc |  | F |  |  |
| R20 | AKG = 0.5 SUC + 0.5 SUC + CO2 | abcde = 0.5 bcde + 0.5 edcb + a |  | F |  |  |
| R21 | SUC = MAL | abcd = abcd |  | FR |  |  |
| R22 | MAL = 0.5 SUC + 0.5 SUC | abcd = 0.5 abcd + 0.5 dcba |  | R | X |  |
| R23 | MAL = OAA | abcd = abcd |  | FR |  |  |
| R24 | OAA = MAL | abcd = abcd |  | R | X |  |
| R25 | MAL = PYR + CO2 | abcd = abc + d |  | F | X |  |
| R26 | PEP + CARB = OAA | abc + d = abcd |  | F | X |  |
| R27 | OAA = PEP + CO2 | abcd = abc + d |  | F |  |  |
| R28 | CARB_EX_1 = CARB | a = a |  | F | X |  |
| R29 | CARB_EX_2 = CARB | a = a |  | F |  |  |
| R30 | GLC6P = GLC6P_B |  |  | B | 0.017342 | 0.000867 |
| R31 | F6P = F6P_B |  |  | B | 0.005974 | 0.000299 |
| R32 | P5P = P5P_B |  |  | B | 0.074595 | 0.00373 |
| R33 | E4P = E4P_B |  |  | B | 0.029954 | 0.001498 |
| R34 | G3P = G3P_B |  |  | B | 0.010289 | 0.000514 |
| R35 | 3PG = 3PG_B |  |  | B | 0.133923 | 0.006696 |
| R36 | PEP = PEP_B |  |  | B | 0.06509 | 0.003255 |
| R37 | PYR = PYR_B |  |  | B | 0.245487 | 0.012274 |
| R38 | ACCOA = ACCOA_B |  |  | B | 0.202211 | 0.010111 |
| R39 | OAA = OAA_B |  |  | B | 0.135499 | 0.006775 |
| R40 | AKG = AKG_B |  |  | B | 0.106955 | 0.005348 |
| R41 | ACCOA = ACE_EX |  |  | B | X |  |
| R42 | AKG + AKG = HYP + SUC_II | abcde + fghij = abcde + fghij |  | F |  |  |
| R43 | SUC_II = 0.5 SUC + 0.5 SUC + CO2 | abcde = 0.5 bcde + 0.5 edcb + a |  | F |  |  |
| R44 | HYP = HYP_EX |  |  | B | 0 |  |
| R45 | PYR + PYR = VALX + CO2X | abc + def = abefc + d |  | S |  |  |
| R46 | E4P + PEP = SHKM | abcd + efg = efgabcd |  | S |  |  |
| R47 | SHKM + PEP = CHRM | abcdefg + hij = abcdefghij |  | S |  |  |
| R48 | CHRM = PHEX + CO2X | abcdefghij = hijbcdefg + a |  | S |  |  |
| R49 | CHRM = TYRX + CO2 | abcdefghij = hijbcdefg + a |  | S |  |  |
| R50 | PYR = ALAX | abc = abc |  | S |  |  |
| R51 | OAA = THRX | abcd = abcd |  | S |  |  |
| R52 | OAA = ASPX | abcd = abcd |  | S |  |  |
| R53 | AKG = GLUX | abcde = abcde |  | S |  |  |
| R54 | 3PG = SERX | abc = abc |  | S |  |  |
| R55 | 3PG = GLYX + MTHF | abc = ab + c |  | S |  |  |
| R56 | PEP + OAA = ILEX + CO2X | abc + defg = debfgc + a |  | S |  |  |
| R57 | PEP + PEP = ISV + CO2X | abc + def = abefc + d |  | S |  |  |
| R58 | ISV + ACCOA = LEUX + CO2X | abcde + fg = fgbcde + a |  | S |  |  |
|  |  |  |  |  |  |  |
| ## | excludedMetabolites |  |  |  |  |  |
| # | MTHF |  |  |  |  |  |
| # | GLC_EX |  |  |  |  |  |
| # | ACE_EX |  |  |  |  |  |
| # | CO2 |  |  |  |  |  |
| # | CARB_EX_1 |  |  |  |  |  |
| # | CARB_EX_2 |  |  |  |  |  |
| # | HYP_EX |  |  |  |  |  |
| # | GLC6P_B |  |  |  |  |  |
| # | F6P_B |  |  |  |  |  |
| # | P5P_B |  |  |  |  |  |
| # | E4P_B |  |  |  |  |  |
| # | G3P_B |  |  |  |  |  |
| # | 3PG_B |  |  |  |  |  |
| # | PEP_B |  |  |  |  |  |
| # | PYR_B |  |  |  |  |  |
| # | ACCOA_B |  |  |  |  |  |
| # | OAA_B |  |  |  |  |  |
| # | AKG_B |  |  |  |  |  |
|  |  |  |  |  |  |  |
| ## | simulatedMDVs |  |  |  |  |  |
| # | ALAX#111 |  |  |  |  |  |
| # | ALAX#011 |  |  |  |  |  |
| # | GLYX#11 |  |  |  |  |  |
| # | GLYX#01 |  |  |  |  |  |
| # | VALX#11111 |  |  |  |  |  |
| # | VALX#01111 |  |  |  |  |  |
| # | LEUX#011111 |  |  |  |  |  |
| # | ILEX#011111 |  |  |  |  |  |
| # | SERX#111 |  |  |  |  |  |
| # | SERX#011 |  |  |  |  |  |
| # | THRX#1111 |  |  |  |  |  |
| # | THRX#0111 |  |  |  |  |  |
| # | PHEX#111111111 |  |  |  |  |  |
| # | PHEX#011111111 |  |  |  |  |  |
| # | ASPX#1111 |  |  |  |  |  |
| # | GLUX#11111 |  |  |  |  |  |
| # | TYRX#111111111 |  |  |  |  |  |
|  |  |  |  |  |  |  |
| ## | inputSubstrates |  |  |  |  |  |
| # | CARB_EX_1 |  |  |  |  |  |
| # | CARB_EX_2 |  |  |  |  |  |
| # | GLC_EX |  |  |  |  |  |
|  |  |  |  |  |  |  |
| ## | measurements |  |  |  |  |  |
| # | 0.366328433 | Ala 260 | m |  |  |  |
| # | 0.34640987 |  | m+1 |  |  |  |
| # | 0.11971838 |  | m+2 |  |  |  |
| # | 0.388788445 | Ala 232 | m |  |  |  |
| # | 0.36538967 |  | m+1 |  |  |  |
| # | 0.245821884 |  | m+2 |  |  |  |
| # | 0.621504616 | Gly 246 | m |  |  |  |
| # | 0.17372232 |  | m+1 |  |  |  |
| # | 0.692024467 | Gly 218 | m |  |  |  |
| # | 0.307975533 |  | m+1 |  |  |  |
| # | 0.174908791 | Val 288 | m |  |  |  |
| # | 0.277975881 |  | m+1 |  |  |  |
| # | 0.232152578 |  | m+2 |  |  |  |
| # | 0.182894525 | Val 260 | m |  |  |  |
| # | 0.286236988 |  | m+1 |  |  |  |
| # | 0.289219571 |  | m+2 |  |  |  |
| # | 0.113287353 | Leu 200 | m |  |  |  |
| # | 0.24260516 |  | m+1 |  |  |  |
| # | 0.292117195 |  | m+2 |  |  |  |
| # | 0.139920686 | Ile 200 | m |  |  |  |
| # | 0.26044346 |  | m+1 |  |  |  |
| # | 0.287066359 |  | m+2 |  |  |  |
| # | 0.319308002 | Ser 390 | m |  |  |  |
| # | 0.34924585 |  | m+1 |  |  |  |
| # | 0.166724583 |  | m+2 |  |  |  |
| # | 0.346368728 | Ser 362 | m |  |  |  |
| # | 0.392266667 |  | m+1 |  |  |  |
| # | 0.261364605 |  | m+2 |  |  |  |
| # | 0.193139592 | Thr 404 | m |  |  |  |
| # | 0.296405532 |  | m+1 |  |  |  |
| # | 0.246661938 |  | m+2 |  |  |  |
| # | 0.237438317 | Thr 376 | m |  |  |  |
| # | 0.343227874 |  | m+1 |  |  |  |
| # | 0.272542243 |  | m+2 |  |  |  |
| # | 0.111204206 | Phe 336 | m |  |  |  |
| # | 0.194934461 |  | m+1 |  |  |  |
| # | 0.19217076 |  | m+2 |  |  |  |
| # | 0.125517085 | Phe 234 | m |  |  |  |
| # | 0.211307355 |  | m+1 |  |  |  |
| # | 0.231076055 |  | m+2 |  |  |  |
| # | 0.194496335 | Asp 418 | m |  |  |  |
| # | 0.30036584 |  | m+1 |  |  |  |
| # | 0.246259529 |  | m+2 |  |  |  |
| # | 0.114594991 | Glu 432 | m |  |  |  |
| # | 0.233735778 |  | m+1 |  |  |  |
| # | 0.273314275 |  | m+2 |  |  |  |
| # | 0.09807566 | Tyr 466 | m |  |  |  |
| # | 0.181317497 |  | m+1 |  |  |  |
| # | 0.189723497 |  | m+2 |  |  |  |
|  |  |  |  |  |  |  |
| ## | error |  |  |  |  |  |
| # | 0.001230642 | Ala 260 | m |  |  |  |
| # | 0.000726746 |  | m+1 |  |  |  |
| # | 0.001588049 |  | m+2 |  |  |  |
| # | 0.002344672 | Ala 232 | m |  |  |  |
| # | 0.00080633 |  | m+1 |  |  |  |
| # | 0.001538342 |  | m+2 |  |  |  |
| # | 0.001530466 | Gly 246 | m |  |  |  |
| # | 0.001894043 |  | m+1 |  |  |  |
| # | 0.000190404 | Gly 218 | m |  |  |  |
| # | 0.000190404 |  | m+1 |  |  |  |
| # | 0.003428503 | Val 288 | m |  |  |  |
| # | 0.001622756 |  | m+1 |  |  |  |
| # | 0.000579387 |  | m+2 |  |  |  |
| # | 0.001528834 | Val 260 | m |  |  |  |
| # | 0.000854257 |  | m+1 |  |  |  |
| # | 0.000779967 |  | m+2 |  |  |  |
| # | 0.000814388 | Leu 200 | m |  |  |  |
| # | 0.00135206 |  | m+1 |  |  |  |
| # | 0.001899986 |  | m+2 |  |  |  |
| # | 0.001095267 | Ile 200 | m |  |  |  |
| # | 0.001895653 |  | m+1 |  |  |  |
| # | 0.000617373 |  | m+2 |  |  |  |
| # | 0.002342803 | Ser 390 | m |  |  |  |
| # | 0.00056526 |  | m+1 |  |  |  |
| # | 0.000606455 |  | m+2 |  |  |  |
| # | 0.001862673 | Ser 362 | m |  |  |  |
| # | 0.001111055 |  | m+1 |  |  |  |
| # | 0.000751618 |  | m+2 |  |  |  |
| # | 0.001199573 | Thr 404 | m |  |  |  |
| # | 0.000483112 |  | m+1 |  |  |  |
| # | 0.00189696 |  | m+2 |  |  |  |
| # | 0.003200555 | Thr 376 | m |  |  |  |
| # | 0.001180982 |  | m+1 |  |  |  |
| # | 0.000932888 |  | m+2 |  |  |  |
| # | 0.00181061 | Phe 336 | m |  |  |  |
| # | 0.00026848 |  | m+1 |  |  |  |
| # | 0.000449017 |  | m+2 |  |  |  |
| # | 0.002033262 | Phe 234 | m |  |  |  |
| # | 0.001689744 |  | m+1 |  |  |  |
| # | 0.001791977 |  | m+2 |  |  |  |
| # | 0.003022816 | Asp 418 | m |  |  |  |
| # | 0.000627193 |  | m+1 |  |  |  |
| # | 0.001044023 |  | m+2 |  |  |  |
| # | 0.002583245 | Glu 432 | m |  |  |  |
| # | 0.000257235 |  | m+1 |  |  |  |
| # | 0.000599599 |  | m+2 |  |  |  |
| # | 0.000245453 | Tyr 466 | m |  |  |  |
| # | 0.002326374 |  | m+1 |  |  |  |
| # | 0.001110388 |  | m+2 |  |  |  |

**BL21*ΔputA* (DE3) (pLysS) (pET-24a) on glucose model**

| rxnID | rxnEQ | cTrans | rates | type | basis | deviation |
| --- | --- | --- | --- | --- | --- | --- |
| R01 | GLC_EX = GLC6P | abcdef = abcdef |  | F | 1 | 0 |
| R02 | GLC6P = F6P | abcdef = abcdef |  | FR |  |  |
| R03 | F6P=GLC6P | abcdef = abcdef |  | R | x |  |
| R04 | F6P = DHAP + G3P | abcdef = abc + def |  | F |  |  |
| R05 | DHAP = G3P | abc = cba |  | F |  |  |
| R06 | GLC6P = P5P + CO2 | abcdef = bcdef + a |  | F | X |  |
| R07 | P5P + P5P = S7P + G3P | abcde + fghij = fgabcde + hij |  | F |  |  |
| R08 | S7P + G3P = E4P + F6P | abcdefg + hij = defg + abchij |  | F |  |  |
| R09 | E4P + P5P = F6P + G3P | abcd + efghi = efabcd + ghi |  | F |  |  |
| R10 | G3P = 3PG | abc = abc |  | F |  |  |
| R11 | 3PG = PEP | abc = abc |  | FR |  |  |
| R12 | PEP = 3PG | abc = abc |  | R | X |  |
| R13 | PEP = PYR | abc = abc |  | F |  |  |
| R14 | PYR = ACCOA + CO2 | abc = bc + a |  | F |  |  |
| R15 | ACCOA + OAA = CIT | ab + cdef = fedbac |  | F |  |  |
| R16 | CIT = AKG + CO2 | abcdef = abcde + f |  | F |  |  |
| R17 | CIT = S_PREC + GLYOX | abcdef = cdef + ab |  | F | 0 |  |
| R18 | S_PREC = 0.5 SUC + 0.5 SUC | abcd = 0.5 abcd + 0.5 dcba |  | F |  |  |
| R19 | GLYOX + ACCOA = MAL | ab + cd = abdc |  | F |  |  |
| R20 | AKG = 0.5 SUC + 0.5 SUC + CO2 | abcde = 0.5 bcde + 0.5 edcb + a |  | F |  |  |
| R21 | SUC = MAL | abcd = abcd |  | FR |  |  |
| R22 | MAL = 0.5 SUC + 0.5 SUC | abcd = 0.5 abcd + 0.5 dcba |  | R | X |  |
| R23 | MAL = OAA | abcd = abcd |  | FR |  |  |
| R24 | OAA = MAL | abcd = abcd |  | R | X |  |
| R25 | MAL = PYR + CO2 | abcd = abc + d |  | F | X |  |
| R26 | PEP + CARB = OAA | abc + d = abcd |  | F | X |  |
| R27 | OAA = PEP + CO2 | abcd = abc + d |  | F |  |  |
| R28 | CARB_EX_1 = CARB | a = a |  | F | X |  |
| R29 | CARB_EX_2 = CARB | a = a |  | F |  |  |
| R30 | GLC6P = GLC6P_B |  |  | B | 0.017102 | 0.00085511 |
| R31 | F6P = F6P_B |  |  | B | 0.005892 | 0.000294583 |
| R32 | P5P = P5P_B |  |  | B | 0.073564 | 0.003678198 |
| R33 | E4P = E4P_B |  |  | B | 0.02954 | 0.001477007 |
| R34 | G3P = G3P_B |  |  | B | 0.010147 | 0.000507338 |
| R35 | 3PG = 3PG_B |  |  | B | 0.132071 | 0.006603573 |
| R36 | PEP = PEP_B |  |  | B | 0.064191 | 0.003209527 |
| R37 | PYR = PYR_B |  |  | B | 0.242094 | 0.012104707 |
| R38 | ACCOA = ACCOA_B |  |  | B | 0.199416 | 0.009970823 |
| R39 | OAA = OAA_B |  |  | B | 0.133626 | 0.00668131 |
| R40 | AKG = AKG_B |  |  | B | 0.105477 | 0.005273857 |
| R41 | ACCOA = ACE_EX |  |  | B | X |  |
| R42 | AKG + AKG = HYP + SUC_II | abcde + fghij = abcde + fghij |  | F |  |  |
| R43 | SUC_II = 0.5 SUC + 0.5 SUC + CO2 | abcde = 0.5 bcde + 0.5 edcb + a |  | F |  |  |
| R44 | HYP = HYP_EX |  |  | B | 0 |  |
| R45 | PYR + PYR = VALX + CO2X | abc + def = abefc + d |  | S |  |  |
| R46 | E4P + PEP = SHKM | abcd + efg = efgabcd |  | S |  |  |
| R47 | SHKM + PEP = CHRM | abcdefg + hij = abcdefghij |  | S |  |  |
| R48 | CHRM = PHEX + CO2X | abcdefghij = hijbcdefg + a |  | S |  |  |
| R49 | CHRM = TYRX + CO2 | abcdefghij = hijbcdefg + a |  | S |  |  |
| R50 | PYR = ALAX | abc = abc |  | S |  |  |
| R51 | OAA = THRX | abcd = abcd |  | S |  |  |
| R52 | OAA = ASPX | abcd = abcd |  | S |  |  |
| R53 | AKG = GLUX | abcde = abcde |  | S |  |  |
| R54 | 3PG = SERX | abc = abc |  | S |  |  |
| R55 | 3PG = GLYX + MTHF | abc = ab + c |  | S |  |  |
| R56 | PEP + OAA = ILEX + CO2X | abc + defg = debfgc + a |  | S |  |  |
| R57 | PEP + PEP = ISV + CO2X | abc + def = abefc + d |  | S |  |  |
| R58 | ISV + ACCOA = LEUX + CO2X | abcde + fg = fgbcde + a |  | S |  |  |
|  |  |  |  |  |  |  |
| ## | excludedMetabolites |  |  |  |  |  |
| # | MTHF |  |  |  |  |  |
| # | GLC_EX |  |  |  |  |  |
| # | ACE_EX |  |  |  |  |  |
| # | CO2 |  |  |  |  |  |
| # | CARB_EX_1 |  |  |  |  |  |
| # | CARB_EX_2 |  |  |  |  |  |
| # | HYP_EX |  |  |  |  |  |
| # | GLC6P_B |  |  |  |  |  |
| # | F6P_B |  |  |  |  |  |
| # | P5P_B |  |  |  |  |  |
| # | E4P_B |  |  |  |  |  |
| # | G3P_B |  |  |  |  |  |
| # | 3PG_B |  |  |  |  |  |
| # | PEP_B |  |  |  |  |  |
| # | PYR_B |  |  |  |  |  |
| # | ACCOA_B |  |  |  |  |  |
| # | OAA_B |  |  |  |  |  |
| # | AKG_B |  |  |  |  |  |
|  |  |  |  |  |  |  |
| ## | simulatedMDVs |  |  |  |  |  |
| # | ALAX#111 |  |  |  |  |  |
| # | ALAX#011 |  |  |  |  |  |
| # | GLYX#11 |  |  |  |  |  |
| # | GLYX#01 |  |  |  |  |  |
| # | VALX#11111 |  |  |  |  |  |
| # | VALX#01111 |  |  |  |  |  |
| # | LEUX#011111 |  |  |  |  |  |
| # | ILEX#011111 |  |  |  |  |  |
| # | SERX#111 |  |  |  |  |  |
| # | SERX#011 |  |  |  |  |  |
| # | THRX#1111 |  |  |  |  |  |
| # | THRX#0111 |  |  |  |  |  |
| # | PHEX#111111111 |  |  |  |  |  |
| # | PHEX#011111111 |  |  |  |  |  |
| # | ASPX#1111 |  |  |  |  |  |
| # | GLUX#11111 |  |  |  |  |  |
| # | TYRX#111111111 |  |  |  |  |  |
|  |  |  |  |  |  |  |
| ## | inputSubstrates |  |  |  |  |  |
| # | CARB_EX_1 |  |  |  |  |  |
| # | CARB_EX_2 |  |  |  |  |  |
| # | GLC_EX |  |  |  |  |  |
|  |  |  |  |  |  |  |
| ## | measurements |  |  |  |  |  |
| # | 0.366152463 | Ala 260 | m |  |  |  |
| # | 0.346002255 |  | m+1 |  |  |  |
| # | 0.120711409 |  | m+2 |  |  |  |
| # | 0.390829454 | Ala 232 | m |  |  |  |
| # | 0.364039314 |  | m+1 |  |  |  |
| # | 0.245131232 |  | m+2 |  |  |  |
| # | 0.620777561 | Gly 246 | m |  |  |  |
| # | 0.174994377 |  | m+1 |  |  |  |
| # | 0.692888427 | Gly 218 | m |  |  |  |
| # | 0.307111573 |  | m+1 |  |  |  |
| # | 0.176447884 | Val 288 | m |  |  |  |
| # | 0.278418551 |  | m+1 |  |  |  |
| # | 0.230090599 |  | m+2 |  |  |  |
| # | 0.183574023 | Val 260 | m |  |  |  |
| # | 0.286710918 |  | m+1 |  |  |  |
| # | 0.288634702 |  | m+2 |  |  |  |
| # | 0.114211905 | Leu 200 | m |  |  |  |
| # | 0.24079165 |  | m+1 |  |  |  |
| # | 0.290476982 |  | m+2 |  |  |  |
| # | 0.140119193 | Ile 200 | m |  |  |  |
| # | 0.258439609 |  | m+1 |  |  |  |
| # | 0.285356988 |  | m+2 |  |  |  |
| # | 0.320769083 | Ser 390 | m |  |  |  |
| # | 0.348132967 |  | m+1 |  |  |  |
| # | 0.167066759 |  | m+2 |  |  |  |
| # | 0.345811699 | Ser 362 | m |  |  |  |
| # | 0.392182809 |  | m+1 |  |  |  |
| # | 0.262005491 |  | m+2 |  |  |  |
| # | 0.190252306 | Thr 404 | m |  |  |  |
| # | 0.295974194 |  | m+1 |  |  |  |
| # | 0.248221086 |  | m+2 |  |  |  |
| # | 0.232462333 | Thr 376 | m |  |  |  |
| # | 0.343359981 |  | m+1 |  |  |  |
| # | 0.27683809 |  | m+2 |  |  |  |
| # | 0.111145524 | Phe 336 | m |  |  |  |
| # | 0.193640094 |  | m+1 |  |  |  |
| # | 0.191803533 |  | m+2 |  |  |  |
| # | 0.125089738 | Phe 234 | m |  |  |  |
| # | 0.211712892 |  | m+1 |  |  |  |
| # | 0.231585654 |  | m+2 |  |  |  |
| # | 0.193536989 | Asp 418 | m |  |  |  |
| # | 0.295117836 |  | m+1 |  |  |  |
| # | 0.249320984 |  | m+2 |  |  |  |
| # | 0.115386898 | Glu 432 | m |  |  |  |
| # | 0.233638682 |  | m+1 |  |  |  |
| # | 0.269326017 |  | m+2 |  |  |  |
| # | 0.09581945 | Tyr 466 | m |  |  |  |
| # | 0.182648285 |  | m+1 |  |  |  |
| # | 0.192044472 |  | m+2 |  |  |  |
|  |  |  |  |  |  |  |
| ## | error |  |  |  |  |  |
| # | 0.0028391 | Ala 260 | m |  |  |  |
| # | 0.0003399 |  | m+1 |  |  |  |
| # | 0.0018739 |  | m+2 |  |  |  |
| # | 0.0005776 | Ala 232 | m |  |  |  |
| # | 0.0004534 |  | m+1 |  |  |  |
| # | 0.0001241 |  | m+2 |  |  |  |
| # | 0.00162 | Gly 246 | m |  |  |  |
| # | 0.0001725 |  | m+1 |  |  |  |
| # | 0.0001637 | Gly 218 | m |  |  |  |
| # | 0.0001637 |  | m+1 |  |  |  |
| # | 0.0011463 | Val 288 | m |  |  |  |
| # | 0.0020458 |  | m+1 |  |  |  |
| # | 0.0029283 |  | m+2 |  |  |  |
| # | 0.0015527 | Val 260 | m |  |  |  |
| # | 0.0004096 |  | m+1 |  |  |  |
| # | 0.0005322 |  | m+2 |  |  |  |
| # | 0.0002521 | Leu 200 | m |  |  |  |
| # | 0.0013761 |  | m+1 |  |  |  |
| # | 0.0006455 |  | m+2 |  |  |  |
| # | 0.0001917 | Ile 200 | m |  |  |  |
| # | 0.0018924 |  | m+1 |  |  |  |
| # | 0.0000595 |  | m+2 |  |  |  |
| # | 0.0017856 | Ser 390 | m |  |  |  |
| # | 0.0019106 |  | m+1 |  |  |  |
| # | 0.0004199 |  | m+2 |  |  |  |
| # | 0.0028965 | Ser 362 | m |  |  |  |
| # | 0.0009154 |  | m+1 |  |  |  |
| # | 0.0019811 |  | m+2 |  |  |  |
| # | 0.000018 | Thr 404 | m |  |  |  |
| # | 0.001309 |  | m+1 |  |  |  |
| # | 0.0005396 |  | m+2 |  |  |  |
| # | 0.001723 | Thr 376 | m |  |  |  |
| # | 0.0020287 |  | m+1 |  |  |  |
| # | 0.0011155 |  | m+2 |  |  |  |
| # | 0.0031559 | Phe 336 | m |  |  |  |
| # | 0.0035192 |  | m+1 |  |  |  |
| # | 0.0016103 |  | m+2 |  |  |  |
| # | 0.001094 | Phe 234 | m |  |  |  |
| # | 0.0000609 |  | m+1 |  |  |  |
| # | 0.0002504 |  | m+2 |  |  |  |
| # | 0.0010145 | Asp 418 | m |  |  |  |
| # | 0.0023528 |  | m+1 |  |  |  |
| # | 0.0010522 |  | m+2 |  |  |  |
| # | 0.0013364 | Glu 432 | m |  |  |  |
| # | 0.001161 |  | m+1 |  |  |  |
| # | 0.0039195 |  | m+2 |  |  |  |
| # | 0.0019919 | Tyr 466 | m |  |  |  |
| # | 0.000476 |  | m+1 |  |  |  |
| # | 0.0012662 |  | m+2 |  |  |  |

**BL21(DE3) (pLysS) (pET-p4h1of) on glucose model**

| rxnID | rxnEQ | cTrans | rates | type | basis | deviation |
| --- | --- | --- | --- | --- | --- | --- |
| R01 | GLC_EX = GLC6P | abcdef = abcdef |  | F | 1 | 0 |
| R02 | GLC6P = F6P | abcdef = abcdef |  | FR |  |  |
| R03 | F6P=GLC6P | abcdef = abcdef |  | R | x |  |
| R04 | F6P = DHAP + G3P | abcdef = abc + def |  | F |  |  |
| R05 | DHAP = G3P | abc = cba |  | F |  |  |
| R06 | GLC6P = P5P + CO2 | abcdef = bcdef + a |  | F | X |  |
| R07 | P5P + P5P = S7P + G3P | abcde + fghij = fgabcde + hij |  | F |  |  |
| R08 | S7P + G3P = E4P + F6P | abcdefg + hij = defg + abchij |  | F |  |  |
| R09 | E4P + P5P = F6P + G3P | abcd + efghi = efabcd + ghi |  | F |  |  |
| R10 | G3P = 3PG | abc = abc |  | F |  |  |
| R11 | 3PG = PEP | abc = abc |  | FR |  |  |
| R12 | PEP = 3PG | abc = abc |  | R | X |  |
| R13 | PEP = PYR | abc = abc |  | F |  |  |
| R14 | PYR = ACCOA + CO2 | abc = bc + a |  | F |  |  |
| R15 | ACCOA + OAA = CIT | ab + cdef = fedbac |  | F |  |  |
| R16 | CIT = AKG + CO2 | abcdef = abcde + f |  | F |  |  |
| R17 | CIT = S_PREC + GLYOX | abcdef = cdef + ab |  | F | 0 |  |
| R18 | S_PREC = 0.5 SUC + 0.5 SUC | abcd = 0.5 abcd + 0.5 dcba |  | F |  |  |
| R19 | GLYOX + ACCOA = MAL | ab + cd = abdc |  | F |  |  |
| R20 | AKG = 0.5 SUC + 0.5 SUC + CO2 | abcde = 0.5 bcde + 0.5 edcb + a |  | F |  |  |
| R21 | SUC = MAL | abcd = abcd |  | FR |  |  |
| R22 | MAL = 0.5 SUC + 0.5 SUC | abcd = 0.5 abcd + 0.5 dcba |  | R | X |  |
| R23 | MAL = OAA | abcd = abcd |  | FR |  |  |
| R24 | OAA = MAL | abcd = abcd |  | R | X |  |
| R25 | MAL = PYR + CO2 | abcd = abc + d |  | F | X |  |
| R26 | PEP + CARB = OAA | abc + d = abcd |  | F | X |  |
| R27 | OAA = PEP + CO2 | abcd = abc + d |  | F |  |  |
| R28 | CARB_EX_1 = CARB | a = a |  | F | X |  |
| R29 | CARB_EX_2 = CARB | a = a |  | F |  |  |
| R30 | GLC6P = GLC6P_B |  |  | B | 0.014879 | 0.000744 |
| R31 | F6P = F6P_B |  |  | B | 0.005126 | 0.000256 |
| R32 | P5P = P5P_B |  |  | B | 0.064002 | 0.0032 |
| R33 | E4P = E4P_B |  |  | B | 0.0257 | 0.001285 |
| R34 | G3P = G3P_B |  |  | B | 0.008828 | 0.000441 |
| R35 | 3PG = 3PG_B |  |  | B | 0.114904 | 0.005745 |
| R36 | PEP = PEP_B |  |  | B | 0.055847 | 0.002792 |
| R37 | PYR = PYR_B |  |  | B | 0.210625 | 0.010531 |
| R38 | ACCOA = ACCOA_B |  |  | B | 0.173495 | 0.008675 |
| R39 | OAA = OAA_B |  |  | B | 0.116257 | 0.005813 |
| R40 | AKG = AKG_B |  |  | B | 0.07066 | 0.003533 |
| R41 | ACCOA = ACE_EX |  |  | B | X |  |
| R42 | AKG + AKG = HYP + SUC_II | abcde + fghij = abcde + fghij |  | F |  |  |
| R43 | SUC_II = 0.5 SUC + 0.5 SUC + CO2 | abcde = 0.5 bcde + 0.5 edcb + a |  | F |  |  |
| R44 | HYP = HYP_EX | abcde = abcde |  | F | X |  |
| R45 | PYR + PYR = VALX + CO2X | abc + def = abefc + d |  | S |  |  |
| R46 | E4P + PEP = SHKM | abcd + efg = efgabcd |  | S |  |  |
| R47 | SHKM + PEP = CHRM | abcdefg + hij = abcdefghij |  | S |  |  |
| R48 | CHRM = PHEX + CO2X | abcdefghij = hijbcdefg + a |  | S |  |  |
| R49 | CHRM = TYRX + CO2 | abcdefghij = hijbcdefg + a |  | S |  |  |
| R50 | PYR = ALAX | abc = abc |  | S |  |  |
| R51 | OAA = THRX | abcd = abcd |  | S |  |  |
| R52 | OAA = ASPX | abcd = abcd |  | S |  |  |
| R53 | AKG = GLUX | abcde = abcde |  | S |  |  |
| R54 | 3PG = SERX | abc = abc |  | S |  |  |
| R55 | 3PG = GLYX + MTHF | abc = ab + c |  | S |  |  |
| R56 | PEP + OAA = ILEX + CO2X | abc + defg = debfgc + a |  | S |  |  |
| R57 | PEP + PEP = ISV + CO2X | abc + def = abefc + d |  | S |  |  |
| R58 | ISV + ACCOA = LEUX + CO2X | abcde + fg = fgbcde + a |  | S |  |  |
|  |  |  |  |  |  |  |
| ## | excludedMetabolites |  |  |  |  |  |
| # | MTHF |  |  |  |  |  |
| # | GLC_EX |  |  |  |  |  |
| # | ACE_EX |  |  |  |  |  |
| # | CO2 |  |  |  |  |  |
| # | CARB_EX_1 |  |  |  |  |  |
| # | CARB_EX_2 |  |  |  |  |  |
| # | HYP_EX |  |  |  |  |  |
| # | GLC6P_B |  |  |  |  |  |
| # | F6P_B |  |  |  |  |  |
| # | P5P_B |  |  |  |  |  |
| # | E4P_B |  |  |  |  |  |
| # | G3P_B |  |  |  |  |  |
| # | 3PG_B |  |  |  |  |  |
| # | PEP_B |  |  |  |  |  |
| # | PYR_B |  |  |  |  |  |
| # | ACCOA_B |  |  |  |  |  |
| # | OAA_B |  |  |  |  |  |
| # | AKG_B |  |  |  |  |  |
|  |  |  |  |  |  |  |
| ## | simulatedMDVs |  |  |  |  |  |
| # | ALAX#111 |  |  |  |  |  |
| # | ALAX#011 |  |  |  |  |  |
| # | GLYX#11 |  |  |  |  |  |
| # | GLYX#01 |  |  |  |  |  |
| # | VALX#11111 |  |  |  |  |  |
| # | VALX#01111 |  |  |  |  |  |
| # | LEUX#011111 |  |  |  |  |  |
| # | ILEX#011111 |  |  |  |  |  |
| # | SERX#111 |  |  |  |  |  |
| # | SERX#011 |  |  |  |  |  |
| # | THRX#1111 |  |  |  |  |  |
| # | THRX#0111 |  |  |  |  |  |
| # | PHEX#111111111 |  |  |  |  |  |
| # | PHEX#011111111 |  |  |  |  |  |
| # | ASPX#1111 |  |  |  |  |  |
| # | GLUX#11111 |  |  |  |  |  |
| # | TYRX#111111111 |  |  |  |  |  |
|  |  |  |  |  |  |  |
| ## | inputSubstrates |  |  |  |  |  |
| # | CARB_EX_1 |  |  |  |  |  |
| # | CARB_EX_2 |  |  |  |  |  |
| # | GLC_EX |  |  |  |  |  |
|  |  |  |  |  |  |  |
| ## | measurements |  |  |  |  |  |
| # | 0.364893238 | Ala 260 | m |  |  |  |
| # | 0.346600967 |  | m+1 |  |  |  |
| # | 0.121083224 |  | m+2 |  |  |  |
| # | 0.387322514 | Ala 232 | m |  |  |  |
| # | 0.367172213 |  | m+1 |  |  |  |
| # | 0.245505273 |  | m+2 |  |  |  |
| # | 0.620678111 | Gly 246 | m |  |  |  |
| # | 0.174998489 |  | m+1 |  |  |  |
| # | 0.692309317 | Gly 218 | m |  |  |  |
| # | 0.307690683 |  | m+1 |  |  |  |
| # | 0.173929577 | Val 288 | m |  |  |  |
| # | 0.277305197 |  | m+1 |  |  |  |
| # | 0.232881908 |  | m+2 |  |  |  |
| # | 0.181649854 | Val 260 | m |  |  |  |
| # | 0.286156526 |  | m+1 |  |  |  |
| # | 0.288798066 |  | m+2 |  |  |  |
| # | 0.11289447 | Leu 200 | m |  |  |  |
| # | 0.23700813 |  | m+1 |  |  |  |
| # | 0.29046615 |  | m+2 |  |  |  |
| # | 0.137262717 | Ile 200 | m |  |  |  |
| # | 0.255356788 |  | m+1 |  |  |  |
| # | 0.285613651 |  | m+2 |  |  |  |
| # | 0.313348487 | Ser 390 | m |  |  |  |
| # | 0.352691242 |  | m+1 |  |  |  |
| # | 0.168950616 |  | m+2 |  |  |  |
| # | 0.341979197 | Ser 362 | m |  |  |  |
| # | 0.395278567 |  | m+1 |  |  |  |
| # | 0.262742236 |  | m+2 |  |  |  |
| # | 0.186999436 | Thr 404 | m |  |  |  |
| # | 0.295705178 |  | m+1 |  |  |  |
| # | 0.248226677 |  | m+2 |  |  |  |
| # | 0.230708762 | Thr 376 | m |  |  |  |
| # | 0.343402117 |  | m+1 |  |  |  |
| # | 0.278777016 |  | m+2 |  |  |  |
| # | 0.112786984 | Phe 336 | m |  |  |  |
| # | 0.196086514 |  | m+1 |  |  |  |
| # | 0.189631354 |  | m+2 |  |  |  |
| # | 0.12827003 | Phe 234 | m |  |  |  |
| # | 0.214486688 |  | m+1 |  |  |  |
| # | 0.232579602 |  | m+2 |  |  |  |
| # | 0.190794508 | Asp 418 | m |  |  |  |
| # | 0.29389883 |  | m+1 |  |  |  |
| # | 0.249561511 |  | m+2 |  |  |  |
| # | 0.113134693 | Glu 432 | m |  |  |  |
| # | 0.230684197 |  | m+1 |  |  |  |
| # | 0.269286715 |  | m+2 |  |  |  |
| # | 0.098569982 | Tyr 466 | m |  |  |  |
| # | 0.182152775 |  | m+1 |  |  |  |
| # | 0.19070063 |  | m+2 |  |  |  |
|  |  |  |  |  |  |  |
| ## | error |  |  |  |  |  |
| # | 0.001569722 | Ala 260 | m |  |  |  |
| # | 0.000655902 |  | m+1 |  |  |  |
| # | 0.000177722 |  | m+2 |  |  |  |
| # | 4.96E-05 | Ala 232 | m |  |  |  |
| # | 0.00061353 |  | m+1 |  |  |  |
| # | 0.000663105 |  | m+2 |  |  |  |
| # | 0.000448077 | Gly 246 | m |  |  |  |
| # | 0.001134178 |  | m+1 |  |  |  |
| # | 0.000301657 | Gly 218 | m |  |  |  |
| # | 0.000301657 |  | m+1 |  |  |  |
| # | 0.001321823 | Val 288 | m |  |  |  |
| # | 0.001921333 |  | m+1 |  |  |  |
| # | 0.003074577 |  | m+2 |  |  |  |
| # | 0.00084648 | Val 260 | m |  |  |  |
| # | 0.000745246 |  | m+1 |  |  |  |
| # | 0.000807585 |  | m+2 |  |  |  |
| # | 0.00020492 | Leu 200 | m |  |  |  |
| # | 0.000464568 |  | m+1 |  |  |  |
| # | 1.96E-05 |  | m+2 |  |  |  |
| # | 9.47E-05 | Ile 200 | m |  |  |  |
| # | 0.000134868 |  | m+1 |  |  |  |
| # | 0.000394828 |  | m+2 |  |  |  |
| # | 0.001096738 | Ser 390 | m |  |  |  |
| # | 0.001146095 |  | m+1 |  |  |  |
| # | 0.000380918 |  | m+2 |  |  |  |
| # | 0.0039335 | Ser 362 | m |  |  |  |
| # | 0.002698656 |  | m+1 |  |  |  |
| # | 0.001234844 |  | m+2 |  |  |  |
| # | 9.11E-05 | Thr 404 | m |  |  |  |
| # | 0.001065288 |  | m+1 |  |  |  |
| # | 0.0008902 |  | m+2 |  |  |  |
| # | 0.000236785 | Thr 376 | m |  |  |  |
| # | 0.000121285 |  | m+1 |  |  |  |
| # | 0.000542079 |  | m+2 |  |  |  |
| # | 0.000529052 | Phe 336 | m |  |  |  |
| # | 0.00130163 |  | m+1 |  |  |  |
| # | 0.000825274 |  | m+2 |  |  |  |
| # | 0.000189593 | Phe 234 | m |  |  |  |
| # | 0.000638734 |  | m+1 |  |  |  |
| # | 2.00E-05 |  | m+2 |  |  |  |
| # | 0.000734223 | Asp 418 | m |  |  |  |
| # | 0.001033762 |  | m+1 |  |  |  |
| # | 0.000652458 |  | m+2 |  |  |  |
| # | 0.001465108 | Glu 432 | m |  |  |  |
| # | 0.000327331 |  | m+1 |  |  |  |
| # | 0.001738264 |  | m+2 |  |  |  |
| # | 0.001507827 | Tyr 466 | m |  |  |  |
| # | 0.000726143 |  | m+1 |  |  |  |
| # | 0.000655879 |  | m+2 |  |  |  |

**BL21 *ΔputA* (DE3) (pLysS) (pET-p4h1of) on glucose model**

| rxnID | rxnEQ | cTrans | rates | type | basis | deviation |
| --- | --- | --- | --- | --- | --- | --- |
| R01 | GLC_EX = GLC6P | abcdef = abcdef |  | F | 1 | 0 |
| R02 | GLC6P = F6P | abcdef = abcdef |  | FR |  |  |
| R03 | F6P=GLC6P | abcdef = abcdef |  | R | x |  |
| R04 | F6P = DHAP + G3P | abcdef = abc + def |  | F |  |  |
| R05 | DHAP = G3P | abc = cba |  | F |  |  |
| R06 | GLC6P = P5P + CO2 | abcdef = bcdef + a |  | F | X |  |
| R07 | P5P + P5P = S7P + G3P | abcde + fghij = fgabcde + hij |  | F |  |  |
| R08 | S7P + G3P = E4P + F6P | abcdefg + hij = defg + abchij |  | F |  |  |
| R09 | E4P + P5P = F6P + G3P | abcd + efghi = efabcd + ghi |  | F |  |  |
| R10 | G3P = 3PG | abc = abc |  | F |  |  |
| R11 | 3PG = PEP | abc = abc |  | FR |  |  |
| R12 | PEP = 3PG | abc = abc |  | R | X |  |
| R13 | PEP = PYR | abc = abc |  | F |  |  |
| R14 | PYR = ACCOA + CO2 | abc = bc + a |  | F |  |  |
| R15 | ACCOA + OAA = CIT | ab + cdef = fedbac |  | F |  |  |
| R16 | CIT = AKG + CO2 | abcdef = abcde + f |  | F |  |  |
| R17 | CIT = S_PREC + GLYOX | abcdef = cdef + ab |  | F | 0 |  |
| R18 | S_PREC = 0.5 SUC + 0.5 SUC | abcd = 0.5 abcd + 0.5 dcba |  | F |  |  |
| R19 | GLYOX + ACCOA = MAL | ab + cd = abdc |  | F |  |  |
| R20 | AKG = 0.5 SUC + 0.5 SUC + CO2 | abcde = 0.5 bcde + 0.5 edcb + a |  | F |  |  |
| R21 | SUC = MAL | abcd = abcd |  | FR |  |  |
| R22 | MAL = 0.5 SUC + 0.5 SUC | abcd = 0.5 abcd + 0.5 dcba |  | R | X |  |
| R23 | MAL = OAA | abcd = abcd |  | FR |  |  |
| R24 | OAA = MAL | abcd = abcd |  | R | X |  |
| R25 | MAL = PYR + CO2 | abcd = abc + d |  | F | X |  |
| R26 | PEP + CARB = OAA | abc + d = abcd |  | F | X |  |
| R27 | OAA = PEP + CO2 | abcd = abc + d |  | F |  |  |
| R28 | CARB_EX_1 = CARB | a = a |  | F | X |  |
| R29 | CARB_EX_2 = CARB | a = a |  | F |  |  |
| R30 | GLC6P = GLC6P_B |  |  | B | 0.013501 | 0.000675 |
| R31 | F6P = F6P_B |  |  | B | 0.004651 | 0.000233 |
| R32 | P5P = P5P_B |  |  | B | 0.058073 | 0.002904 |
| R33 | E4P = E4P_B |  |  | B | 0.023319 | 0.001166 |
| R34 | G3P = G3P_B |  |  | B | 0.00801 | 0.000401 |
| R35 | 3PG = 3PG_B |  |  | B | 0.104259 | 0.005213 |
| R36 | PEP = PEP_B |  |  | B | 0.050673 | 0.002534 |
| R37 | PYR = PYR_B |  |  | B | 0.191113 | 0.009556 |
| R38 | ACCOA = ACCOA_B |  |  | B | 0.157423 | 0.007871 |
| R39 | OAA = OAA_B |  |  | B | 0.105487 | 0.005274 |
| R40 | AKG = AKG_B |  |  | B | 0.064114 | 0.003206 |
| R41 | ACCOA = ACE_EX |  |  | B | X |  |
| R42 | AKG + AKG = HYP + SUC_II | abcde + fghij = abcde + fghij |  | F |  |  |
| R43 | SUC_II = 0.5 SUC + 0.5 SUC + CO2 | abcde = 0.5 bcde + 0.5 edcb + a |  | F |  |  |
| R44 | HYP = HYP_EX | abcde = abcde |  | F | X |  |
| R45 | PYR + PYR = VALX + CO2X | abc + def = abefc + d |  | S |  |  |
| R46 | E4P + PEP = SHKM | abcd + efg = efgabcd |  | S |  |  |
| R47 | SHKM + PEP = CHRM | abcdefg + hij = abcdefghij |  | S |  |  |
| R48 | CHRM = PHEX + CO2X | abcdefghij = hijbcdefg + a |  | S |  |  |
| R49 | CHRM = TYRX + CO2 | abcdefghij = hijbcdefg + a |  | S |  |  |
| R50 | PYR = ALAX | abc = abc |  | S |  |  |
| R51 | OAA = THRX | abcd = abcd |  | S |  |  |
| R52 | OAA = ASPX | abcd = abcd |  | S |  |  |
| R53 | AKG = GLUX | abcde = abcde |  | S |  |  |
| R54 | 3PG = SERX | abc = abc |  | S |  |  |
| R55 | 3PG = GLYX + MTHF | abc = ab + c |  | S |  |  |
| R56 | PEP + OAA = ILEX + CO2X | abc + defg = debfgc + a |  | S |  |  |
| R57 | PEP + PEP = ISV + CO2X | abc + def = abefc + d |  | S |  |  |
| R58 | ISV + ACCOA = LEUX + CO2X | abcde + fg = fgbcde + a |  | S |  |  |
|  |  |  |  |  |  |  |
| ## | excludedMetabolites |  |  |  |  |  |
| # | MTHF |  |  |  |  |  |
| # | GLC_EX |  |  |  |  |  |
| # | ACE_EX |  |  |  |  |  |
| # | CO2 |  |  |  |  |  |
| # | CARB_EX_1 |  |  |  |  |  |
| # | CARB_EX_2 |  |  |  |  |  |
| # | HYP_EX |  |  |  |  |  |
| # | GLC6P_B |  |  |  |  |  |
| # | F6P_B |  |  |  |  |  |
| # | P5P_B |  |  |  |  |  |
| # | E4P_B |  |  |  |  |  |
| # | G3P_B |  |  |  |  |  |
| # | 3PG_B |  |  |  |  |  |
| # | PEP_B |  |  |  |  |  |
| # | PYR_B |  |  |  |  |  |
| # | ACCOA_B |  |  |  |  |  |
| # | OAA_B |  |  |  |  |  |
| # | AKG_B |  |  |  |  |  |
|  |  |  |  |  |  |  |
| ## | simulatedMDVs |  |  |  |  |  |
| # | ALAX#111 |  |  |  |  |  |
| # | ALAX#011 |  |  |  |  |  |
| # | GLYX#11 |  |  |  |  |  |
| # | GLYX#01 |  |  |  |  |  |
| # | VALX#11111 |  |  |  |  |  |
| # | VALX#01111 |  |  |  |  |  |
| # | LEUX#011111 |  |  |  |  |  |
| # | ILEX#011111 |  |  |  |  |  |
| # | SERX#111 |  |  |  |  |  |
| # | SERX#011 |  |  |  |  |  |
| # | THRX#1111 |  |  |  |  |  |
| # | THRX#0111 |  |  |  |  |  |
| # | PHEX#111111111 |  |  |  |  |  |
| # | PHEX#011111111 |  |  |  |  |  |
| # | ASPX#1111 |  |  |  |  |  |
| # | GLUX#11111 |  |  |  |  |  |
| # | TYRX#111111111 |  |  |  |  |  |
|  |  |  |  |  |  |  |
| ## | inputSubstrates |  |  |  |  |  |
| # | CARB_EX_1 |  |  |  |  |  |
| # | CARB_EX_2 |  |  |  |  |  |
| # | GLC_EX |  |  |  |  |  |
|  |  |  |  |  |  |  |
| ## | measurements |  |  |  |  |  |
| # | 0.36108 | Ala 260 | m |  |  |  |
| # | 0.34916 |  | m+1 |  |  |  |
| # | 0.12204 |  | m+2 |  |  |  |
| # | 0.38357 | Ala 232 | m |  |  |  |
| # | 0.36897 |  | m+1 |  |  |  |
| # | 0.24746 |  | m+2 |  |  |  |
| # | 0.61922 | Gly 246 | m |  |  |  |
| # | 0.17658 |  | m+1 |  |  |  |
| # | 0.69252 | Gly 218 | m |  |  |  |
| # | 0.30748 |  | m+1 |  |  |  |
| # | 0.16904 | Val 288 | m |  |  |  |
| # | 0.27782 |  | m+1 |  |  |  |
| # | 0.23346 |  | m+2 |  |  |  |
| # | 0.17858 | Val 260 | m |  |  |  |
| # | 0.28678 |  | m+1 |  |  |  |
| # | 0.29121 |  | m+2 |  |  |  |
| # | 0.10924 | Leu 200 | m |  |  |  |
| # | 0.23983 |  | m+1 |  |  |  |
| # | 0.29326 |  | m+2 |  |  |  |
| # | 0.13459 | Ile 200 | m |  |  |  |
| # | 0.25922 |  | m+1 |  |  |  |
| # | 0.28701 |  | m+2 |  |  |  |
| # | 0.31338 | Ser 390 | m |  |  |  |
| # | 0.3514 |  | m+1 |  |  |  |
| # | 0.16977 |  | m+2 |  |  |  |
| # | 0.3404 | Ser 362 | m |  |  |  |
| # | 0.39577 |  | m+1 |  |  |  |
| # | 0.26383 |  | m+2 |  |  |  |
| # | 0.18392 | Thr 404 | m |  |  |  |
| # | 0.2973 |  | m+1 |  |  |  |
| # | 0.25003 |  | m+2 |  |  |  |
| # | 0.22902 | Thr 376 | m |  |  |  |
| # | 0.34475 |  | m+1 |  |  |  |
| # | 0.27656 |  | m+2 |  |  |  |
| # | 0.10879 | Phe 336 | m |  |  |  |
| # | 0.19614 |  | m+1 |  |  |  |
| # | 0.19096 |  | m+2 |  |  |  |
| # | 0.12412 | Phe 234 | m |  |  |  |
| # | 0.21468 |  | m+1 |  |  |  |
| # | 0.23269 |  | m+2 |  |  |  |
| # | 0.18654 | Asp 418 | m |  |  |  |
| # | 0.29574 |  | m+1 |  |  |  |
| # | 0.25279 |  | m+2 |  |  |  |
| # | 0.11151 | Glu 432 | m |  |  |  |
| # | 0.2327 |  | m+1 |  |  |  |
| # | 0.27115 |  | m+2 |  |  |  |
| # | 0.09576 | Tyr 466 | m |  |  |  |
| # | 0.18472 |  | m+1 |  |  |  |
| # | 0.19069 |  | m+2 |  |  |  |
|  |  |  |  |  |  |  |
| ## | error |  |  |  |  |  |
| # | 0.002045403 | Ala 260 | m |  |  |  |
| # | 0.001296893 |  | m+1 |  |  |  |
| # | 0.000153729 |  | m+2 |  |  |  |
| # | 0.000474383 | Ala 232 | m |  |  |  |
| # | 0.00076698 |  | m+1 |  |  |  |
| # | 0.000292596 |  | m+2 |  |  |  |
| # | 0.000846164 | Gly 246 | m |  |  |  |
| # | 0.000318919 |  | m+1 |  |  |  |
| # | 0.001020356 | Gly 218 | m |  |  |  |
| # | 0.001020356 |  | m+1 |  |  |  |
| # | 0.001127 | Val 288 | m |  |  |  |
| # | 0.000555876 |  | m+1 |  |  |  |
| # | 0.002373749 |  | m+2 |  |  |  |
| # | 0.000944599 | Val 260 | m |  |  |  |
| # | 0.000533219 |  | m+1 |  |  |  |
| # | 0.000879434 |  | m+2 |  |  |  |
| # | 0.000906468 | Leu 200 | m |  |  |  |
| # | 0.000256684 |  | m+1 |  |  |  |
| # | 5.92E-05 |  | m+2 |  |  |  |
| # | 0.000858146 | Ile 200 | m |  |  |  |
| # | 0.000769988 |  | m+1 |  |  |  |
| # | 0.00014772 |  | m+2 |  |  |  |
| # | 0.001351307 | Ser 390 | m |  |  |  |
| # | 0.00166081 |  | m+1 |  |  |  |
| # | 0.001179629 |  | m+2 |  |  |  |
| # | 0.00312952 | Ser 362 | m |  |  |  |
| # | 0.00273439 |  | m+1 |  |  |  |
| # | 0.00039513 |  | m+2 |  |  |  |
| # | 0.000878483 | Thr 404 | m |  |  |  |
| # | 0.001442389 |  | m+1 |  |  |  |
| # | 0.000652702 |  | m+2 |  |  |  |
| # | 0.00320569 | Thr 376 | m |  |  |  |
| # | 0.001714146 |  | m+1 |  |  |  |
| # | 0.001528927 |  | m+2 |  |  |  |
| # | 0.000353907 | Phe 336 | m |  |  |  |
| # | 0.001219197 |  | m+1 |  |  |  |
| # | 0.000789389 |  | m+2 |  |  |  |
| # | 0.000448852 | Phe 234 | m |  |  |  |
| # | 7.35E-05 |  | m+1 |  |  |  |
| # | 0.000685923 |  | m+2 |  |  |  |
| # | 0.002010323 | Asp 418 | m |  |  |  |
| # | 0.001911277 |  | m+1 |  |  |  |
| # | 0.002540396 |  | m+2 |  |  |  |
| # | 0.000291148 | Glu 432 | m |  |  |  |
| # | 0.000741787 |  | m+1 |  |  |  |
| # | 0.002680823 |  | m+2 |  |  |  |
| # | 0.004738689 | Tyr 466 | m |  |  |  |
| # | 0.001515612 |  | m+1 |  |  |  |
| # | 0.000169543 |  | m+2 |  |  |  |

**BL21(DE3) (pLysS) (pET-24a) on glucose and proline model**

| rxnID | rxnEQ | cTrans | rates | type | basis | deviation |
| --- | --- | --- | --- | --- | --- | --- |
| R01 | GLC_EX = GLC6P | abcdef = abcdef |  | F | 1 | 0 |
| R02 | GLC6P = F6P | abcdef = abcdef |  | FR |  |  |
| R03 | F6P=GLC6P | abcdef = abcdef |  | R | x |  |
| R04 | F6P = DHAP + G3P | abcdef = abc + def |  | F |  |  |
| R05 | DHAP = G3P | abc = cba |  | F |  |  |
| R06 | GLC6P = P5P + CO2 | abcdef = bcdef + a |  | F | X |  |
| R07 | P5P + P5P = S7P + G3P | abcde + fghij = fgabcde + hij |  | F |  |  |
| R08 | S7P + G3P = E4P + F6P | abcdefg + hij = defg + abchij |  | F |  |  |
| R09 | E4P + P5P = F6P + G3P | abcd + efghi = efabcd + ghi |  | F |  |  |
| R10 | G3P = 3PG | abc = abc |  | F |  |  |
| R11 | 3PG = PEP | abc = abc |  | FR |  |  |
| R12 | PEP = 3PG | abc = abc |  | R | X |  |
| R13 | PEP = PYR | abc = abc |  | F |  |  |
| R14 | PYR = ACCOA + CO2 | abc = bc + a |  | F |  |  |
| R15 | ACCOA + OAA = CIT | ab + cdef = fedbac |  | F |  |  |
| R16 | CIT = AKG + CO2 | abcdef = abcde + f |  | F |  |  |
| R17 | CIT = S_PREC + GLYOX | abcdef = cdef + ab |  | F | 0 |  |
| R18 | S_PREC = 0.5 SUC + 0.5 SUC | abcd = 0.5 abcd + 0.5 dcba |  | F |  |  |
| R19 | GLYOX + ACCOA = MAL | ab + cd = abdc |  | F |  |  |
| R20 | AKG = 0.5 SUC + 0.5 SUC + CO2 | abcde = 0.5 bcde + 0.5 edcb + a |  | F |  |  |
| R21 | SUC = MAL | abcd = abcd |  | FR |  |  |
| R22 | MAL = 0.5 SUC + 0.5 SUC | abcd = 0.5 abcd + 0.5 dcba |  | R | X |  |
| R23 | MAL = OAA | abcd = abcd |  | FR |  |  |
| R24 | OAA = MAL | abcd = abcd |  | R | X |  |
| R25 | MAL = PYR + CO2 | abcd = abc + d |  | F | X |  |
| R26 | PEP + CARB = OAA | abc + d = abcd |  | F | X |  |
| R27 | OAA = PEP + CO2 | abcd = abc + d |  | F |  |  |
| R28 | CARB_EX_1 = CARB | a = a |  | F | X |  |
| R29 | CARB_EX_2 = CARB | a = a |  | F |  |  |
| R30 | GLC6P = GLC6P_B |  |  | B | 0.019431 | 0.000972 |
| R31 | F6P = F6P_B |  |  | B | 0.006694 | 0.000335 |
| R32 | P5P = P5P_B |  |  | B | 0.083581 | 0.004179 |
| R33 | E4P = E4P_B |  |  | B | 0.033563 | 0.001678 |
| R34 | G3P = G3P_B |  |  | B | 0.011528 | 0.000576 |
| R35 | 3PG = 3PG_B |  |  | B | 0.150056 | 0.007503 |
| R36 | PEP = PEP_B |  |  | B | 0.072931 | 0.003647 |
| R37 | PYR = PYR_B |  |  | B | 0.275061 | 0.013753 |
| R38 | ACCOA = ACCOA_B |  |  | B | 0.226571 | 0.011329 |
| R39 | OAA = OAA_B |  |  | B | 0.151822 | 0.007591 |
| R40 | GLU = GLU_B |  |  | B | 0.092277 | 0.004614 |
| R41 | PRO = PRO_B |  |  | B | 0.027563 | 0.001378 |
| R42 | ACCOA = ACE_EX |  |  | B | X |  |
| R43 | AKG = GLU | abcde = abcde |  | FR |  |  |
| R44 | GLU = AKG | abcde = abcde |  | R | X |  |
| R45 | GLU = PRO | abcde = abcde |  | FR |  |  |
| R46 | PRO = GLU | abcde = abcde |  | R | X |  |
| R47 | PRO_EX = PRO | abcde = abcde |  | F | X |  |
| R48 | PRO + AKG = HYP + SUC_II | abcde + fghij = abcde + fghij |  | F |  |  |
| R49 | SUC_II = 0.5 SUC + 0.5 SUC + CO2 | abcde = 0.5 bcde + 0.5 edcb + a |  | F |  |  |
| R50 | HYP = HYP_EX |  |  | B | 0 |  |
| R51 | PYR + PYR = VALX + CO2X | abc + def = abefc + d |  | S |  |  |
| R52 | E4P + PEP = SHKM | abcd + efg = efgabcd |  | S |  |  |
| R53 | SHKM + PEP = CHRM | abcdefg + hij = abcdefghij |  | S |  |  |
| R54 | CHRM = PHEX + CO2X | abcdefghij = hijbcdefg + a |  | S |  |  |
| R55 | CHRM = TYRX + CO2 | abcdefghij = hijbcdefg + a |  | S |  |  |
| R56 | PYR = ALAX | abc = abc |  | S |  |  |
| R57 | OAA = THRX | abcd = abcd |  | S |  |  |
| R58 | OAA = ASPX | abcd = abcd |  | S |  |  |
| R59 | GLU = GLUX | abcde = abcde |  | S |  |  |
| R60 | PRO = PROX | abcde = abcde |  | S |  |  |
| R61 | 3PG = SERX | abc = abc |  | S |  |  |
| R62 | 3PG = GLYX + MTHF | abc = ab + c |  | S |  |  |
| R63 | PEP + OAA = ILEX + CO2X | abc + defg = debfgc + a |  | S |  |  |
| R64 | PEP + PEP = ISV + CO2X | abc + def = abefc + d |  | S |  |  |
| R65 | ISV + ACCOA = LEUX + CO2X | abcde + fg = fgbcde + a |  | S |  |  |
|  |  |  |  |  |  |  |
| ## | excludedMetabolites |  |  |  |  |  |
| # | MTHF |  |  |  |  |  |
| # | GLC_EX |  |  |  |  |  |
| # | ACE_EX |  |  |  |  |  |
| # | PRO_EX |  |  |  |  |  |
| # | CO2 |  |  |  |  |  |
| # | CARB_EX_1 |  |  |  |  |  |
| # | CARB_EX_2 |  |  |  |  |  |
| # | HYP_EX |  |  |  |  |  |
| # | GLC6P_B |  |  |  |  |  |
| # | F6P_B |  |  |  |  |  |
| # | P5P_B |  |  |  |  |  |
| # | E4P_B |  |  |  |  |  |
| # | G3P_B |  |  |  |  |  |
| # | 3PG_B |  |  |  |  |  |
| # | PEP_B |  |  |  |  |  |
| # | PYR_B |  |  |  |  |  |
| # | ACCOA_B |  |  |  |  |  |
| # | OAA_B |  |  |  |  |  |
| # | GLU_B |  |  |  |  |  |
| # | PRO_B |  |  |  |  |  |
|  |  |  |  |  |  |  |
| ## | simulatedMDVs |  |  |  |  |  |
| # | ALAX#111 |  |  |  |  |  |
| # | ALAX#011 |  |  |  |  |  |
| # | GLYX#11 |  |  |  |  |  |
| # | GLYX#01 |  |  |  |  |  |
| # | VALX#11111 |  |  |  |  |  |
| # | VALX#01111 |  |  |  |  |  |
| # | LEUX#011111 |  |  |  |  |  |
| # | ILEX#011111 |  |  |  |  |  |
| # | PROX#01111 |  |  |  |  |  |
| # | SERX#111 |  |  |  |  |  |
| # | SERX#011 |  |  |  |  |  |
| # | THRX#1111 |  |  |  |  |  |
| # | THRX#0111 |  |  |  |  |  |
| # | PHEX#111111111 |  |  |  |  |  |
| # | PHEX#011111111 |  |  |  |  |  |
| # | ASPX#1111 |  |  |  |  |  |
| # | GLUX#11111 |  |  |  |  |  |
| # | TYRX#111111111 |  |  |  |  |  |
|  |  |  |  |  |  |  |
| ## | inputSubstrates |  |  |  |  |  |
| # | CARB_EX_1 |  |  |  |  |  |
| # | CARB_EX_2 |  |  |  |  |  |
| # | GLC_EX |  |  |  |  |  |
| # | PRO_EX |  |  |  |  |  |
|  |  |  |  |  |  |  |
| ## | measurements |  |  |  |  |  |
| # | 0.376878527 | Ala 260 | m |  |  |  |
| # | 0.34305573 |  | m+1 |  |  |  |
| # | 0.118695529 |  | m+2 |  |  |  |
| # | 0.400407857 | Ala 232 | m |  |  |  |
| # | 0.36038475 |  | m+1 |  |  |  |
| # | 0.239207393 |  | m+2 |  |  |  |
| # | 0.623649074 | Gly 246 | m |  |  |  |
| # | 0.172055685 |  | m+1 |  |  |  |
| # | 0.69371476 | Gly 218 | m |  |  |  |
| # | 0.30628524 |  | m+1 |  |  |  |
| # | 0.183484874 | Val 288 | m |  |  |  |
| # | 0.282104761 |  | m+1 |  |  |  |
| # | 0.228973767 |  | m+2 |  |  |  |
| # | 0.192896665 | Val 260 | m |  |  |  |
| # | 0.290827685 |  | m+1 |  |  |  |
| # | 0.285090409 |  | m+2 |  |  |  |
| # | 0.120003795 | Leu 200 | m |  |  |  |
| # | 0.250383978 |  | m+1 |  |  |  |
| # | 0.290683708 |  | m+2 |  |  |  |
| # | 0.22549964 | Ile 200 | m |  |  |  |
| # | 0.296527638 |  | m+1 |  |  |  |
| # | 0.263182067 |  | m+2 |  |  |  |
| # | 0.818185964 | Pro 184 | m |  |  |  |
| # | 0.136946171 |  | m+1 |  |  |  |
| # | 0.038929156 |  | m+2 |  |  |  |
| # | 0.32240902 | Ser 390 | m |  |  |  |
| # | 0.348435854 |  | m+1 |  |  |  |
| # | 0.163729314 |  | m+2 |  |  |  |
| # | 0.348576506 | Ser 362 | m |  |  |  |
| # | 0.38826384 |  | m+1 |  |  |  |
| # | 0.263159654 |  | m+2 |  |  |  |
| # | 0.330799129 | Thr 404 | m |  |  |  |
| # | 0.288279377 |  | m+1 |  |  |  |
| # | 0.205860892 |  | m+2 |  |  |  |
| # | 0.369895396 | Thr 376 | m |  |  |  |
| # | 0.318426244 |  | m+1 |  |  |  |
| # | 0.213365611 |  | m+2 |  |  |  |
| # | 0.113800317 | Phe 336 | m |  |  |  |
| # | 0.197550064 |  | m+1 |  |  |  |
| # | 0.189522984 |  | m+2 |  |  |  |
| # | 0.129908311 | Phe 234 | m |  |  |  |
| # | 0.216086859 |  | m+1 |  |  |  |
| # | 0.231872827 |  | m+2 |  |  |  |
| # | 0.335813938 | Asp 418 | m |  |  |  |
| # | 0.286920627 |  | m+1 |  |  |  |
| # | 0.205344935 |  | m+2 |  |  |  |
| # | 0.333439536 | Glu 432 | m |  |  |  |
| # | 0.255808727 |  | m+1 |  |  |  |
| # | 0.207877597 |  | m+2 |  |  |  |
| # | 0.099530147 | Tyr 466 | m |  |  |  |
| # | 0.183664342 |  | m+1 |  |  |  |
| # | 0.190413899 |  | m+2 |  |  |  |
|  |  |  |  |  |  |  |
| ## | error |  |  |  |  |  |
| # | 0.00050081 | Ala 260 | m |  |  |  |
| # | 0.0007235 |  | m+1 |  |  |  |
| # | 0.0009547 |  | m+2 |  |  |  |
| # | 0.00085204 | Ala 232 | m |  |  |  |
| # | 0.00086145 |  | m+1 |  |  |  |
| # | 0.00000941 |  | m+2 |  |  |  |
| # | 0.00025369 | Gly 246 | m |  |  |  |
| # | 0.00106448 |  | m+1 |  |  |  |
| # | 0.00028471 | Gly 218 | m |  |  |  |
| # | 0.00028471 |  | m+1 |  |  |  |
| # | 0.00008009 | Val 288 | m |  |  |  |
| # | 0.00058088 |  | m+1 |  |  |  |
| # | 0.00054355 |  | m+2 |  |  |  |
| # | 0.00003347 | Val 260 | m |  |  |  |
| # | 0.00048433 |  | m+1 |  |  |  |
| # | 0.00075207 |  | m+2 |  |  |  |
| # | 0.00035968 | Leu 200 | m |  |  |  |
| # | 0.0006284 |  | m+1 |  |  |  |
| # | 0.0000446 |  | m+2 |  |  |  |
| # | 0.00000035 | Ile 200 | m |  |  |  |
| # | 0.00064459 |  | m+1 |  |  |  |
| # | 0.00071291 |  | m+2 |  |  |  |
| # | 0.00430733 | Pro 184 | m |  |  |  |
| # | 0.00129832 |  | m+1 |  |  |  |
| # | 0.00152657 |  | m+2 |  |  |  |
| # | 0.00117276 | Ser 390 | m |  |  |  |
| # | 0.00042295 |  | m+1 |  |  |  |
| # | 0.00000377 |  | m+2 |  |  |  |
| # | 0.00279622 | Ser 362 | m |  |  |  |
| # | 0.00053238 |  | m+1 |  |  |  |
| # | 0.00226384 |  | m+2 |  |  |  |
| # | 0.00022018 | Thr 404 | m |  |  |  |
| # | 0.0003736 |  | m+1 |  |  |  |
| # | 0.00063523 |  | m+2 |  |  |  |
| # | 0.00206174 | Thr 376 | m |  |  |  |
| # | 0.00050751 |  | m+1 |  |  |  |
| # | 0.00138009 |  | m+2 |  |  |  |
| # | 0.00164425 | Phe 336 | m |  |  |  |
| # | 0.00116697 |  | m+1 |  |  |  |
| # | 0.0003525 |  | m+2 |  |  |  |
| # | 0.00020577 | Phe 234 | m |  |  |  |
| # | 0.00038583 |  | m+1 |  |  |  |
| # | 0.00006423 |  | m+2 |  |  |  |
| # | 0.00054942 | Asp 418 | m |  |  |  |
| # | 0.00013117 |  | m+1 |  |  |  |
| # | 0.000275 |  | m+2 |  |  |  |
| # | 0.00000814 | Glu 432 | m |  |  |  |
| # | 0.00054352 |  | m+1 |  |  |  |
| # | 0.00025811 |  | m+2 |  |  |  |
| # | 0.00103453 | Tyr 466 | m |  |  |  |
| # | 0.00084897 |  | m+1 |  |  |  |
| # | 0.00128171 |  | m+2 |  |  |  |

**BL21 *ΔputA* (DE3) (pLysS) (pET-24a) on glucose and proline model**

| rxnID | rxnEQ | cTrans | rates | type | basis | deviation |
| --- | --- | --- | --- | --- | --- | --- |
| R01 | GLC_EX = GLC6P | abcdef = abcdef |  | F | 1 | 0 |
| R02 | GLC6P = F6P | abcdef = abcdef |  | FR |  |  |
| R03 | F6P=GLC6P | abcdef = abcdef |  | R | x |  |
| R04 | F6P = DHAP + G3P | abcdef = abc + def |  | F |  |  |
| R05 | DHAP = G3P | abc = cba |  | F |  |  |
| R06 | GLC6P = P5P + CO2 | abcdef = bcdef + a |  | F | X |  |
| R07 | P5P + P5P = S7P + G3P | abcde + fghij = fgabcde + hij |  | F |  |  |
| R08 | S7P + G3P = E4P + F6P | abcdefg + hij = defg + abchij |  | F |  |  |
| R09 | E4P + P5P = F6P + G3P | abcd + efghi = efabcd + ghi |  | F |  |  |
| R10 | G3P = 3PG | abc = abc |  | F |  |  |
| R11 | 3PG = PEP | abc = abc |  | FR |  |  |
| R12 | PEP = 3PG | abc = abc |  | R | X |  |
| R13 | PEP = PYR | abc = abc |  | F |  |  |
| R14 | PYR = ACCOA + CO2 | abc = bc + a |  | F |  |  |
| R15 | ACCOA + OAA = CIT | ab + cdef = fedbac |  | F |  |  |
| R16 | CIT = AKG + CO2 | abcdef = abcde + f |  | F |  |  |
| R17 | CIT = S_PREC + GLYOX | abcdef = cdef + ab |  | F | 0 |  |
| R18 | S_PREC = 0.5 SUC + 0.5 SUC | abcd = 0.5 abcd + 0.5 dcba |  | F |  |  |
| R19 | GLYOX + ACCOA = MAL | ab + cd = abdc |  | F |  |  |
| R20 | AKG = 0.5 SUC + 0.5 SUC + CO2 | abcde = 0.5 bcde + 0.5 edcb + a |  | F |  |  |
| R21 | SUC = MAL | abcd = abcd |  | FR |  |  |
| R22 | MAL = 0.5 SUC + 0.5 SUC | abcd = 0.5 abcd + 0.5 dcba |  | R | X |  |
| R23 | MAL = OAA | abcd = abcd |  | FR |  |  |
| R24 | OAA = MAL | abcd = abcd |  | R | X |  |
| R25 | MAL = PYR + CO2 | abcd = abc + d |  | F | X |  |
| R26 | PEP + CARB = OAA | abc + d = abcd |  | F | X |  |
| R27 | OAA = PEP + CO2 | abcd = abc + d |  | F |  |  |
| R28 | CARB_EX_1 = CARB | a = a |  | F | X |  |
| R29 | CARB_EX_2 = CARB | a = a |  | F |  |  |
| R30 | GLC6P = GLC6P_B |  |  | B | 0.018261 | 0.000913 |
| R31 | F6P = F6P_B |  |  | B | 0.006291 | 0.000315 |
| R32 | P5P = P5P_B |  |  | B | 0.078547 | 0.003927 |
| R33 | E4P = E4P_B |  |  | B | 0.031541 | 0.001577 |
| R34 | G3P = G3P_B |  |  | B | 0.010834 | 0.000542 |
| R35 | 3PG = 3PG_B |  |  | B | 0.141018 | 0.007051 |
| R36 | PEP = PEP_B |  |  | B | 0.068539 | 0.003427 |
| R37 | PYR = PYR_B |  |  | B | 0.258493 | 0.012925 |
| R38 | ACCOA = ACCOA_B |  |  | B | 0.212924 | 0.010646 |
| R39 | OAA = OAA_B |  |  | B | 0.142678 | 0.007134 |
| R40 | GLU = GLU_B |  |  | B | 0.086719 | 0.004336 |
| R41 | PRO = PRO_B |  |  | B | 0.025903 | 0.001295 |
| R42 | ACCOA = ACE_EX |  |  | B | X |  |
| R43 | AKG = GLU | abcde = abcde |  | FR |  |  |
| R44 | GLU = AKG | abcde = abcde |  | R | X |  |
| R45 | GLU = PRO | abcde = abcde |  | FR |  |  |
| R46 | PRO = GLU | abcde = abcde |  | R | X |  |
| R47 | PRO_EX = PRO | abcde = abcde |  | F | X |  |
| R48 | PRO + AKG = HYP + SUC_II | abcde + fghij = abcde + fghij |  | F |  |  |
| R49 | SUC_II = 0.5 SUC + 0.5 SUC + CO2 | abcde = 0.5 bcde + 0.5 edcb + a |  | F |  |  |
| R50 | HYP = HYP_EX |  |  | B | 0 |  |
| R51 | PYR + PYR = VALX + CO2X | abc + def = abefc + d |  | S |  |  |
| R52 | E4P + PEP = SHKM | abcd + efg = efgabcd |  | S |  |  |
| R53 | SHKM + PEP = CHRM | abcdefg + hij = abcdefghij |  | S |  |  |
| R54 | CHRM = PHEX + CO2X | abcdefghij = hijbcdefg + a |  | S |  |  |
| R55 | CHRM = TYRX + CO2 | abcdefghij = hijbcdefg + a |  | S |  |  |
| R56 | PYR = ALAX | abc = abc |  | S |  |  |
| R57 | OAA = THRX | abcd = abcd |  | S |  |  |
| R58 | OAA = ASPX | abcd = abcd |  | S |  |  |
| R59 | GLU = GLUX | abcde = abcde |  | S |  |  |
| R60 | PRO = PROX | abcde = abcde |  | S |  |  |
| R61 | 3PG = SERX | abc = abc |  | S |  |  |
| R62 | 3PG = GLYX + MTHF | abc = ab + c |  | S |  |  |
| R63 | PEP + OAA = ILEX + CO2X | abc + defg = debfgc + a |  | S |  |  |
| R64 | PEP + PEP = ISV + CO2X | abc + def = abefc + d |  | S |  |  |
| R65 | ISV + ACCOA = LEUX + CO2X | abcde + fg = fgbcde + a |  | S |  |  |
|  |  |  |  |  |  |  |
| ## | excludedMetabolites |  |  |  |  |  |
| # | MTHF |  |  |  |  |  |
| # | GLC_EX |  |  |  |  |  |
| # | ACE_EX |  |  |  |  |  |
| # | PRO_EX |  |  |  |  |  |
| # | CO2 |  |  |  |  |  |
| # | CARB_EX_1 |  |  |  |  |  |
| # | CARB_EX_2 |  |  |  |  |  |
| # | HYP_EX |  |  |  |  |  |
| # | GLC6P_B |  |  |  |  |  |
| # | F6P_B |  |  |  |  |  |
| # | P5P_B |  |  |  |  |  |
| # | E4P_B |  |  |  |  |  |
| # | G3P_B |  |  |  |  |  |
| # | 3PG_B |  |  |  |  |  |
| # | PEP_B |  |  |  |  |  |
| # | PYR_B |  |  |  |  |  |
| # | ACCOA_B |  |  |  |  |  |
| # | OAA_B |  |  |  |  |  |
| # | GLU_B |  |  |  |  |  |
| # | PRO_B |  |  |  |  |  |
|  |  |  |  |  |  |  |
| ## | simulatedMDVs |  |  |  |  |  |
| # | ALAX#111 |  |  |  |  |  |
| # | ALAX#011 |  |  |  |  |  |
| # | GLYX#11 |  |  |  |  |  |
| # | GLYX#01 |  |  |  |  |  |
| # | VALX#11111 |  |  |  |  |  |
| # | VALX#01111 |  |  |  |  |  |
| # | LEUX#011111 |  |  |  |  |  |
| # | ILEX#011111 |  |  |  |  |  |
| # | PROX#01111 |  |  |  |  |  |
| # | SERX#111 |  |  |  |  |  |
| # | SERX#011 |  |  |  |  |  |
| # | THRX#1111 |  |  |  |  |  |
| # | THRX#0111 |  |  |  |  |  |
| # | PHEX#111111111 |  |  |  |  |  |
| # | PHEX#011111111 |  |  |  |  |  |
| # | ASPX#1111 |  |  |  |  |  |
| # | GLUX#11111 |  |  |  |  |  |
| # | TYRX#111111111 |  |  |  |  |  |
|  |  |  |  |  |  |  |
| ## | inputSubstrates |  |  |  |  |  |
| # | CARB_EX_1 |  |  |  |  |  |
| # | CARB_EX_2 |  |  |  |  |  |
| # | GLC_EX |  |  |  |  |  |
| # | PRO_EX |  |  |  |  |  |
|  |  |  |  |  |  |  |
| ## | measurements |  |  |  |  |  |
| # | 0.36774 | Ala 260 | m |  |  |  |
| # | 0.34711 |  | m+1 |  |  |  |
| # | 0.11845 |  | m+2 |  |  |  |
| # | 0.39021 | Ala 232 | m |  |  |  |
| # | 0.36452 |  | m+1 |  |  |  |
| # | 0.24527 |  | m+2 |  |  |  |
| # | 0.62226 | Gly 246 | m |  |  |  |
| # | 0.17314 |  | m+1 |  |  |  |
| # | 0.69113 | Gly 218 | m |  |  |  |
| # | 0.30887 |  | m+1 |  |  |  |
| # | 0.17495 | Val 288 | m |  |  |  |
| # | 0.27786 |  | m+1 |  |  |  |
| # | 0.23081 |  | m+2 |  |  |  |
| # | 0.1834 | Val 260 | m |  |  |  |
| # | 0.28619 |  | m+1 |  |  |  |
| # | 0.28989 |  | m+2 |  |  |  |
| # | 0.1137 | Leu 200 | m |  |  |  |
| # | 0.2412 |  | m+1 |  |  |  |
| # | 0.29125 |  | m+2 |  |  |  |
| # | 0.13979 | Ile 200 | m |  |  |  |
| # | 0.25951 |  | m+1 |  |  |  |
| # | 0.28589 |  | m+2 |  |  |  |
| # | 0.81945 | Pro 184 | m |  |  |  |
| # | 0.13569 |  | m+1 |  |  |  |
| # | 0.03871 |  | m+2 |  |  |  |
| # | 0.32075 | Ser 390 | m |  |  |  |
| # | 0.34852 |  | m+1 |  |  |  |
| # | 0.16618 |  | m+2 |  |  |  |
| # | 0.34644 | Ser 362 | m |  |  |  |
| # | 0.39057 |  | m+1 |  |  |  |
| # | 0.26299 |  | m+2 |  |  |  |
| # | 0.19015 | Thr 404 | m |  |  |  |
| # | 0.29636 |  | m+1 |  |  |  |
| # | 0.24698 |  | m+2 |  |  |  |
| # | 0.23247 | Thr 376 | m |  |  |  |
| # | 0.34547 |  | m+1 |  |  |  |
| # | 0.27686 |  | m+2 |  |  |  |
| # | 0.11023 | Phe 336 | m |  |  |  |
| # | 0.19532 |  | m+1 |  |  |  |
| # | 0.19098 |  | m+2 |  |  |  |
| # | 0.12572 | Phe 234 | m |  |  |  |
| # | 0.21258 |  | m+1 |  |  |  |
| # | 0.23216 |  | m+2 |  |  |  |
| # | 0.19401 | Asp 418 | m |  |  |  |
| # | 0.29533 |  | m+1 |  |  |  |
| # | 0.24878 |  | m+2 |  |  |  |
| # | 0.1151 | Glu 432 | m |  |  |  |
| # | 0.23229 |  | m+1 |  |  |  |
| # | 0.26735 |  | m+2 |  |  |  |
| # | 0.09408 | Tyr 466 | m |  |  |  |
| # | 0.18276 |  | m+1 |  |  |  |
| # | 0.19151 |  | m+2 |  |  |  |
|  |  |  |  |  |  |  |
| ## | error |  |  |  |  |  |
| # | 0.00113 | Ala 260 | m |  |  |  |
| # | 0.00025 |  | m+1 |  |  |  |
| # | 0.00028 |  | m+2 |  |  |  |
| # | 0.0007 | Ala 232 | m |  |  |  |
| # | 0.00018 |  | m+1 |  |  |  |
| # | 0.00052 |  | m+2 |  |  |  |
| # | 0.00015 | Gly 246 | m |  |  |  |
| # | 0.00107 |  | m+1 |  |  |  |
| # | 0.00026 | Gly 218 | m |  |  |  |
| # | 0.00026 |  | m+1 |  |  |  |
| # | 0.00104 | Val 288 | m |  |  |  |
| # | 0.00111 |  | m+1 |  |  |  |
| # | 0.00246 |  | m+2 |  |  |  |
| # | 0.00156 | Val 260 | m |  |  |  |
| # | 0.00054 |  | m+1 |  |  |  |
| # | 0.00074 |  | m+2 |  |  |  |
| # | 0.00025 | Leu 200 | m |  |  |  |
| # | 0.00002 |  | m+1 |  |  |  |
| # | 0.00038 |  | m+2 |  |  |  |
| # | 0.00089 | Ile 200 | m |  |  |  |
| # | 0.0004 |  | m+1 |  |  |  |
| # | 0.00024 |  | m+2 |  |  |  |
| # | 0.00452 | Pro 184 | m |  |  |  |
| # | 0.00221 |  | m+1 |  |  |  |
| # | 0.00129 |  | m+2 |  |  |  |
| # | 0.00067 | Ser 390 | m |  |  |  |
| # | 0.00029 |  | m+1 |  |  |  |
| # | 0.00038 |  | m+2 |  |  |  |
| # | 0.00145 | Ser 362 | m |  |  |  |
| # | 0.0008 |  | m+1 |  |  |  |
| # | 0.00065 |  | m+2 |  |  |  |
| # | 0.00306 | Thr 404 | m |  |  |  |
| # | 0.0028 |  | m+1 |  |  |  |
| # | 0.00169 |  | m+2 |  |  |  |
| # | 0.00108 | Thr 376 | m |  |  |  |
| # | 0.00054 |  | m+1 |  |  |  |
| # | 0.00026 |  | m+2 |  |  |  |
| # | 0.00126 | Phe 336 | m |  |  |  |
| # | 0.00231 |  | m+1 |  |  |  |
| # | 0.00097 |  | m+2 |  |  |  |
| # | 0.00031 | Phe 234 | m |  |  |  |
| # | 0.00008 |  | m+1 |  |  |  |
| # | 0.00082 |  | m+2 |  |  |  |
| # | 0.00085 | Asp 418 | m |  |  |  |
| # | 0.00062 |  | m+1 |  |  |  |
| # | 0.00096 |  | m+2 |  |  |  |
| # | 0.00176 | Glu 432 | m |  |  |  |
| # | 0.00045 |  | m+1 |  |  |  |
| # | 0.00212 |  | m+2 |  |  |  |
| # | 0.00117 | Tyr 466 | m |  |  |  |
| # | 0.0019 |  | m+1 |  |  |  |
| # | 0.00032 |  | m+2 |  |  |  |

**BL21(DE3) (pLysS) (pET-p4h1of) on glucose and proline model**

| rxnID | rxnEQ | cTrans | rates | type | basis | deviation |
| --- | --- | --- | --- | --- | --- | --- |
| R01 | GLC_EX = GLC6P | abcdef = abcdef |  | F | 1 | 0 |
| R02 | GLC6P = F6P | abcdef = abcdef |  | FR |  |  |
| R03 | F6P=GLC6P | abcdef = abcdef |  | R | x |  |
| R04 | F6P = DHAP + G3P | abcdef = abc + def |  | F |  |  |
| R05 | DHAP = G3P | abc = cba |  | F |  |  |
| R06 | GLC6P = P5P + CO2 | abcdef = bcdef + a |  | F | X |  |
| R07 | P5P + P5P = S7P + G3P | abcde + fghij = fgabcde + hij |  | F |  |  |
| R08 | S7P + G3P = E4P + F6P | abcdefg + hij = defg + abchij |  | F |  |  |
| R09 | E4P + P5P = F6P + G3P | abcd + efghi = efabcd + ghi |  | F |  |  |
| R10 | G3P = 3PG | abc = abc |  | F |  |  |
| R11 | 3PG = PEP | abc = abc |  | FR |  |  |
| R12 | PEP = 3PG | abc = abc |  | R | X |  |
| R13 | PEP = PYR | abc = abc |  | F |  |  |
| R14 | PYR = ACCOA + CO2 | abc = bc + a |  | F |  |  |
| R15 | ACCOA + OAA = CIT | ab + cdef = fedbac |  | F |  |  |
| R16 | CIT = AKG + CO2 | abcdef = abcde + f |  | F |  |  |
| R17 | CIT = S_PREC + GLYOX | abcdef = cdef + ab |  | F | 0 |  |
| R18 | S_PREC = 0.5 SUC + 0.5 SUC | abcd = 0.5 abcd + 0.5 dcba |  | F |  |  |
| R19 | GLYOX + ACCOA = MAL | ab + cd = abdc |  | F |  |  |
| R20 | AKG = 0.5 SUC + 0.5 SUC + CO2 | abcde = 0.5 bcde + 0.5 edcb + a |  | F |  |  |
| R21 | SUC = MAL | abcd = abcd |  | FR |  |  |
| R22 | MAL = 0.5 SUC + 0.5 SUC | abcd = 0.5 abcd + 0.5 dcba |  | R | X |  |
| R23 | MAL = OAA | abcd = abcd |  | FR |  |  |
| R24 | OAA = MAL | abcd = abcd |  | R | X |  |
| R25 | MAL = PYR + CO2 | abcd = abc + d |  | F | X |  |
| R26 | PEP + CARB = OAA | abc + d = abcd |  | F | X |  |
| R27 | OAA = PEP + CO2 | abcd = abc + d |  | F |  |  |
| R28 | CARB_EX_1 = CARB | a = a |  | F | X |  |
| R29 | CARB_EX_2 = CARB | a = a |  | F |  |  |
| R30 | GLC6P = GLC6P_B |  |  | B | 0.015929 | 0.000796 |
| R31 | F6P = F6P_B |  |  | B | 0.005487 | 0.000274 |
| R32 | P5P = P5P_B |  |  | B | 0.068516 | 0.003426 |
| R33 | E4P = E4P_B |  |  | B | 0.027513 | 0.001376 |
| R34 | G3P = G3P_B |  |  | B | 0.00945 | 0.000473 |
| R35 | 3PG = 3PG_B |  |  | B | 0.123009 | 0.00615 |
| R36 | PEP = PEP_B |  |  | B | 0.059786 | 0.002989 |
| R37 | PYR = PYR_B |  |  | B | 0.225482 | 0.011274 |
| R38 | ACCOA = ACCOA_B |  |  | B | 0.185733 | 0.009287 |
| R39 | OAA = OAA_B |  |  | B | 0.124457 | 0.006223 |
| R40 | GLU = GLU_B |  |  | B | 0.075644 | 0.003782 |
| R41 | PRO = PRO_B |  |  | B | 0.022595 | 0.00113 |
| R42 | ACCOA = ACE_EX |  |  | B | X |  |
| R43 | AKG = GLU | abcde = abcde |  | FR |  |  |
| R44 | GLU = AKG | abcde = abcde |  | R | X |  |
| R45 | GLU = PRO | abcde = abcde |  | FR |  |  |
| R46 | PRO = GLU | abcde = abcde |  | R | X |  |
| R47 | PRO_EX = PRO | abcde = abcde |  | F | X |  |
| R48 | PRO + AKG = HYP + SUC_II | abcde + fghij = abcde + fghij |  | F |  |  |
| R49 | SUC_II = 0.5 SUC + 0.5 SUC + CO2 | abcde = 0.5 bcde + 0.5 edcb + a |  | F |  |  |
| R50 | HYP = HYP_EX |  |  | B | X |  |
| R51 | PYR + PYR = VALX + CO2X | abc + def = abefc + d |  | S |  |  |
| R52 | E4P + PEP = SHKM | abcd + efg = efgabcd |  | S |  |  |
| R53 | SHKM + PEP = CHRM | abcdefg + hij = abcdefghij |  | S |  |  |
| R54 | CHRM = PHEX + CO2X | abcdefghij = hijbcdefg + a |  | S |  |  |
| R55 | CHRM = TYRX + CO2 | abcdefghij = hijbcdefg + a |  | S |  |  |
| R56 | PYR = ALAX | abc = abc |  | S |  |  |
| R57 | OAA = THRX | abcd = abcd |  | S |  |  |
| R58 | OAA = ASPX | abcd = abcd |  | S |  |  |
| R59 | GLU = GLUX | abcde = abcde |  | S |  |  |
| R60 | PRO = PROX | abcde = abcde |  | S |  |  |
| R61 | 3PG = SERX | abc = abc |  | S |  |  |
| R62 | 3PG = GLYX + MTHF | abc = ab + c |  | S |  |  |
| R63 | PEP + OAA = ILEX + CO2X | abc + defg = debfgc + a |  | S |  |  |
| R64 | PEP + PEP = ISV + CO2X | abc + def = abefc + d |  | S |  |  |
| R65 | ISV + ACCOA = LEUX + CO2X | abcde + fg = fgbcde + a |  | S |  |  |
|  |  |  |  |  |  |  |
| ## | excludedMetabolites |  |  |  |  |  |
| # | MTHF |  |  |  |  |  |
| # | GLC_EX |  |  |  |  |  |
| # | ACE_EX |  |  |  |  |  |
| # | PRO_EX |  |  |  |  |  |
| # | CO2 |  |  |  |  |  |
| # | CARB_EX_1 |  |  |  |  |  |
| # | CARB_EX_2 |  |  |  |  |  |
| # | HYP_EX |  |  |  |  |  |
| # | GLC6P_B |  |  |  |  |  |
| # | F6P_B |  |  |  |  |  |
| # | P5P_B |  |  |  |  |  |
| # | E4P_B |  |  |  |  |  |
| # | G3P_B |  |  |  |  |  |
| # | 3PG_B |  |  |  |  |  |
| # | PEP_B |  |  |  |  |  |
| # | PYR_B |  |  |  |  |  |
| # | ACCOA_B |  |  |  |  |  |
| # | OAA_B |  |  |  |  |  |
| # | GLU_B |  |  |  |  |  |
| # | PRO_B |  |  |  |  |  |
|  |  |  |  |  |  |  |
| ## | simulatedMDVs |  |  |  |  |  |
| # | ALAX#111 |  |  |  |  |  |
| # | ALAX#011 |  |  |  |  |  |
| # | GLYX#11 |  |  |  |  |  |
| # | GLYX#01 |  |  |  |  |  |
| # | VALX#11111 |  |  |  |  |  |
| # | VALX#01111 |  |  |  |  |  |
| # | LEUX#011111 |  |  |  |  |  |
| # | ILEX#011111 |  |  |  |  |  |
| # | PROX#01111 |  |  |  |  |  |
| # | SERX#111 |  |  |  |  |  |
| # | SERX#011 |  |  |  |  |  |
| # | THRX#1111 |  |  |  |  |  |
| # | THRX#0111 |  |  |  |  |  |
| # | PHEX#111111111 |  |  |  |  |  |
| # | PHEX#011111111 |  |  |  |  |  |
| # | ASPX#1111 |  |  |  |  |  |
| # | GLUX#11111 |  |  |  |  |  |
| # | TYRX#111111111 |  |  |  |  |  |
|  |  |  |  |  |  |  |
| ## | inputSubstrates |  |  |  |  |  |
| # | CARB_EX_1 |  |  |  |  |  |
| # | CARB_EX_2 |  |  |  |  |  |
| # | GLC_EX |  |  |  |  |  |
| # | PRO_EX |  |  |  |  |  |
|  |  |  |  |  |  |  |
| ## | measurements |  |  |  |  |  |
| # | 0.35954 | Ala 260 | m |  |  |  |
| # | 0.34921 |  | m+1 |  |  |  |
| # | 0.12479 |  | m+2 |  |  |  |
| # | 0.38133 | Ala 232 | m |  |  |  |
| # | 0.37223 |  | m+1 |  |  |  |
| # | 0.24644 |  | m+2 |  |  |  |
| # | 0.62076 | Gly 246 | m |  |  |  |
| # | 0.1749 |  | m+1 |  |  |  |
| # | 0.69213 | Gly 218 | m |  |  |  |
| # | 0.30787 |  | m+1 |  |  |  |
| # | 0.16935 | Val 288 | m |  |  |  |
| # | 0.27752 |  | m+1 |  |  |  |
| # | 0.23492 |  | m+2 |  |  |  |
| # | 0.17539 | Val 260 | m |  |  |  |
| # | 0.28791 |  | m+1 |  |  |  |
| # | 0.29091 |  | m+2 |  |  |  |
| # | 0.10538 | Leu 200 | m |  |  |  |
| # | 0.23464 |  | m+1 |  |  |  |
| # | 0.29411 |  | m+2 |  |  |  |
| # | 0.15677 | Ile 200 | m |  |  |  |
| # | 0.26781 |  | m+1 |  |  |  |
| # | 0.28286 |  | m+2 |  |  |  |
| # | 0.819 | Pro 184 | m |  |  |  |
| # | 0.13663 |  | m+1 |  |  |  |
| # | 0.03858 |  | m+2 |  |  |  |
| # | 0.31102 | Ser 390 | m |  |  |  |
| # | 0.35336 |  | m+1 |  |  |  |
| # | 0.16958 |  | m+2 |  |  |  |
| # | 0.33773 | Ser 362 | m |  |  |  |
| # | 0.39703 |  | m+1 |  |  |  |
| # | 0.26524 |  | m+2 |  |  |  |
| # | 0.23157 | Thr 404 | m |  |  |  |
| # | 0.28834 |  | m+1 |  |  |  |
| # | 0.23987 |  | m+2 |  |  |  |
| # | 0.27259 | Thr 376 | m |  |  |  |
| # | 0.33411 |  | m+1 |  |  |  |
| # | 0.26 |  | m+2 |  |  |  |
| # | 0.10918 | Phe 336 | m |  |  |  |
| # | 0.19706 |  | m+1 |  |  |  |
| # | 0.19219 |  | m+2 |  |  |  |
| # | 0.1249 | Phe 234 | m |  |  |  |
| # | 0.21533 |  | m+1 |  |  |  |
| # | 0.23375 |  | m+2 |  |  |  |
| # | 0.23251 | Asp 418 | m |  |  |  |
| # | 0.28767 |  | m+1 |  |  |  |
| # | 0.24089 |  | m+2 |  |  |  |
| # | 0.19528 | Glu 432 | m |  |  |  |
| # | 0.23614 |  | m+1 |  |  |  |
| # | 0.24843 |  | m+2 |  |  |  |
| # | 0.09558 | Tyr 466 | m |  |  |  |
| # | 0.1835 |  | m+1 |  |  |  |
| # | 0.19307 |  | m+2 |  |  |  |
|  |  |  |  |  |  |  |
| ## | error |  |  |  |  |  |
| # | 0.00001 | Ala 260 | m |  |  |  |
| # | 0.00103 |  | m+1 |  |  |  |
| # | 0.0001 |  | m+2 |  |  |  |
| # | 0.00127 | Ala 232 | m |  |  |  |
| # | 0.00057 |  | m+1 |  |  |  |
| # | 0.0007 |  | m+2 |  |  |  |
| # | 0.00005 | Gly 246 | m |  |  |  |
| # | 0.00058 |  | m+1 |  |  |  |
| # | 0.00016 | Gly 218 | m |  |  |  |
| # | 0.00016 |  | m+1 |  |  |  |
| # | 0.00018 | Val 288 | m |  |  |  |
| # | 0.00223 |  | m+1 |  |  |  |
| # | 0.00115 |  | m+2 |  |  |  |
| # | 0.00018 | Val 260 | m |  |  |  |
| # | 0.00187 |  | m+1 |  |  |  |
| # | 0.00181 |  | m+2 |  |  |  |
| # | 0.00019 | Leu 200 | m |  |  |  |
| # | 0.00077 |  | m+1 |  |  |  |
| # | 0.00079 |  | m+2 |  |  |  |
| # | 0.00201 | Ile 200 | m |  |  |  |
| # | 0.00082 |  | m+1 |  |  |  |
| # | 0.00039 |  | m+2 |  |  |  |
| # | 0.00218 | Pro 184 | m |  |  |  |
| # | 0.00087 |  | m+1 |  |  |  |
| # | 0.00064 |  | m+2 |  |  |  |
| # | 0.00036 | Ser 390 | m |  |  |  |
| # | 0.0006 |  | m+1 |  |  |  |
| # | 0.00057 |  | m+2 |  |  |  |
| # | 0.00466 | Ser 362 | m |  |  |  |
| # | 0.0031 |  | m+1 |  |  |  |
| # | 0.00156 |  | m+2 |  |  |  |
| # | 0.00015 | Thr 404 | m |  |  |  |
| # | 0.00252 |  | m+1 |  |  |  |
| # | 0.00025 |  | m+2 |  |  |  |
| # | 0.00358 | Thr 376 | m |  |  |  |
| # | 0.00057 |  | m+1 |  |  |  |
| # | 0.00341 |  | m+2 |  |  |  |
| # | 0.00261 | Phe 336 | m |  |  |  |
| # | 0.00095 |  | m+1 |  |  |  |
| # | 0.00119 |  | m+2 |  |  |  |
| # | 0.00019 | Phe 234 | m |  |  |  |
| # | 0.0003 |  | m+1 |  |  |  |
| # | 0.00067 |  | m+2 |  |  |  |
| # | 0.0005 | Asp 418 | m |  |  |  |
| # | 0.00077 |  | m+1 |  |  |  |
| # | 0.00064 |  | m+2 |  |  |  |
| # | 0.00036 | Glu 432 | m |  |  |  |
| # | 0.00058 |  | m+1 |  |  |  |
| # | 0.00081 |  | m+2 |  |  |  |
| # | 0.00276 | Tyr 466 | m |  |  |  |
| # | 0.00033 |  | m+1 |  |  |  |
| # | 0.00063 |  | m+2 |  |  |  |

**BL21 *ΔputA* (DE3) (pLysS) (pET-p4h1of) on glucose and proline model**

| rxnID | rxnEQ | cTrans | rates | type | basis | deviation |  |  |
| --- | --- | --- | --- | --- | --- | --- | --- | --- |
| R01 | GLC_EX = GLC6P | abcdef = abcdef |  | F | 1 | 0 |  |  |
| R02 | GLC6P = F6P | abcdef = abcdef |  | FR |  |  |  |  |
| R03 | F6P=GLC6P | abcdef = abcdef |  | R | x |  |  |  |
| R04 | F6P = DHAP + G3P | abcdef = abc + def |  | F |  |  |  |  |
| R05 | DHAP = G3P | abc = cba |  | F |  |  |  |  |
| R06 | GLC6P = P5P + CO2 | abcdef = bcdef + a |  | F | X |  |  |  |
| R07 | P5P + P5P = S7P + G3P | abcde + fghij = fgabcde + hij |  | F |  |  |  |  |
| R08 | S7P + G3P = E4P + F6P | abcdefg + hij = defg + abchij |  | F |  |  |  |  |
| R09 | E4P + P5P = F6P + G3P | abcd + efghi = efabcd + ghi |  | F |  |  |  |  |
| R10 | G3P = 3PG | abc = abc |  | F |  |  |  |  |
| R11 | 3PG = PEP | abc = abc |  | FR |  |  |  |  |
| R12 | PEP = 3PG | abc = abc |  | R | X |  |  |  |
| R13 | PEP = PYR | abc = abc |  | F |  |  |  |  |
| R14 | PYR = ACCOA + CO2 | abc = bc + a |  | F |  |  |  |  |
| R15 | ACCOA + OAA = CIT | ab + cdef = fedbac |  | F |  |  |  |  |
| R16 | CIT = AKG + CO2 | abcdef = abcde + f |  | F |  |  |  |  |
| R17 | CIT = S_PREC + GLYOX | abcdef = cdef + ab |  | F | 0 |  |  |  |
| R18 | S_PREC = 0.5 SUC + 0.5 SUC | abcd = 0.5 abcd + 0.5 dcba |  | F |  |  |  |  |
| R19 | GLYOX + ACCOA = MAL | ab + cd = abdc |  | F |  |  |  |  |
| R20 | AKG = 0.5 SUC + 0.5 SUC + CO2 | abcde = 0.5 bcde + 0.5 edcb + a |  | F |  |  |  |  |
| R21 | SUC = MAL | abcd = abcd |  | FR |  |  |  |  |
| R22 | MAL = 0.5 SUC + 0.5 SUC | abcd = 0.5 abcd + 0.5 dcba |  | R | X |  |  |  |
| R23 | MAL = OAA | abcd = abcd |  | FR |  |  |  |  |
| R24 | OAA = MAL | abcd = abcd |  | R | X |  |  |  |
| R25 | MAL = PYR + CO2 | abcd = abc + d |  | F | X |  |  |  |
| R26 | PEP + CARB = OAA | abc + d = abcd |  | F | X |  |  |  |
| R27 | OAA = PEP + CO2 | abcd = abc + d |  | F |  |  |  |  |
| R28 | CARB_EX_1 = CARB | a = a |  | F | X |  |  |  |
| R29 | CARB_EX_2 = CARB | a = a |  | F |  |  |  |  |
| R30 | GLC6P = GLC6P_B |  |  | B | 0.016922 | 0.000846 |  |  |
| R31 | F6P = F6P_B |  |  | B | 0.005829 | 0.000291 |  |  |
| R32 | P5P = P5P_B |  |  | B | 0.072787 | 0.003639 |  |  |
| R33 | E4P = E4P_B |  |  | B | 0.029228 | 0.001461 |  |  |
| R34 | G3P = G3P_B |  |  | B | 0.01004 | 0.000502 |  |  |
| R35 | 3PG = 3PG_B |  |  | B | 0.130677 | 0.006534 |  |  |
| R36 | PEP = PEP_B |  |  | B | 0.063513 | 0.003176 |  |  |
| R37 | PYR = PYR_B |  |  | B | 0.239538 | 0.011977 |  |  |
| R38 | ACCOA = ACCOA_B |  |  | B | 0.197311 | 0.009866 |  |  |
| R39 | OAA = OAA_B |  |  | B | 0.132215 | 0.006611 |  |  |
| R40 | GLU = GLU_B |  |  | B | 0.08036 | 0.004018 |  |  |
| R41 | PRO = PRO_B |  |  | B | 0.024004 | 0.0012 |  |  |
| R42 | ACCOA = ACE_EX |  |  | B | X |  |  |  |
| R43 | AKG = GLU | abcde = abcde |  | FR |  |  |  |  |
| R44 | GLU = AKG | abcde = abcde |  | R | X |  |  |  |
| R45 | GLU = PRO | abcde = abcde |  | FR |  |  |  |  |
| R46 | PRO = GLU | abcde = abcde |  | R | X |  |  |  |
| R47 | PRO_EX = PRO | abcde = abcde |  | F | X |  |  |  |
| R48 | PRO + AKG = HYP + SUC_II | abcde + fghij = abcde + fghij |  | F |  |  |  |  |
| R49 | SUC_II = 0.5 SUC + 0.5 SUC + CO2 | abcde = 0.5 bcde + 0.5 edcb + a |  | F |  |  |  |  |
| R50 | HYP = HYP_EX |  |  | B | X |  |  |  |
| R51 | PYR + PYR = VALX + CO2X | abc + def = abefc + d |  | S |  |  |  |  |
| R52 | E4P + PEP = SHKM | abcd + efg = efgabcd |  | S |  |  |  |  |
| R53 | SHKM + PEP = CHRM | abcdefg + hij = abcdefghij |  | S |  |  |  |  |
| R54 | CHRM = PHEX + CO2X | abcdefghij = hijbcdefg + a |  | S |  |  |  |  |
| R55 | CHRM = TYRX + CO2 | abcdefghij = hijbcdefg + a |  | S |  |  |  |  |
| R56 | PYR = ALAX | abc = abc |  | S |  |  |  |  |
| R57 | OAA = THRX | abcd = abcd |  | S |  |  |  |  |
| R58 | OAA = ASPX | abcd = abcd |  | S |  |  |  |  |
| R59 | GLU = GLUX | abcde = abcde |  | S |  |  |  |  |
| R60 | PRO = PROX | abcde = abcde |  | S |  |  |  |  |
| R61 | 3PG = SERX | abc = abc |  | S |  |  |  |  |
| R62 | 3PG = GLYX + MTHF | abc = ab + c |  | S |  |  |  |  |
| R63 | PEP + OAA = ILEX + CO2X | abc + defg = debfgc + a |  | S |  |  |  |  |
| R64 | PEP + PEP = ISV + CO2X | abc + def = abefc + d |  | S |  |  |  |  |
| R65 | ISV + ACCOA = LEUX + CO2X | abcde + fg = fgbcde + a |  | S |  |  |  |  |
|  |  |  |  |  |  |  |  |  |
| ## | excludedMetabolites |  |  |  |  |  |  |  |
| # | MTHF |  |  |  |  |  |  |  |
| # | GLC_EX |  |  |  |  |  |  |  |
| # | ACE_EX |  |  |  |  |  |  |  |
| # | PRO_EX |  |  |  |  |  |  |  |
| # | CO2 |  |  |  |  |  |  |  |
| # | CARB_EX_1 |  |  |  |  |  |  |  |
| # | CARB_EX_2 |  |  |  |  |  |  |  |
| # | HYP_EX |  |  |  |  |  |  |  |
| # | GLC6P_B |  |  |  |  |  |  |  |
| # | F6P_B |  |  |  |  |  |  |  |
| # | P5P_B |  |  |  |  |  |  |  |
| # | E4P_B |  |  |  |  |  |  |  |
| # | G3P_B |  |  |  |  |  |  |  |
| # | 3PG_B |  |  |  |  |  |  |  |
| # | PEP_B |  |  |  |  |  |  |  |
| # | PYR_B |  |  |  |  |  |  |  |
| # | ACCOA_B |  |  |  |  |  |  |  |
| # | OAA_B |  |  |  |  |  |  |  |
| # | GLU_B |  |  |  |  |  |  |  |
| # | PRO_B |  |  |  |  |  |  |  |
|  |  |  |  |  |  |  |  |  |
| ## | simulatedMDVs |  |  |  |  |  |  |  |
| # | ALAX#111 |  |  |  |  |  |  |  |
| # | ALAX#011 |  |  |  |  |  |  |  |
| # | GLYX#11 |  |  |  |  |  |  |  |
| # | GLYX#01 |  |  |  |  |  |  |  |
| # | VALX#11111 |  |  |  |  |  |  |  |
| # | VALX#01111 |  |  |  |  |  |  |  |
| # | LEUX#011111 |  |  |  |  |  |  |  |
| # | ILEX#011111 |  |  |  |  |  |  |  |
| # | PROX#01111 |  |  |  |  |  |  |  |
| # | SERX#111 |  |  |  |  |  |  |  |
| # | SERX#011 |  |  |  |  |  |  |  |
| # | THRX#1111 |  |  |  |  |  |  |  |
| # | THRX#0111 |  |  |  |  |  |  |  |
| # | PHEX#111111111 |  |  |  |  |  |  |  |
| # | PHEX#011111111 |  |  |  |  |  |  |  |
| # | ASPX#1111 |  |  |  |  |  |  |  |
| # | GLUX#11111 |  |  |  |  |  |  |  |
| # | TYRX#111111111 |  |  |  |  |  |  |  |
|  |  |  |  |  |  |  |  |  |
| ## | inputSubstrates |  |  |  |  |  |  |  |
| # | CARB_EX_1 |  |  |  |  |  |  |  |
| # | CARB_EX_2 |  |  |  |  |  |  |  |
| # | GLC_EX |  |  |  |  |  |  |  |
| # | PRO_EX |  |  |  |  |  |  |  |
|  |  |  |  |  |  |  |  |  |
| ## | measurements |  |  |  |  |  |  |  |
| # | 0.35861 | Ala 260 | m |  |  |  |  |  |
| # | 0.35119 |  | m+1 |  |  |  |  |  |
| # | 0.12234 |  | m+2 |  |  |  |  |  |
| # | 0.37797 | Ala 232 | m |  |  |  |  |  |
| # | 0.37403 |  | m+1 |  |  |  |  |  |
| # | 0.248 |  | m+2 |  |  |  |  |  |
| # | 0.61989 | Gly 246 | m |  |  |  |  |  |
| # | 0.17571 |  | m+1 |  |  |  |  |  |
| # | 0.69183 | Gly 218 | m |  |  |  |  |  |
| # | 0.30817 |  | m+1 |  |  |  |  |  |
| # | 0.1652 | Val 288 | m |  |  |  |  |  |
| # | 0.27676 |  | m+1 |  |  |  |  |  |
| # | 0.23638 |  | m+2 |  |  |  |  |  |
| # | 0.17336 | Val 260 | m |  |  |  |  |  |
| # | 0.28728 |  | m+1 |  |  |  |  |  |
| # | 0.29239 |  | m+2 |  |  |  |  |  |
| # | 0.10447 | Leu 200 | m |  |  |  |  |  |
| # | 0.23466 |  | m+1 |  |  |  |  |  |
| # | 0.29402 |  | m+2 |  |  |  |  |  |
| # | 0.12812 | Ile 200 | m |  |  |  |  |  |
| # | 0.25305 |  | m+1 |  |  |  |  |  |
| # | 0.28967 |  | m+2 |  |  |  |  |  |
| # | 0.81957 | Pro 184 | m |  |  |  |  |  |
| # | 0.13563 |  | m+1 |  |  |  |  |  |
| # | 0.03859 |  | m+2 |  |  |  |  |  |
| # | 0.30969 | Ser 390 | m |  |  |  |  |  |
| # | 0.35412 |  | m+1 |  |  |  |  |  |
| # | 0.16934 |  | m+2 |  |  |  |  |  |
| # | 0.33509 | Ser 362 | m |  |  |  |  |  |
| # | 0.39942 |  | m+1 |  |  |  |  |  |
| # | 0.2655 |  | m+2 |  |  |  |  |  |
| # | 0.17614 | Thr 404 | m |  |  |  |  |  |
| # | 0.29345 |  | m+1 |  |  |  |  |  |
| # | 0.25462 |  | m+2 |  |  |  |  |  |
| # | 0.22088 | Thr 376 | m |  |  |  |  |  |
| # | 0.3435 |  | m+1 |  |  |  |  |  |
| # | 0.28434 |  | m+2 |  |  |  |  |  |
| # | 0.10759 | Phe 336 | m |  |  |  |  |  |
| # | 0.19772 |  | m+1 |  |  |  |  |  |
| # | 0.19207 |  | m+2 |  |  |  |  |  |
| # | 0.12422 | Phe 234 | m |  |  |  |  |  |
| # | 0.21497 |  | m+1 |  |  |  |  |  |
| # | 0.23464 |  | m+2 |  |  |  |  |  |
| # | 0.17898 | Asp 418 | m |  |  |  |  |  |
| # | 0.29108 |  | m+1 |  |  |  |  |  |
| # | 0.25735 |  | m+2 |  |  |  |  |  |
| # | 0.10505 | Glu 432 | m |  |  |  |  |  |
| # | 0.22578 |  | m+1 |  |  |  |  |  |
| # | 0.27154 |  | m+2 |  |  |  |  |  |
| # | 0.09358 | Tyr 466 | m |  |  |  |  |  |
| # | 0.18454 |  | m+1 |  |  |  |  |  |
| # | 0.19173 |  | m+2 |  |  |  |  |  |
|  |  |  |  |  |  |  |  |  |
| ## | error |  |  |  |  |  |  |  |
| # | 0.00175 | Ala 260 | m |  |  |  |  |  |
| # | 0.00008 |  | m+1 |  |  |  |  |  |
| # | 0.00114 |  | m+2 |  |  |  |  |  |
| # | 0.00029 | Ala 232 | m |  |  |  |  |  |
| # | 0.00062 |  | m+1 |  |  |  |  |  |
| # | 0.00034 |  | m+2 |  |  |  |  |  |
| # | 0.00111 | Gly 246 | m |  |  |  |  |  |
| # | 0.00166 |  | m+1 |  |  |  |  |  |
| # | 0.00019 | Gly 218 | m |  |  |  |  |  |
| # | 0.00019 |  | m+1 |  |  |  |  |  |
| # | 0.00104 | Val 288 | m |  |  |  |  |  |
| # | 0.0007 |  | m+1 |  |  |  |  |  |
| # | 0.00121 |  | m+2 |  |  |  |  |  |
| # | 0.00009 | Val 260 | m |  |  |  |  |  |
| # | 0.00128 |  | m+1 |  |  |  |  |  |
| # | 0.00054 |  | m+2 |  |  |  |  |  |
| # | 0.00081 | Leu 200 | m |  |  |  |  |  |
| # | 0.00108 |  | m+1 |  |  |  |  |  |
| # | 0.00053 |  | m+2 |  |  |  |  |  |
| # | 0.0008 | Ile 200 | m |  |  |  |  |  |
| # | 0.00108 |  | m+1 |  |  |  |  |  |
| # | 0.00026 |  | m+2 |  |  |  |  |  |
| # | 0.00456 | Pro 184 | m |  |  |  |  |  |
| # | 0.00287 |  | m+1 |  |  |  |  |  |
| # | 0.00096 |  | m+2 |  |  |  |  |  |
| # | 0.00037 | Ser 390 | m |  |  |  |  |  |
| # | 0.00041 |  | m+1 |  |  |  |  |  |
| # | 0.00012 |  | m+2 |  |  |  |  |  |
| # | 0.00169 | Ser 362 | m |  |  |  |  |  |
| # | 0.00069 |  | m+1 |  |  |  |  |  |
| # | 0.001 |  | m+2 |  |  |  |  |  |
| # | 0.00051 | Thr 404 | m |  |  |  |  |  |
| # | 0.004 |  | m+1 |  |  |  |  |  |
| # | 0.00062 |  | m+2 |  |  |  |  |  |
| # | 0.00014 | Thr 376 | m |  |  |  |  |  |
| # | 0.00134 |  | m+1 |  |  |  |  |  |
| # | 0.00051 |  | m+2 |  |  |  |  |  |
| # | 0.00045 | Phe 336 | m |  |  |  |  |  |
| # | 0.00144 |  | m+1 |  |  |  |  |  |
| # | 0.00072 |  | m+2 |  |  |  |  |  |
| # | 0.00044 | Phe 234 | m |  |  |  |  |  |
| # | 0.00067 |  | m+1 |  |  |  |  |  |
| # | 0.00105 |  | m+2 |  |  |  |  |  |
| # | 0.0002 | Asp 418 | m |  |  |  |  |  |
| # | 0.00219 |  | m+1 |  |  |  |  |  |
| # | 0.00109 |  | m+2 |  |  |  |  |  |
| # | 0.00049 | Glu 432 | m |  |  |  |  |  |
| # | 0.0004 |  | m+1 |  |  |  |  |  |
| # | 0.00158 |  | m+2 |  |  |  |  |  |
| # | 0.00329 | Tyr 466 | m |  |  |  |  |  |
| # | 0.00031 |  | m+1 |  |  |  |  |  |
| # | 0.00073 |  | m+2 |  |  |  |  |  |

**The following was copied into each individual preSolverScript.m, replacing all prior contents**

**BL21(DE3) (pLysS) (pET-24a) grown on glucose**

%acetate

lb(9)=0.251-0.021;

ub(9)=0.251+0.021;

**BL21 *ΔputA* (DE3) (pLysS) (pET_24a) grown on glucose**

%acetate

lb(9)=0.189-0.024;

ub(9)=0.189+0.024;

**BL21(DE3) (pLysS) (pET_p4h1of) grown on glucose**

%acetate

lb(10)=0.192-0.01;

ub(10)=0.192+0.01;

%hyp

lb(8)=0.018-0.002;

ub(8)=0.018+0.002;

**BL21 *ΔputA* (DE3) (pLysS) (pET_p4h1of) grown on glucose**

%acetate

lb(10)=0.233-0.008;

ub(10)=0.233+0.008;

%hyp

lb(8)=0.029-0.001;

ub(8)=0.029+0.001;

**for BL21(DE3) (pLysS) (pET-24a) grown on glucose and proline**

%acetate

lb(12)=0.314-0.034;

ub(12)=0.314+0.034;

%pro

lb(10)=0.212-0.001;

ub(10)=0.212+0.001;

**for BL21 *ΔputA* (DE3) (pLysS) (pET-24a) grown on glucose and proline**

%acetate

lb(12)=0.151-0.011;

ub(12)=0.151+0.011;

%glu

lb(6)=0;

ub(6)=0;

**for BL21(DE3) (pLysS) (p4h1of) grown on glucose and proline**

%acetate

lb(12)=0.279-0.017;

ub(12)=0.279+0.017;

%pro

lb(10)=0.153-0.001;

ub(10)=0.153+0.001;

%hyp

lb(13)=0.084-0.001;

ub(13)=0.084+0.001;

**for BL21 *ΔputA* (DE3) (pLysS) (p4h1of) grown on glucose and proline**

%acetate

lb(12)=0.235-0.010;

ub(12)=0.235+0.010;

%pro

lb(10)=0.191-0.001;

ub(10)=0.191+0.001;

%hyp

lb(13)=0.191-0.001;

ub(13)=0.191+0.001;

**Comparison between experimental and simulated MDVs for the strains grown on glucose**

|  |  | **wt_pET24a** | | ***ΔputA*_pET24a** | | **wt_p4h1of** | | ***ΔputA*_p4h1of** | |
| --- | --- | --- | --- | --- | --- | --- | --- | --- | --- |
|  |  | **experimental** | **simulated** | **experimental** | **simulated** | **experimental** | **simulated** | **experimental** | **simulated** |
| Ala 260 | m | 0.366 | 0.363 | 0.366 | 0.363 | 0.365 | 0.364 | 0.361 | 0.359 |
|  | m+1 | 0.346 | 0.340 | 0.346 | 0.339 | 0.347 | 0.339 | 0.349 | 0.341 |
|  | m+2 | 0.120 | 0.126 | 0.121 | 0.127 | 0.121 | 0.126 | 0.122 | 0.129 |
| Ala 232 | m | 0.389 | 0.396 | 0.391 | 0.396 | 0.387 | 0.396 | 0.384 | 0.392 |
|  | m+1 | 0.365 | 0.353 | 0.364 | 0.353 | 0.367 | 0.354 | 0.369 | 0.357 |
|  | m+2 | 0.246 | 0.250 | 0.245 | 0.250 | 0.246 | 0.250 | 0.247 | 0.251 |
| Gly 246 | m | 0.622 | 0.607 | 0.621 | 0.606 | 0.621 | 0.603 | 0.619 | 0.602 |
|  | m+1 | 0.174 | 0.180 | 0.175 | 0.181 | 0.175 | 0.183 | 0.177 | 0.184 |
| Gly 218 | m | 0.692 | 0.673 | 0.693 | 0.673 | 0.692 | 0.671 | 0.693 | 0.671 |
|  | m+1 | 0.308 | 0.327 | 0.307 | 0.327 | 0.308 | 0.329 | 0.307 | 0.329 |
| Val 288 | m | 0.175 | 0.167 | 0.176 | 0.167 | 0.174 | 0.167 | 0.169 | 0.163 |
|  | m+1 | 0.278 | 0.272 | 0.278 | 0.272 | 0.277 | 0.272 | 0.278 | 0.272 |
|  | m+2 | 0.232 | 0.235 | 0.230 | 0.235 | 0.233 | 0.235 | 0.233 | 0.237 |
| Val 260 | m | 0.183 | 0.178 | 0.184 | 0.178 | 0.182 | 0.178 | 0.179 | 0.174 |
|  | m+1 | 0.286 | 0.283 | 0.287 | 0.283 | 0.286 | 0.283 | 0.287 | 0.283 |
|  | m+2 | 0.289 | 0.296 | 0.289 | 0.296 | 0.289 | 0.296 | 0.291 | 0.297 |
| Leu 200 | m | 0.113 | 0.100 | 0.114 | 0.100 | 0.113 | 0.100 | 0.109 | 0.097 |
|  | m+1 | 0.243 | 0.246 | 0.241 | 0.246 | 0.237 | 0.246 | 0.240 | 0.244 |
|  | m+2 | 0.292 | 0.295 | 0.290 | 0.295 | 0.290 | 0.295 | 0.293 | 0.296 |
| Ile 200 | m | 0.140 | 0.137 | 0.140 | 0.136 | 0.137 | 0.134 | 0.135 | 0.131 |
|  | m+1 | 0.260 | 0.264 | 0.258 | 0.264 | 0.255 | 0.263 | 0.259 | 0.262 |
|  | m+2 | 0.287 | 0.284 | 0.285 | 0.285 | 0.286 | 0.291 | 0.287 | 0.292 |
| Ser 390 | m | 0.319 | 0.327 | 0.321 | 0.327 | 0.313 | 0.326 | 0.313 | 0.323 |
|  | m+1 | 0.349 | 0.340 | 0.348 | 0.339 | 0.353 | 0.339 | 0.351 | 0.340 |
|  | m+2 | 0.167 | 0.154 | 0.167 | 0.155 | 0.169 | 0.157 | 0.170 | 0.158 |
| Ser 362 | m | 0.346 | 0.362 | 0.346 | 0.362 | 0.342 | 0.361 | 0.340 | 0.358 |
|  | m+1 | 0.392 | 0.360 | 0.392 | 0.359 | 0.395 | 0.361 | 0.396 | 0.363 |
|  | m+2 | 0.261 | 0.279 | 0.262 | 0.279 | 0.263 | 0.279 | 0.264 | 0.279 |
| Thr 404 | m | 0.193 | 0.194 | 0.190 | 0.193 | 0.187 | 0.192 | 0.184 | 0.190 |
|  | m+1 | 0.296 | 0.300 | 0.296 | 0.298 | 0.296 | 0.294 | 0.297 | 0.294 |
|  | m+2 | 0.247 | 0.238 | 0.248 | 0.241 | 0.248 | 0.244 | 0.250 | 0.245 |
| Thr 376 | m | 0.237 | 0.239 | 0.232 | 0.237 | 0.231 | 0.233 | 0.229 | 0.231 |
|  | m+1 | 0.343 | 0.339 | 0.343 | 0.339 | 0.343 | 0.338 | 0.345 | 0.339 |
|  | m+2 | 0.273 | 0.268 | 0.277 | 0.271 | 0.279 | 0.280 | 0.277 | 0.282 |
| Phe 336 | m | 0.111 | 0.119 | 0.111 | 0.119 | 0.113 | 0.119 | 0.109 | 0.116 |
|  | m+1 | 0.195 | 0.205 | 0.194 | 0.205 | 0.196 | 0.205 | 0.196 | 0.205 |
|  | m+2 | 0.192 | 0.188 | 0.192 | 0.188 | 0.190 | 0.188 | 0.191 | 0.189 |
| Phe 234 | m | 0.126 | 0.140 | 0.125 | 0.140 | 0.128 | 0.140 | 0.124 | 0.137 |
|  | m+1 | 0.211 | 0.225 | 0.212 | 0.225 | 0.214 | 0.225 | 0.215 | 0.226 |
|  | m+2 | 0.231 | 0.235 | 0.232 | 0.235 | 0.233 | 0.235 | 0.233 | 0.235 |
| Asp 418 | m | 0.194 | 0.194 | 0.194 | 0.193 | 0.191 | 0.192 | 0.187 | 0.189 |
|  | m+1 | 0.300 | 0.300 | 0.295 | 0.297 | 0.294 | 0.293 | 0.296 | 0.294 |
|  | m+2 | 0.246 | 0.238 | 0.249 | 0.241 | 0.250 | 0.244 | 0.253 | 0.245 |
| Glu 432 | m | 0.115 | 0.108 | 0.115 | 0.107 | 0.113 | 0.105 | 0.112 | 0.103 |
|  | m+1 | 0.234 | 0.228 | 0.234 | 0.227 | 0.231 | 0.226 | 0.233 | 0.225 |
|  | m+2 | 0.273 | 0.272 | 0.269 | 0.273 | 0.269 | 0.277 | 0.271 | 0.278 |
| Tyr 466 | m | 0.098 | 0.102 | 0.096 | 0.102 | 0.099 | 0.103 | 0.096 | 0.100 |
|  | m+1 | 0.181 | 0.189 | 0.183 | 0.189 | 0.182 | 0.189 | 0.185 | 0.189 |
|  | m+2 | 0.190 | 0.187 | 0.192 | 0.187 | 0.191 | 0.187 | 0.191 | 0.188 |
|  |  |  |  |  |  |  |  |  |  |

**Comparison between experimental and simulated MDVs for the strains grown on glucose and proline**

|  |  | **wt_pET24a** | | ***ΔputA*_pET24a** | | **wt_p4h1of** | | ***ΔputA*_p4h1of** | |
| --- | --- | --- | --- | --- | --- | --- | --- | --- | --- |
|  |  | **experimental** | **simulated** | **experimental** | **simulated** | **experimental** | **simulated** | **experimental** | **simulated** |
| Ala 260 | m | 0.377 | 0.378 | 0.368 | 0.363 | 0.360 | 0.358 | 0.359 | 0.355 |
|  | m+1 | 0.343 | 0.336 | 0.347 | 0.340 | 0.349 | 0.344 | 0.351 | 0.343 |
|  | m+2 | 0.119 | 0.124 | 0.118 | 0.125 | 0.125 | 0.128 | 0.122 | 0.130 |
| Ala 232 | m | 0.400 | 0.408 | 0.390 | 0.395 | 0.381 | 0.389 | 0.378 | 0.390 |
|  | m+1 | 0.360 | 0.350 | 0.365 | 0.354 | 0.372 | 0.361 | 0.374 | 0.360 |
|  | m+2 | 0.239 | 0.242 | 0.245 | 0.251 | 0.246 | 0.250 | 0.248 | 0.250 |
| Gly 246 | m | 0.624 | 0.618 | 0.622 | 0.607 | 0.621 | 0.606 | 0.620 | 0.602 |
|  | m+1 | 0.172 | 0.174 | 0.173 | 0.180 | 0.175 | 0.182 | 0.176 | 0.183 |
| Gly 218 | m | 0.694 | 0.680 | 0.691 | 0.674 | 0.692 | 0.672 | 0.692 | 0.671 |
|  | m+1 | 0.306 | 0.320 | 0.309 | 0.326 | 0.308 | 0.328 | 0.308 | 0.329 |
| Val 288 | m | 0.183 | 0.179 | 0.175 | 0.166 | 0.169 | 0.161 | 0.165 | 0.161 |
|  | m+1 | 0.282 | 0.279 | 0.278 | 0.272 | 0.278 | 0.273 | 0.277 | 0.272 |
|  | m+2 | 0.229 | 0.233 | 0.231 | 0.235 | 0.235 | 0.238 | 0.236 | 0.238 |
| Val 260 | m | 0.193 | 0.189 | 0.183 | 0.177 | 0.175 | 0.171 | 0.173 | 0.173 |
|  | m+1 | 0.291 | 0.288 | 0.286 | 0.283 | 0.288 | 0.284 | 0.287 | 0.285 |
|  | m+2 | 0.285 | 0.292 | 0.290 | 0.297 | 0.291 | 0.298 | 0.292 | 0.298 |
| Leu 200 | m | 0.120 | 0.109 | 0.114 | 0.099 | 0.105 | 0.095 | 0.104 | 0.096 |
|  | m+1 | 0.250 | 0.253 | 0.241 | 0.245 | 0.235 | 0.243 | 0.235 | 0.244 |
|  | m+2 | 0.291 | 0.293 | 0.291 | 0.295 | 0.294 | 0.297 | 0.294 | 0.297 |
| Ile 200 | m | 0.225 | 0.201 | 0.140 | 0.135 | 0.157 | 0.150 | 0.128 | 0.125 |
|  | m+1 | 0.297 | 0.288 | 0.260 | 0.263 | 0.268 | 0.272 | 0.253 | 0.260 |
|  | m+2 | 0.263 | 0.268 | 0.286 | 0.287 | 0.283 | 0.288 | 0.290 | 0.291 |
| Pro 184 | m | 0.818 | 0.819 | 0.819 | 0.820 | 0.819 | 0.820 | 0.820 | 0.757 |
|  | m+1 | 0.137 | 0.137 | 0.136 | 0.137 | 0.137 | 0.137 | 0.136 | 0.153 |
|  | m+2 | 0.039 | 0.039 | 0.039 | 0.039 | 0.039 | 0.039 | 0.039 | 0.064 |
| Ser 390 | m | 0.322 | 0.335 | 0.321 | 0.326 | 0.311 | 0.322 | 0.310 | 0.321 |
|  | m+1 | 0.348 | 0.340 | 0.349 | 0.340 | 0.353 | 0.343 | 0.354 | 0.342 |
|  | m+2 | 0.164 | 0.149 | 0.166 | 0.154 | 0.170 | 0.157 | 0.169 | 0.158 |
| Ser 362 | m | 0.349 | 0.367 | 0.346 | 0.361 | 0.338 | 0.355 | 0.335 | 0.357 |
|  | m+1 | 0.388 | 0.358 | 0.391 | 0.360 | 0.397 | 0.365 | 0.399 | 0.364 |
|  | m+2 | 0.263 | 0.276 | 0.263 | 0.279 | 0.265 | 0.280 | 0.266 | 0.279 |
| Thr 404 | m | 0.331 | 0.306 | 0.190 | 0.193 | 0.232 | 0.228 | 0.176 | 0.179 |
|  | m+1 | 0.288 | 0.291 | 0.296 | 0.297 | 0.288 | 0.295 | 0.293 | 0.303 |
|  | m+2 | 0.206 | 0.200 | 0.247 | 0.241 | 0.240 | 0.226 | 0.255 | 0.246 |
| Thr 376 | m | 0.370 | 0.336 | 0.232 | 0.235 | 0.273 | 0.263 | 0.221 | 0.221 |
|  | m+1 | 0.318 | 0.323 | 0.345 | 0.339 | 0.334 | 0.337 | 0.344 | 0.342 |
|  | m+2 | 0.213 | 0.223 | 0.277 | 0.274 | 0.260 | 0.266 | 0.284 | 0.281 |
| Phe 336 | m | 0.114 | 0.127 | 0.110 | 0.118 | 0.109 | 0.116 | 0.108 | 0.115 |
|  | m+1 | 0.198 | 0.210 | 0.195 | 0.205 | 0.197 | 0.206 | 0.198 | 0.205 |
|  | m+2 | 0.190 | 0.184 | 0.191 | 0.188 | 0.192 | 0.190 | 0.192 | 0.190 |
| Phe 234 | m | 0.130 | 0.147 | 0.126 | 0.139 | 0.125 | 0.135 | 0.124 | 0.136 |
|  | m+1 | 0.216 | 0.226 | 0.213 | 0.225 | 0.215 | 0.227 | 0.215 | 0.227 |
|  | m+2 | 0.232 | 0.232 | 0.232 | 0.235 | 0.234 | 0.236 | 0.235 | 0.236 |
| Asp 418 | m | 0.336 | 0.306 | 0.194 | 0.192 | 0.233 | 0.228 | 0.179 | 0.179 |
|  | m+1 | 0.287 | 0.290 | 0.295 | 0.297 | 0.288 | 0.295 | 0.291 | 0.302 |
|  | m+2 | 0.205 | 0.200 | 0.249 | 0.241 | 0.241 | 0.226 | 0.257 | 0.246 |
| Glu 432 | m | 0.333 | 0.321 | 0.115 | 0.112 | 0.195 | 0.195 | 0.105 | 0.098 |
|  | m+1 | 0.256 | 0.248 | 0.232 | 0.227 | 0.236 | 0.235 | 0.226 | 0.223 |
|  | m+2 | 0.208 | 0.211 | 0.267 | 0.272 | 0.248 | 0.251 | 0.272 | 0.276 |
| Tyr 466 | m | 0.100 | 0.109 | 0.094 | 0.102 | 0.096 | 0.100 | 0.094 | 0.099 |
|  | m+1 | 0.184 | 0.194 | 0.183 | 0.189 | 0.184 | 0.190 | 0.185 | 0.189 |
|  | m+2 | 0.190 | 0.185 | 0.192 | 0.187 | 0.193 | 0.189 | 0.192 | 0.189 |
|  |  |  |  |  |  |  |  |  |  |
|  |  |  |  |  |  |  |  |  |  |

**Monte Carlo Analysis for the strains grown on glucose**

|  | wt_pET24a | | *ΔputA*_pET24a | | wt_p4h1of | | *ΔputA*_p4h1of | |
| --- | --- | --- | --- | --- | --- | --- | --- | --- |
|  | LB | UB | LB | UB | LB | UB | LB | UB |
| GLC6P ↔ F6P | 54 | 56 | 53 | 55 | 54 | 56 | 56 | 58 |
| F6P ↔ T3P+T3P | 77 | 78 | 77 | 78 | 78 | 79 | 79 | 80 |
| GLC6P ↔ P5P + CO2 | 42 | 44 | 43 | 45 | 43 | 45 | 40 | 43 |
| P5P + P5P ↔ S7P + T3P | 12.6 | 13.3 | 12.9 | 13.6 | 13.0 | 13.8 | 12.1 | 12.9 |
| S7P + T3P↔ E4P + F6P | 12.6 | 13.3 | 12.9 | 13.6 | 13.0 | 13.8 | 12.1 | 12.9 |
| E4P + P5P ↔ F6P + T3P | 9.5 | 10.2 | 10.0 | 10.7 | 10.4 | 11.1 | 10.1 | 10.9 |
| T3P ↔ 3PG | 163 | 164 | 163 | 164 | 167 | 168 | 168 | 169 |
| 3PG ↔ PEP | 150 | 150 | 151 | 152 | 155 | 156 | 158 | 159 |
| PEP = PYR | 117 | 118 | 122 | 123 | 129 | 130 | 133 | 134 |
| PYR = ACCOA + CO2 | 92 | 93 | 96 | 96 | 109 | 110 | 114 | 115 |
| ACCOA + OAA → CIT | 48 | 52 | 55 | 61 | 70 | 73 | 74 | 76 |
| CIT → AKG + CO2 | 48 | 52 | 55 | 61 | 70 | 73 | 74 | 76 |
| CIT = S_PREC + GLYOX | 0 | 0 | 0 | 0 | 0 | 0 | 0 | 0 |
| AKG = SUC + CO2 | 37 | 41 | 45 | 52 | 58 | 62 | 61 | 64 |
| SUC ↔ MAL | 37 | 41 | 45 | 52 | 60 | 64 | 64 | 66 |
| MAL = OAA | 37 | 41 | 45 | 52 | 60 | 64 | 64 | 66 |
| MAL = PYR + CO2 | 0 | 0 | 0 | 0 | 0 | 0 | 0 | 0 |
| PEP + CO2 = OAA | 25.8 | 25.8 | 22.8 | 22.8 | 20.7 | 21.1 | 19.6 | 19.7 |
| HYP = HYP_EX | 0 | 0 | 0 | 0 | 1.8 | 2.2 | 2.9 | 3.1 |
| *Monte Carlo analyses of the MFA data reported in Fig. 2. All fluxes are normalized to the glucose uptake rate (set to 100). The upper and lower boundaries (LB and UB) define the 95% confidence interval* | | | | | | | | |

**Monte Carlo Analysis for the strains grown on glucose and proline**

|  | wt_pET24a | | *ΔputA*_pET24a | | wt_p4h1of | | *ΔputA*_p4h1of | |
| --- | --- | --- | --- | --- | --- | --- | --- | --- |
|  | LB | UB | LB | UB | LB | UB | LB | UB |
| GLC6P ↔ F6P | 54 | 57 | 54 | 56 | 59 | 61 | 56 | 58 |
| F6P ↔ T3P+T3P | 76 | 77 | 76 | 77 | 79 | 80 | 78 | 78 |
| GLC6P ↔ P5P + CO2 | 42 | 44 | 43 | 45 | 38 | 40 | 40 | 42 |
| P5P + P5P ↔ S7P + T3P | 12 | 13 | 13 | 13 | 11 | 12 | 12 | 13 |
| S7P + T3P↔ E4P + F6P | 12 | 13 | 13 | 13 | 11 | 12 | 12 | 13 |
| E4P + P5P ↔ F6P + T3P | 9 | 9 | 9 | 10 | 9 | 9 | 9 | 9 |
| T3P ↔ 3PG | 160 | 161 | 162 | 162 | 167 | 168 | 164 | 165 |
| 3PG ↔ PEP | 145 | 145 | 149 | 149 | 154 | 154 | 151 | 152 |
| PEP = PYR | 109 | 120 | 117 | 119 | 133 | 133 | 123 | 124 |
| PYR = ACCOA + CO2 | 103 | 104 | 92 | 93 | 110 | 111 | 97 | 98 |
| ACCOA + OAA → CIT | 45 | 45 | 55 | 60 | 62 | 68 | 53 | 54 |
| CIT → AKG + CO2 | 45 | 45 | 55 | 60 | 62 | 68 | 53 | 54 |
| CIT = S_PREC + GLYOX | 0 | 0 | 0 | 0 | 0 | 0 | 0 | 0 |
| AKG = SUC + CO2 | 54 | 55 | 46 | 52 | 51 | 57 | 24 | 26 |
| SUC ↔ MAL | 54 | 55 | 46 | 52 | 59 | 65 | 43 | 45 |
| MAL = OAA | 32 | 42 | 46 | 52 | 59 | 65 | 43 | 45 |
| MAL = PYR + CO2 | 12 | 22 | 0 | 0 | 0 | 0 | 0 | 0 |
| PEP + CO2 = OAA | 18 | 28 | 23 | 24 | 15 | 15 | 22 | 22 |
| AKG = GLU | -9.0 | -9.0 | 8.0 | 8.5 | 2.7 | 2.7 | 7.1 | 7.1 |
| PRO = GLU | 18.5 | 18.6 | 0 | -0.24 | 4.9 | 4.9 | -2.3 | -2.3 |
| *Monte Carlo analyses of the MFA data reported in Fig. 2. All fluxes are normalized to the glucose uptake rate (set to 100). The upper and lower boundaries (LB and UB) define the 95% confidence interval* | | | | | | | | |

**Mass isotopomer distribution of the amino acids for the strains under conditions studied using 20% 1-^13^C and 80% U-^13^C labelled glucose**
